# CF_3_-substituted carbocations: underexploited intermediates with great potential in modern synthetic chemistry

**DOI:** 10.3762/bjoc.17.32

**Published:** 2021-02-03

**Authors:** Anthony J Fernandes, Armen Panossian, Bastien Michelet, Agnès Martin-Mingot, Frédéric R Leroux, Sébastien Thibaudeau

**Affiliations:** 1 Université de Strasbourg, Université de Haute-Alsace, CNRS, UMR 7042-LIMA, ECPM, 25 Rue Becquerel, 67087 Strasbourg, France; 2 Université de Poitiers, CNRS, IC2MP, UMR 7285, Equipe “Synthèse Organique”, 4 Rue Michel Brunet, 86073 Poitiers Cedex 9, France

**Keywords:** carbocation, organic synthesis, superelectrophile, trifluoromethyl

## Abstract

“The extraordinary instability of such an “ion” accounts for many of the peculiarities of organic reactions” – Franck C. Whitmore (1932). This statement from Whitmore came in a period where carbocations began to be considered as intermediates in reactions. Ninety years later, pointing at the strong knowledge acquired from the contributions of famous organic chemists, carbocations are very well known reaction intermediates. Among them, destabilized carbocations – carbocations substituted with electron-withdrawing groups – are, however, still predestined to be transient species and sometimes considered as exotic ones. Among them, the CF_3_-substituted carbocations, frequently suggested to be involved in synthetic transformations but rarely considered as affordable intermediates for synthetic purposes, have long been investigated. This review highlights recent and past reports focusing on their study and potential in modern synthetic transformations.

## Introduction

Carbocations are pivotal intermediates in organic chemistry, and carbocation-based synthetic chemistry continues to be a vital part of industrial and academic chemistry [1]. A countless number of carbocations have been generated and studied [2–3], and many famous organic chemists strongly participated in their development. Carbocations that are especially intriguing are the destabilized ones that have been elegantly reviewed over the past years by Gassman, Tidwell, and Creary [4–6]. The so-called electron-deficient carbocations, i.e., carbocations substituted with electron-withdrawing groups, drive original reactions, and the most important one among these cations is probably the α-(trifluoromethyl) carbocation. Many efforts are currently devoted to develop methods allowing the efficient insertion of fluorine atoms or fluorinated groups into organic molecules [7–12]. The increasing demand for fluorinated scaffolds, due to the striking beneficial effects generally resulting from the introduction of these fluorinated motifs [13], also participated in this development. These fluorine effects are nowadays remarkably established in many domains, including medicinal, organic, and organometallic chemistry, catalysis, chemical biology, and material sciences [14–17]. In this context, deciphering the impact that can be exerted by the trifluoromethyl group on a cation and the associated consequences when facing the challenge of developing innovative synthetic methods are the subjects of this review.

## Review

Quantitative parameters accounting for the electron-donating or -withdrawing ability of substituents are of major importance in synthetic organic chemistry. The Hammett constant σ for a variety of substituents [18–19] and improved values, known as σ^+^, furnished by Brown et al. [20–21] – some of which are listed in Table 1 for selected substituents – were developed towards this aim. Following this classification, the CF_3_ group is amongst the most electron-withdrawing substituents, with a σ_p_^+^ value of +0.612 for the *para*-position.

**Table 1:** Selection of Hammett constant σ^+^ values for selected functional groups X, extracted from References [20–21].

X	σ*^+^*	
	*meta*	*para*

NMe_2_	n.d.	−1.7
NH_2_	−0.16	−1.3
OH	+0.12^a^	−0.92
OMe	+0.047	−0.778
CH_3_	−0.066	−0.311
SiMe_3_	+0.011	+0.021
Ph	+0.109	−0.179
H	0	0
SMe	+0.158	−0.604
F	+0.352	−0.073
Cl	+0.399	+0.114
Br	+0.405	+0.150
I	+0.359	+0.135
NMe_3_^+^	+0.359	+0.408
CO_2_Et	+0.366	+0.482
C(O)Me	+0.38^a^	+0.50^a^
**CF_3_**	**+0.52**	**+0.612**
CN	+0.562	+0.659
NO_2_	+0.674	+0.790

^a^σ values based on benzoic acid ionization.

However, as noted by Reynolds et al. [22–23], “the electronic effect of a substituent depends to a certain extent upon the electron demand in the system to which it is attached”. Thus, despite the strong intrinsic electron-withdrawing character, the trifluoromethyl group was shown to modestly act as a π-electron donor when substituting a carbenium ion. Ab initio calculations were performed to account for the π-electron-donating ability of several substituents conjugated with carbocations (Table 2). It is noteworthy that amongst the several substituents studied, the CF_3_ group exhibits the lowest π-electron-donation ability in each investigated carbenium series, reflecting, as one could expect, the very poor stabilizing power by π-electron donation. A trend exists in the magnitude of the parameter according to the nature of the carbenium ions, which is in line with the carbenium ion stability (alkyl < allylic < benzylic). Thus, an increased π-electron transfer is present in the least-stabilized alkylcarbenium ions, in which a higher electronic contribution from neighboring substituents is required.

**Table 2:** π-Electron-transfer parameters from STO-3G calculations with optimized C–X bond length (established as ∑*q_π_*, without unit) for substituents X in alkyl, allylic, and benzylic carbenium ions. Parameters for neutral phenyl derivatives are given for comparison. Negative values indicate π-electron donation by the substituent [22–23].

X	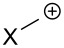	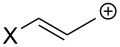	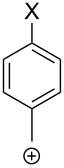	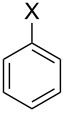

NH_2_	−566	−434	−284	−115
OH	−486	−334	−202	−90
CH=CH_2_	−427	−243	−148	0
F	−353	−223	−134	−70
CN	−262	−105	−33	+21
CHO	−155	−77	−20	+27
CH_3_	−113	−58	−29	−8
NO_2_	−76	−36	−10	+19
**CF_3_**	−**29**	−**15**	−**4**	**+10**
H	0	0	0	0

**Figure 1:**
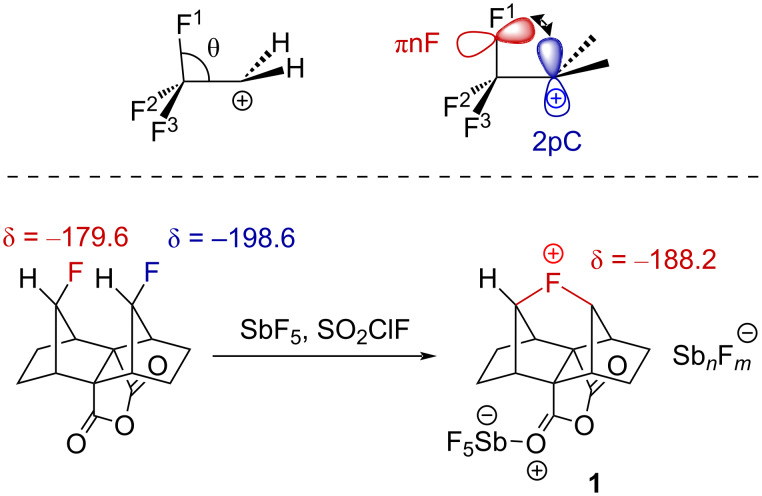
Stabilizing interaction in the CF_3_CH_2_^+^ carbenium ion (top) and structure of the first observable fluoronium ion **1** (bottom) (δ in ppm).

Detailed ab initio studies have been focused on the stability of the CF_3_CH_2_^+^ cation and provide pieces of thoughts on the origins of the stabilizing interactions in α-(trifluoromethyl)carbenium ions. The optimization of the geometry for CF_3_CH_2_^+^ at the STO-3G level led to an energy minimum, in which one of the fluorine atoms is significantly closer to the positive carbon center (Figure 1, top, θ = 101°) [24]. However, exactly the same structural distortion was calculated for the ethyl cation. Furthermore, the very small π-electron density calculated in the 2pC orbital of CF_3_CH_2_^+^ (0.04 electrons) led the authors to conclude that “there is no hyperconjugative stabilization by the CF_3_ group”. The presence of this attractive interaction should, however, not be discarded. Indeed, the quantitative PMO analysis at the 6-31G* level allowed, by calculating fragment orbitals (FO), the identification of the nature of this attractive interaction [25]. The latter arose from a homoconjugation interaction (−5.3 kcal⋅mol^−1^) of one fluorine lone pair (πnF FO) with the empty 2pC orbital of the cationic carbon center (Figure 1, top). A second stabilizing interaction was also found and came from hyperconjugation of the CF_3_ substituent, involving interactions between the empty 2pC orbital with the πCF_3_ FO (−5.2 kcal⋅mol^−1^). In 2018, spectroscopic evidence for the generation of the first observable fluoronium ion **1** by Letcka et al., which can be seen as a strong nF→2pC interaction (Wiberg bond order of 0.53 for each C–F bond), gave additional credit to these calculations (Figure 1, bottom) [26–28].

**Scheme 1:**
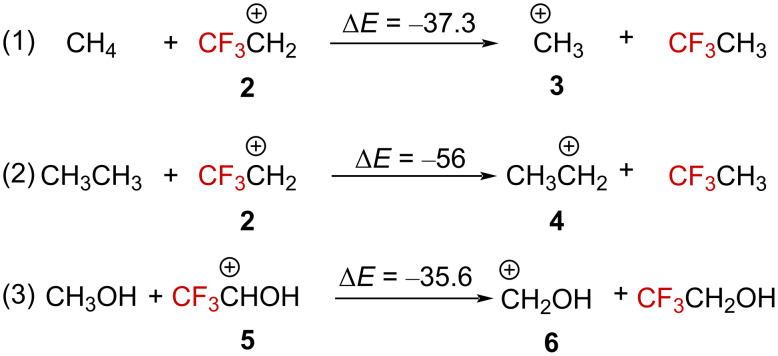
Isodesmic equations accounting for the destabilizing effect of the CF_3_ group. Δ*E* in kcal⋅mol^−1^, calculated at the 4-31G level.

The thermochemical data can also provide information on the effect of the CF_3_ group on the stability of the carbenium ions. Calculations of the isodesmic reactions (1), (2), and (3) demonstrate the overall destabilizing effect of CF_3_ compared to H or CH_3_ when directly attached to a carbenium ion (i.e., α position, Scheme 1) [5,29]. Even an oxonium ion appears to be significantly destabilized by the presence of the CF_3_ group. These data globally suggest, as one could expect, an electronic destabilizing effect of the CF_3_ group when attached closely to a carbenium ion. However, any strong nF→2pC interaction might also influence the overall stability of any system.

Any perspectives toward CF_3_-containing carbocation-based synthesis must take this trend into account, especially studies on the specific α-(trifluoromethyl)carbenium ions. This review aims to systematically relate the reported work in this field. For each part, a focus on a series of α-(trifluoromethyl)carbenium ions differing in its chemical environment will be scrutinized. The chapter will summarize kinetic studies and concomitant theoretical investigations on the cations formation and stability data as well as synthetic perspectives offered by the studied carbenium ions. Any discussion of the results coming from the ionization of perfluorinated substrates will not be addressed in this review [30–33].

### Aryl-substituted trifluoromethylated carbenium ions

**Scheme 2:**
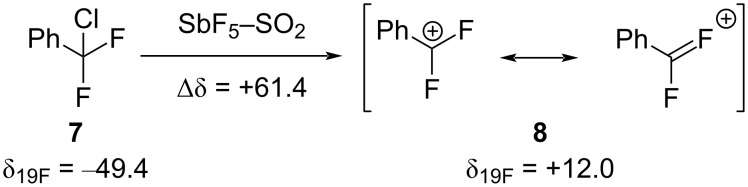
Stabilizing effect of fluorine atoms by resonance electron donation in carbenium ions (δ in ppm).

**α-(Trifluoromethyl)-substituted carbenium ions:** At the dawn of their outstanding studies on carbocation chemistry, Olah et al. empirically demonstrated that despite exhibiting the highest Pauling electronegativity, the fluorine atoms, when directly linked to a carbenium ion, can be engaged in significant resonance electron donation (Scheme 2) [34]. While stabilizing the positively charged carbon center via lone pair conjugation, the electron density at the fluorine atom decreases, and this phenomenon is shown by a large downfield shift in the ^19^F NMR spectrum of **8** compared to the neutral precursor **7**.

**Scheme 3:**
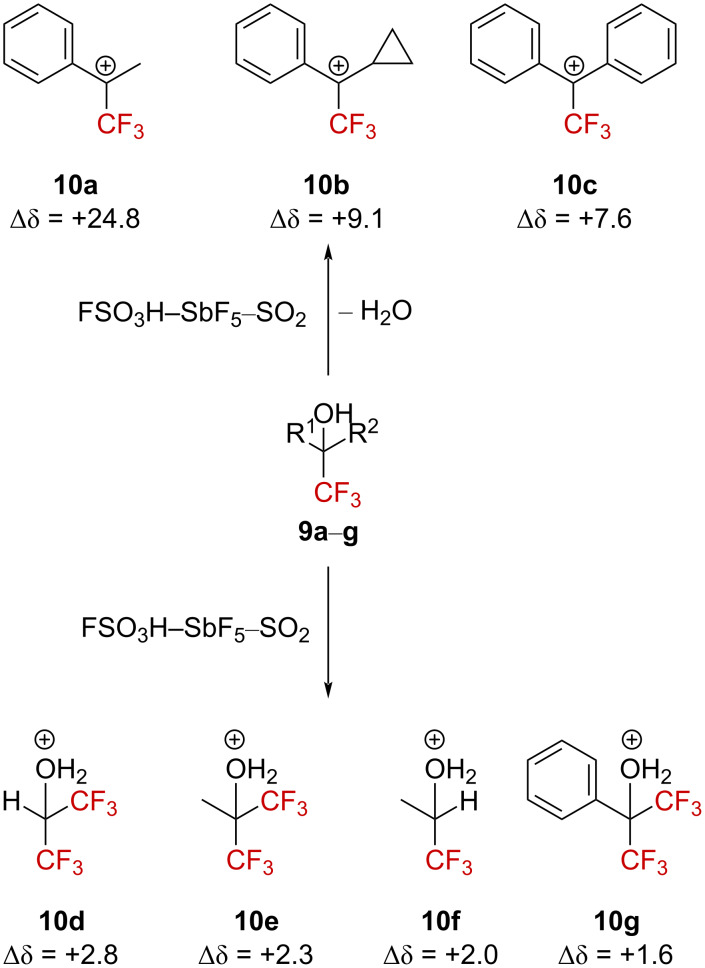
Direct in situ NMR observation of α-(trifluoromethyl)carbenium ion or protonated alcohols. Δδ = δ_19F,product_ − δ_19F,precursor_ (δ in ppm).

Following these studies on the evaluation of fluorine atom(s) substitution on cation behavior, Olah et al. then investigated the expected destabilizing effect resulting from the presence of fluorine atoms close to a carbenium ion [35]. Thus, Olah et al. envisioned the possibility to generate α-(trifluoromethyl)carbenium ions, and this achievement led to the first direct observation of these species using low-temperature NMR experiments in situ [35]. In this study, the authors furnished spectroscopic evidence for the complete ionization of several α-(trifluoromethyl) alcohol precursors **9a**–**c** in a superacidic FSO_3_H–SbF_5_–SO_2_ medium. They also brought experimental ^19^F NMR variation values up to Δδ = +24.8 ppm (Scheme 3). This suggests a partial stabilization of the cationic center by hyperconjugation and/or fluorine lone pair interaction, resulting in a certain degree of a positive charge of one fluorine atom. Interestingly, at least one phenyl substituent was required to allow the ionization of the starting alcohols into the corresponding carbenium ions. When the aromatic substituent was absent or upon installation of an additional CF_3_ group, only the corresponding protonated alcohols **10d**–**g** were observed.

**Scheme 4:**
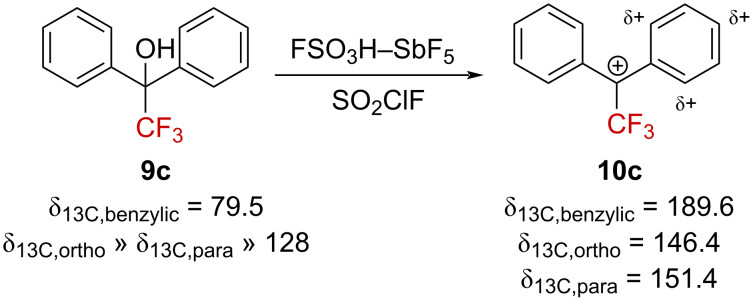
Reported ^13^C NMR chemical shifts for the α-(trifluoromethyl)carbenium ion **10c** (δ in ppm).

Olah et al. also reported the ^13^C NMR chemical shifts for carbenium ion **10c** upon ionization of the alcohol precursor **9c** in a superacid (Scheme 4) [36]. A large downfield shift was observed predominantly at the benzylic position (Δδ_13C_ = 110.1 ppm), with minor impacts at the *ortho*- and *para*-positions (Δδ_13C_ ≈ 20 ppm) relative to the starting alcohol **9c** [37]. These variations are fully consistent with the presence of a positive charge located at the benzylic position, with only partial stabilization of the cationic center by the phenyl groups.

**Scheme 5:**
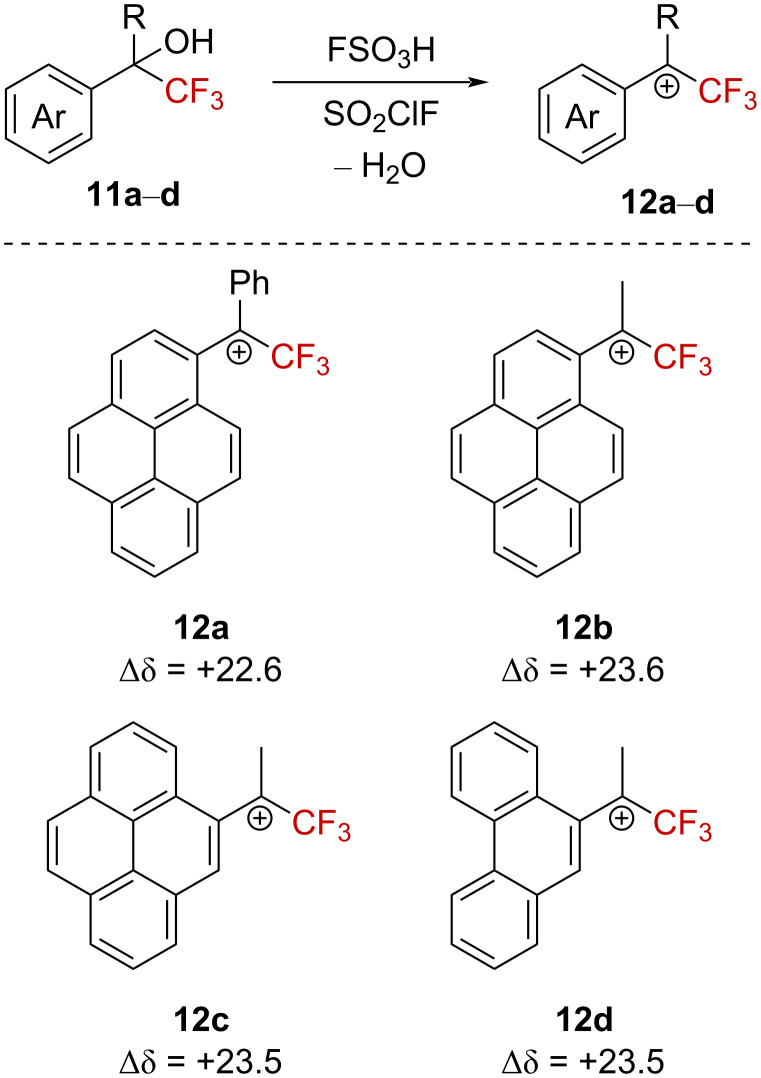
Direct NMR observation of α-(trifluoromethyl)carbenium ions in situ (δ in ppm).

Similarly, Laali et al. observed significant ^19^F NMR downfield chemical shifts upon the formation of α-(trifluoromethyl)pyrenylcarbenium- and α-(trifluoromethyl)anthracenylcarbenium ions **12a**–**d** from the corresponding carbinols **11a**–**d** (Scheme 5) [38].

**Scheme 6:**
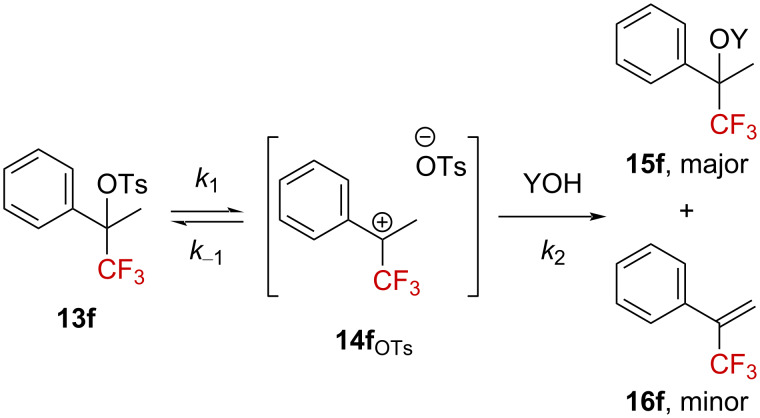
Illustration of the ion pair solvolysis mechanism for sulfonate **13f**. YOH = solvent.

Tidwell et al. explored the influence of a CF_3_ group on the solvolysis reaction of various benzylic sulfonate derivatives [39–40]. They found a linear free-energy relationship between the solvolysis rate of sulfonate **13f** in different solvents compared to the one of 2-adamantyl tosylate, the latter being known to undergo solvolysis via the formation of a carbenium ion. Hence, the formation of a highly destabilized α-(trifluoromethyl)carbenium ion **14f**_OTs_ was established as the rate-limiting step in the solvolysis reactions of **13f** (Scheme 6). Furthermore, the authors determined a *k*_CH3_/*k*_CD3_ ratio of 1.54, highlighting an isotopic effect consistent with a solvolysis mechanism involving a carbenium ion (*k*_CH3_/*k*_CD3_ = 1.48 for 2-methyl-2-adamantyl tosylate). Also, *k*_H_/*k*_CF3_ = 2⋅10^5^ was established, illustrating the retarding α-CF_3_ effect in the production of a carbenium ion [41]. In the solvolysis reaction of **13f**, a mixture of the major product **15f**, resulting from solvent substitution, and the minor elimination product **16f** was observed. Further, ^14^C labeling experiments on **13f** confirmed that the formation of the ion pair **14f**_OTs_ was a reversible process [42].

**Figure 2:**
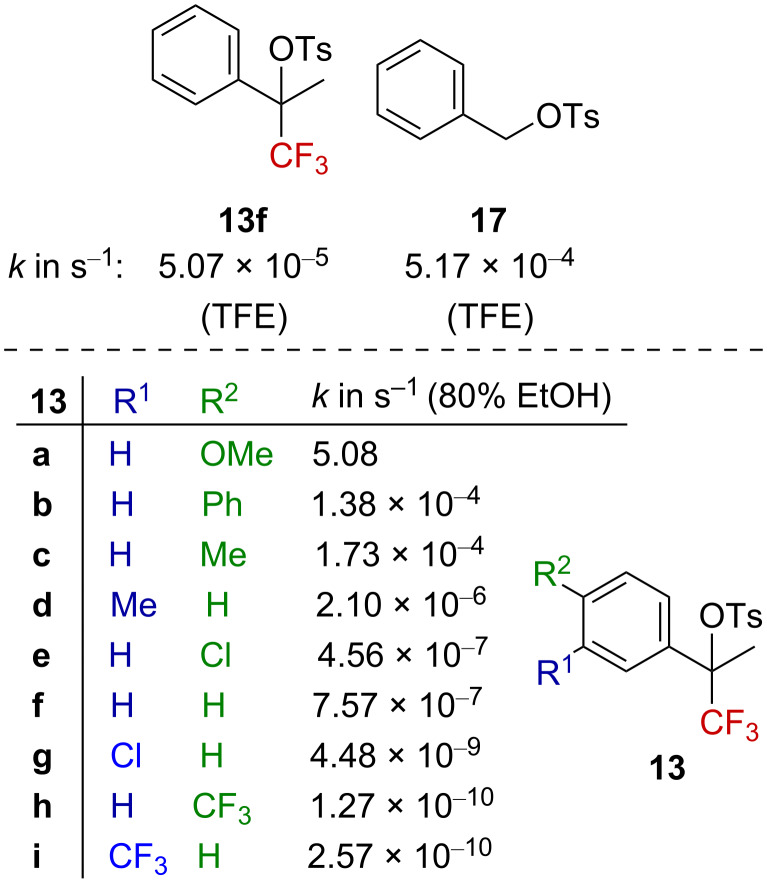
Solvolysis rate for **13a**–**i** and **17**.

Later, Liu et al. explored the solvolysis of aryl derivatives **13a**–**i** to highlight the importance of the nature of the aromatic substituent on the solvolysis rate (Figure 2) [43]. As anticipated, a faster rate was observed for electron-donating groups, while electron-withdrawing groups slowed the process down. Plotting the Hammett–Brown correlation, established as log(*k*) = *f*(σ^+^), gave a linear dependence of the rate with the σ^+^ parameters of the aryl substituents, with a behavior in agreement with the transient formation of a carbenium ion. The slope of the straight line, ρ^+^ = −7.46, reflects the very high electron demand induced by the CF_3_ group. Remarkably, they found that CF_3_ deactivates to such an extent that benzylic tosylate **13f** was approximately 10 times less reactive than benzylic tosylate **17** (Figure 2, top). Similarly to the previous study, the Grunwald–Winstein plot [44] gave a linear free-energy relationship between the solvolysis rate for derivatives **13f** or **13g** and the solvent polarity parameter *Y*_OTs_ [45]. The solvent participation in the solvolysis of these tertiary benzylic tosylates was thus defined as “unimportant” by the authors.

**Figure 3:**
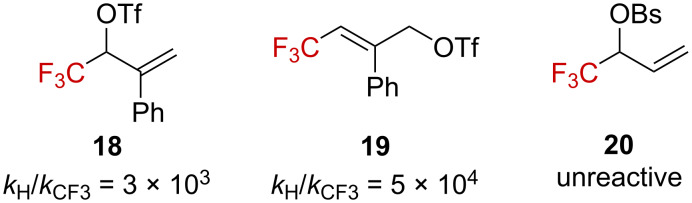
Structures of allyl triflates **18** and **19** and allyl brosylate **20**. Bs = *p*-BrC_6_H_4_SO_2_.

Gassman and Harrington successfully measured the solvolysis kinetics of CF_3_-substituted allylic triflates **18** and **19**, showing a significant solvolysis retardation with CF_3_-substituted substrates (Figure 3) [46]. These results are in accordance with an earlier study that revealed that **20** was unreactive in acetone/H_2_O 70:30, even over a period of 35 days at 50 °C [47].

**Figure 4:**
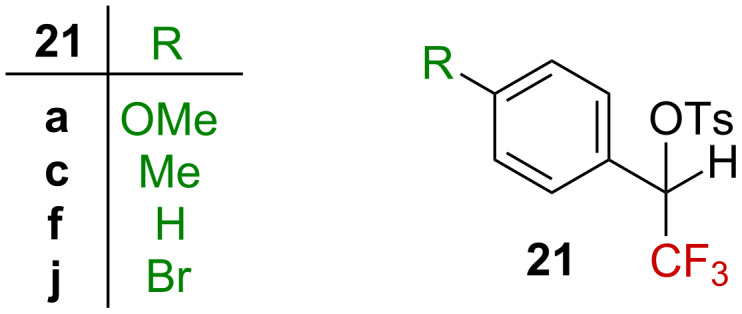
Structure of tosylate derivatives **21**.

Encouraged by these preliminary results, Tidwell et al. envisioned the possibility to study the solvolysis reaction of secondary benzylic sulfonates [48]. In tertiary benzylic sulfonates [39,43], a linear free-energy relationship between the solvolysis rate for the secondary benzylic tosylates **21** (Figure 4) and *Y*_OTs_ was obtained. Similarly, the nature of the aromatic substituent influenced the solvolysis rate, with an observed acceleration for substrates adorned with electron donor substituents and a deceleration for those carrying electron-withdrawing substituents. The Hammett–Brown correlation gave a straight line, with ρ^+^ = −10.1 (80% EtOH, 25 °C), a significantly greater magnitude than for the tertiary derivatives (−7.46), in agreement with the transient formation of a more destabilized carbenium ion (i.e., a secondary carbenium ion). They also noticed that the greatest magnitude of ρ^+^ was obtained in the most nucleophilic and less ionizing solvents, in agreement with an increased electron demand on the aromatic substituent in a poorly ionizing solvent. This also suggests that the positive charge is delocalized to a higher extent on the aromatic substituent for the secondary tosylates than for the tertiary ones. These data support the hypothesis that the transient formation of a carbenium ion is the rate-limiting step and the absence of significant solvent participation in the latter. Richard also conducted extensive studies on the impact of the nature of the leaving group (I, Br, OSO_2_R, etc.) and on the aryl substituents (NMe_2_, OMe, SMe, etc.) in the derivatives **21**, substituted with a secondary CF_3_ group in the benzylic position, and reported similar conclusions [49–50].

**Figure 5:**
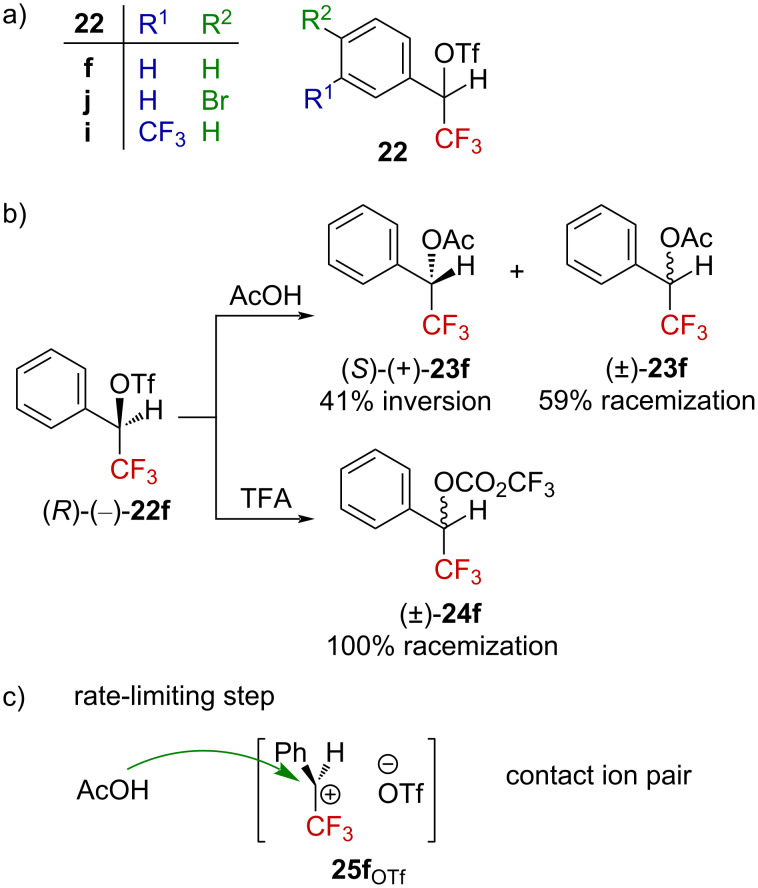
a) Structure of triflate derivatives **22**. b) Stereochemistry outcomes of the reaction starting from (*R*)-(−)-**22f**. c) Rate-limiting step in poorly ionizing solvents.

A different behavior emerged from triflate derivatives **22** (Figure 5a). In addition to their enhanced reactivity (*k*_Tf_/*k*_Ts_ = 2 × 10^4^), a nonlinear free-energy relationship between the solvolysis rate and *Y*_OTs_ was obtained, suggesting an important solvent participation in these cases. Further investigations on **22f** showed deuterium isotope effects in agreement with the transient formation of a carbenium ion. A solvent dependence of the *k*_H_/*k*_D_ ratio was also noticed, with the higher ratios being obtained in the most ionizing and less nucleophilic solvents (i.e., 1.34 ± 0.07 in HFIP vs 1.21 ± 0.01 in 80% EtOH). The subsequent solvolysis of enantioenriched triflate (*R*)-(−)-**22f** evidenced that in a poorly ionizing solvent, such as AcOH, solvolysis occurred with 41% inversion (and 59% racemization, i.e., product **23f** was obtained with an enantiomeric ratio of ca. 70:30 in favor of the (*S*)-enantiomer), while complete racemization was observed in more ionizing TFA or HFIP as the solvent (Figure 5b) [48]. These observations are in agreement with a process generating a carbenium ion in highly ionizing solvents (TFA, HFIP, etc.) for the tosylates derivatives, and with the concomitant formation of a contact ion pair **25f**_OTf_ favoring the S_N_2 process in less ionizing solvents (Figure 5c). Recent studies conducted by Moran et al. support the ionization via a S_N_1 process for trifluoromethylcarbinol derivatives related to **22** under TfOH–HFIP activation [51].

**Scheme 7:**
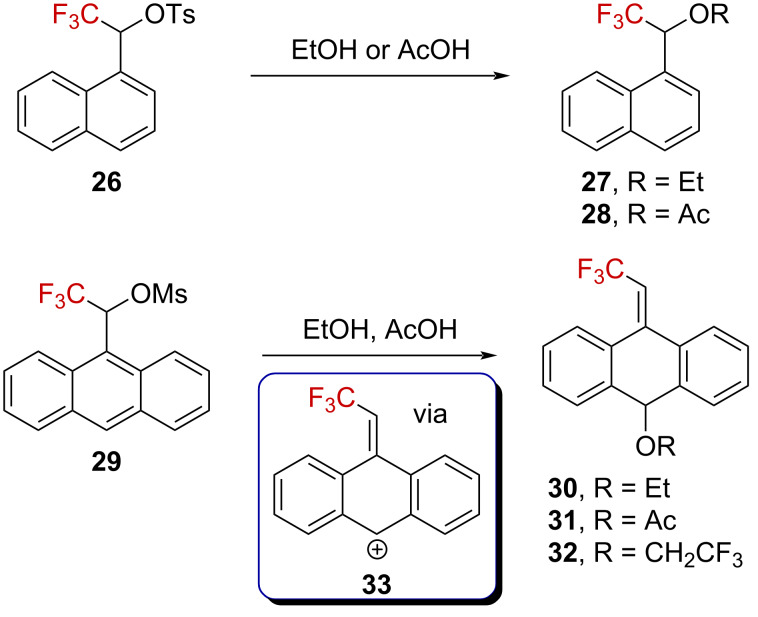
Solvolysis reaction of naphthalene and anthracenyl derivatives **26** and **29**.

Tidwell et al. investigated CF_3_-containing naphthyl- and anthracenylsulfonate derivatives **26** and **29** [52]. They reported that while the solvolysis of **26** afforded the expected compounds **27** and **28**, that of **29** exclusively gave the ring-substituted products **30**–**32** (Scheme 7). A Grunwald–Winstein plot gave linear dependences of the solvolysis rate against *Y*_OTs_ in both cases, suggesting that the formation of the carbenium ions was the rate-limiting step. Thus, the formation of products **30**–**32** is best explained by a complete charge delocalization from an α-(trifluoromethyl)carbenium ion to anthracenylcarbenium ion **33**, with subsequent trapping of **33** by the solvent.

**Figure 6:**
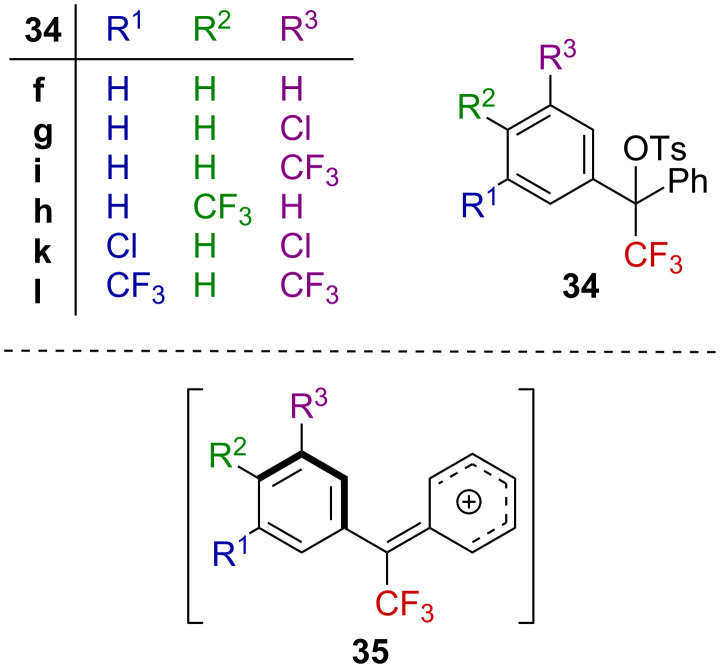
Structure of bisarylated derivatives **34**.

The solvolysis of the bisarylated α-CF_3_-substituted tosylates bearing electron-withdrawing substituents was investigated by Liu and Kuo [53]. The Hammett–Brown correlation considering derivatives **34** (Figure 6) gave a linear free-energy correlation with ρ^+^ = −3.98, which is approximately half the value of those previously reported for the benzylic α-CF_3_-substituted tosylate derivatives **13** substituted by a methyl group (Figure 2) [43,48]. The presence of the additional phenyl group, in addition to the CF_3_ group, was suggested to induce a lower ρ^+^ value. This could be explained in terms of a twisted electron-poor aryl ring, which was not in the plane of the carbenium ion for stereoelectronic reasons. The cation is thus stabilized by the additional phenyl ring in **35** (Figure 6).

**Figure 7:**
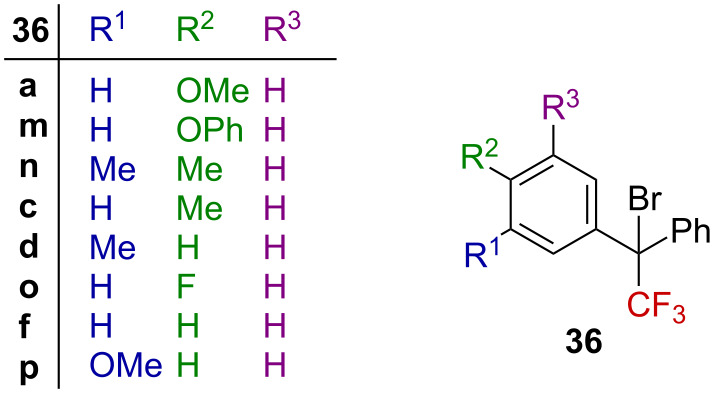
Structure of bisarylated derivatives **36**.

As an extension of the previous study, Liu et al. explored the solvolysis of tertiary, highly congested benzylic α-CF_3_-substituted halides **36** (Figure 7) [54]. Similar to their previous results, they obtained straight lines upon plotting the Hammett–Brown or Yukawa–Tsuno correlations, with ρ^+^ values from −5.9 to −7.4, depending on the solvent and on the chosen treatment. These values are close to those obtained from previous studies, suggesting a significant stabilization of the transient carbenium ion by the ring.

**Scheme 8:**
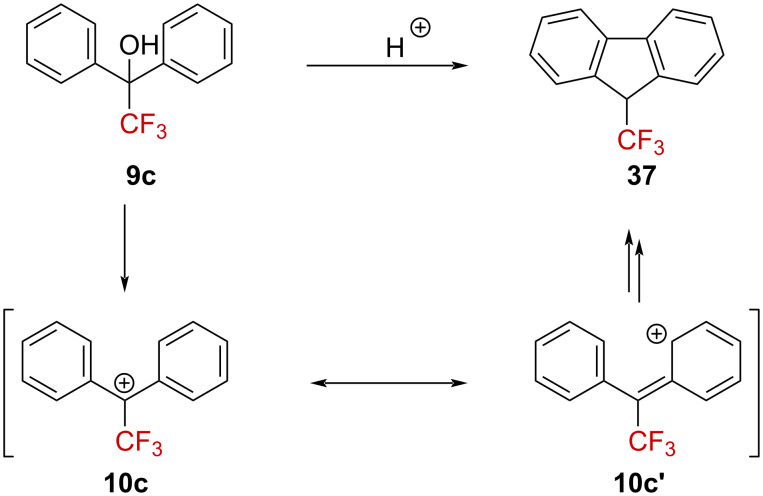
Reactivity of **9c** in the presence of a Brønsted acid.

Early interest in bisarylated α-CF_3_-substituted alcohols was shown by Cohen and Kaluszyner [55–56] and by Streitwieser et al. [57]. The cyclodehydration of **9c** occurs in polyphosphoric acid to afford fluorene **37** (Scheme 8) [57]. A mechanistic proposal invoking the initial generation of the α-(trifluoromethyl)carbenium ion **10c**↔**10c’** was mentioned by the authors [55–56]. Related studies on diphenyl derivative **9c** in a mixture of H_2_SO_4_ and chloroform also showed the formation of fluorene derivative **37** in 25% yield [58].

**Scheme 9:**
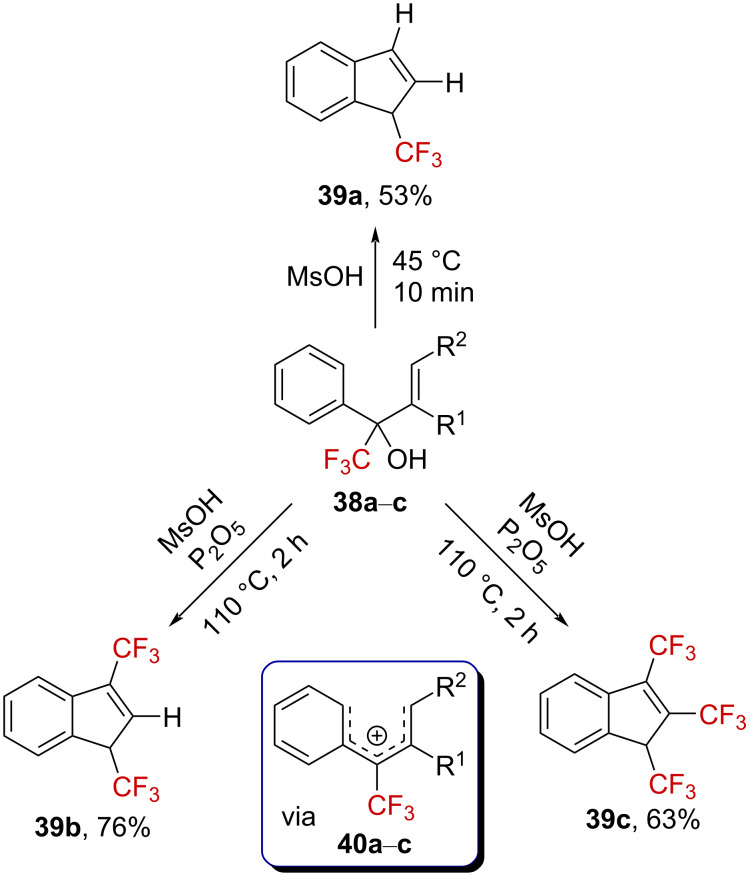
Cationic electrocyclization of **38a**–**c** under strongly acidic conditions.

Exploiting this impact of the trifluoromethyl substituent in the cationic Nazarov electrocyclization, the synthesis of CF_3_-substituted indenes **39a**–**c** from the α-(trifluoromethyl)allyl-substituted benzyl alcohols **38a**–**c** in strong acids has been reported (Scheme 9) [59]. The significant rate retardation observed upon the addition of further CF_3_ groups, illustrated by the need for harsh reaction conditions, strongly supports the formation of delocalized α-(trifluoromethyl)carbenium ions **40a**–**c**.

**Scheme 10:**
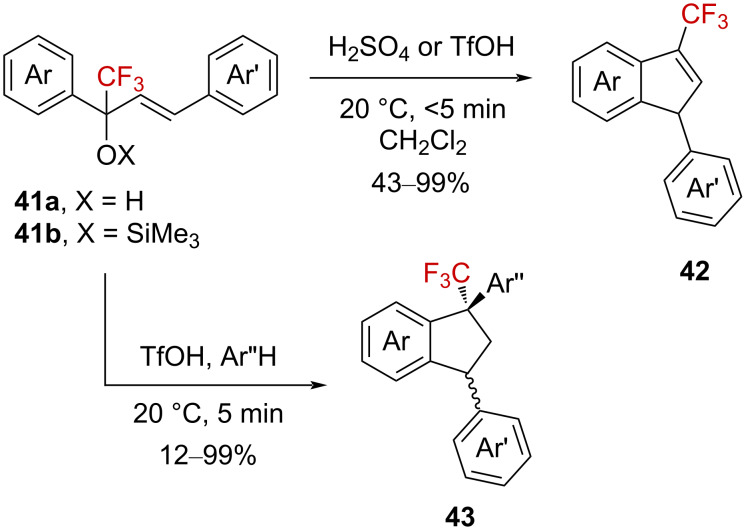
Brønsted acid-catalyzed synthesis of indenes **42** and indanes **43**.

Vasilyev et al. also investigated this Nazarov electrocyclization for the synthesis of indene derivatives. Thus, a variety of indenes **42** could be readily obtained from α-(trifluoromethyl)allyl-substituted benzyl alcohols **41a** or the corresponding silyl ethers **41b** upon the reaction in a dichloromethane solution of sulfuric acid or triflic acid [60–61]. The authors also reported that indenes **42** could undergo a subsequent Friedel–Crafts alkylation when **41b** was reacted in the presence of an external aromatic partner Ar’H in pure triflic acid. Thus, a variety of α-(trifluoromethyl) silyl ethers **41b** was converted into the corresponding indanes **43** in low to high yields [62]. The *trans*-isomers were generally obtained as the major product (Scheme 10).

**Scheme 11:**
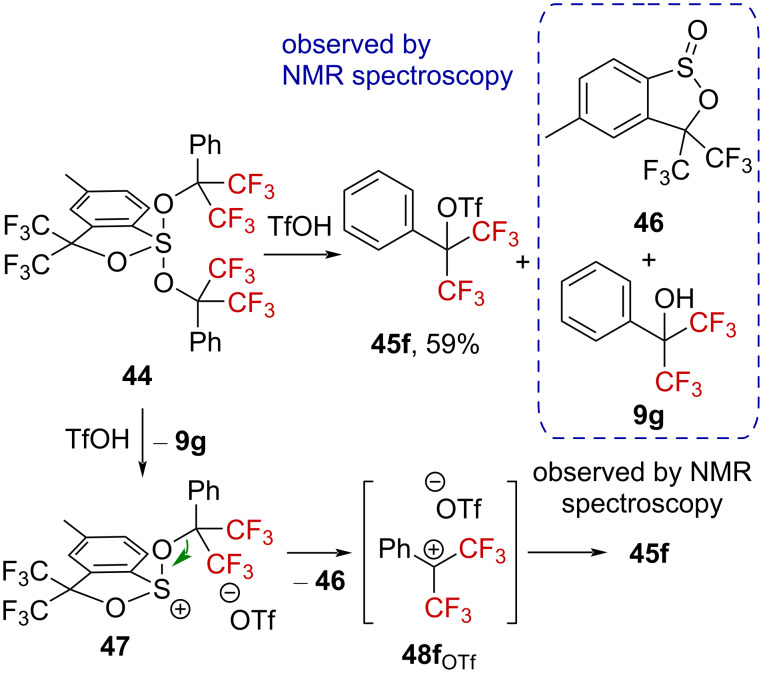
Reactivity of sulfurane **44** in triflic acid.

**Bis[α-(trifluoromethyl)]-substituted carbenium ions**: More destabilized bis(trifluoromethyl)-substituted carbenium ions have also been suggested to exist as reaction intermediates. During their investigations on the reactivity of sulfuranes under acidic conditions, Martin et al. reported that sulfurane **44** reacts with triflic acid to provide alcohol **9g** and sultine **46**, according to ^1^H and ^19^F NMR assignments, and triflate **45f**, which was isolated after basic workup of the reaction (59% yield) [63]. Hence, protonation of **44** led to dialkoxysulfonium triflate **47** along with the release of alcohol **9g**. The subsequent formation of the excellent sultine leaving group **46** (assumed to be as good of a leaving group as N_2_) [63] is the driving force for the decomposition of **47**, generating collaterally bis(trifluoromethyl)-substituted carbenium ion intermediate **48f**_OTf_. Finally, triflate **45f** is formed after ion pair recombination (Scheme 11). Similar experiments conducted with ^18^O-labeled **44** confirmed the proposed mechanism, including the transient formation of **48f_OTf_**.

**Scheme 12:**
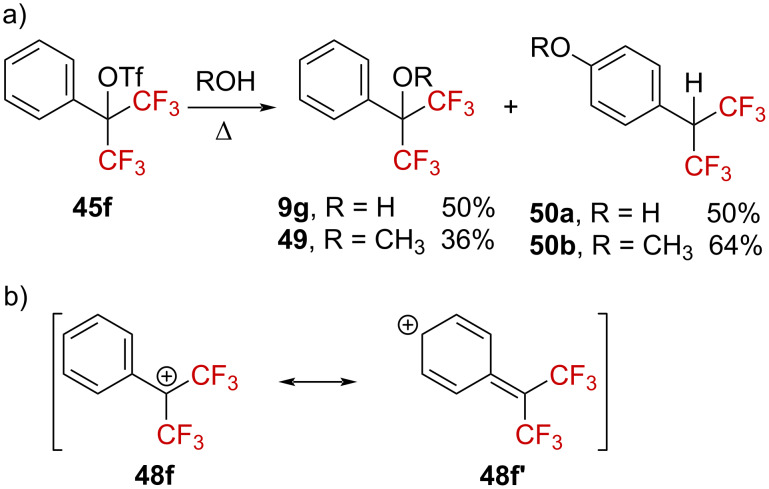
Solvolysis of triflate **45f** in alcoholic solvents.

The solvolysis of triflate **45f** was explored next [63]. Heating **45f** in water or methanol resulted in the expected solvolyzed products **9g** or **49** and the concomitant formation of **50a** or **50b** (Scheme 12a). A S_N_1 mechanism was thus suggested, with formation of the benzylic cation intermediate **48f**↔**48f’**, stabilized by the phenyl group (Scheme 12b).

**Scheme 13:**
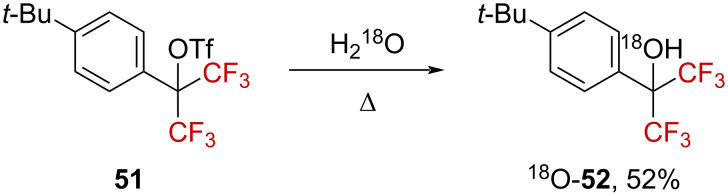
Synthesis of labeled ^18^O-**52**.

Substrate **51**, bearing a *tert*-butyl group in the *para*-position, was also submitted to solvolysis in labeled H_2_^18^O, generating the labeled benzylic alcohol ^18^O-**52** (Scheme 13). The solvolysis of **51** was found to be much faster than that of **45f** by at least a factor of 10, encouraging the authors to suggest “a transition state resembling **48f** in the rate-limiting step”.

**Scheme 14:**
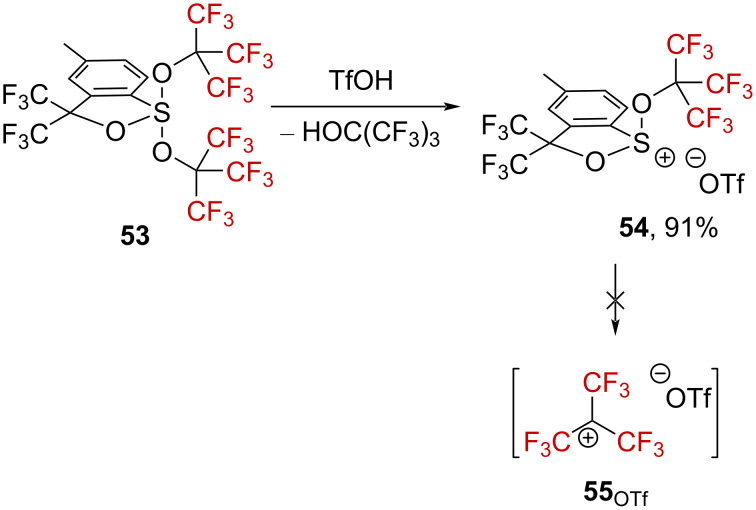
Reactivity of sulfurane **53** in triflic acid.

Sulfurane **53**, bearing OC(CF_3_)_3_ groups, was also treated with triflic acid, affording dialkylsulfonium species **54** in 91% yield along with perfluoro-*tert*-butyl alcohol (Scheme 14) [63]. No further decomposition was observed in this case, suggesting that the especially challenging perfluoro-*tert*-butylcarbenium ion **55** cannot be generated.

**Figure 8:**
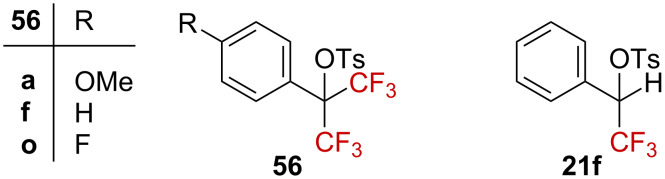
Structure of tosylates **56** and **21f**.

Highly deactivated bis(trifluoromethyl)-substituted carbenium ions and their precursors were also explored in detail by Tidwell et al. [64–66] and Richard et al. [67] in solvolysis studies of di(trifluoromethyl)-substituted tosylates **56** in comparison to the monosubstituted analogue **21f** (Figure 8). A linear free-energy relationship was found upon plotting the solvolysis rate against *Y*_OTs_ and ρ^+^ = −10.7 (TFA) for the Hammett–Brown correlation. The linear dependence of the rate on the solvent ionizing power, in addition to the strong effect of the substituents on the reactivity, are in agreement with the conclusions of Martin et al. [63] as they strongly support the formation of a bis(trifluoromethyl)-substituted carbenium ion **48**.

**Scheme 15:**
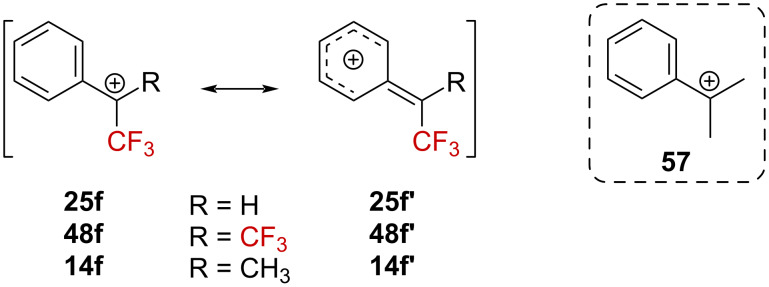
Resonance forms in benzylic carbenium ions.

Surprisingly, a relatively low kinetic effect (*k*_H_/*k*_CF3_ = 54, in TFA) was observed by comparing the solvolysis rate of tosylates **21f** and **56f**. For *p*-OMe derivatives **21a** and **56a**, *k*_H_/*k*_CF3_ = 2.5 (HFIP) was obtained. These ratios are very small compared to typical *k*_H_/*k*_CF3_ ratios in the 10^4^–10^7^ range [39–41,43,48,68]. Thus, while introducing one CF_3_ group dramatically alters the reactivity, an additional CF_3_ group does not seem to significantly impact the reactivity any further. The hypothesis of a ground-state strain release to explain this behavior was discarded as an analysis of the structures of **56f**, **13f**, and **21f** by X-ray diffraction crystallography revealed similar bond angle distortions [64–65]. A considerable delocalization of the positive charge in the aryl ring was therefore suggested (Scheme 15): in the dominant resonance form **25f’**, **48f’**, or **14f’**, the α-substituent (i.e., H, CH_3_, or CF_3_) would have a poor impact. Gas phase calculations by Tsuno et al. provided evidence for the significantly increased resonance stabilization contribution in **14f**↔**14f’** (*r* = 1.4) relative to the *t*-cumyl cation **57** (r = 1.0) [69].

### α-(Trifluoromethyl)heteroarylcarbenium ions

**Figure 9:**
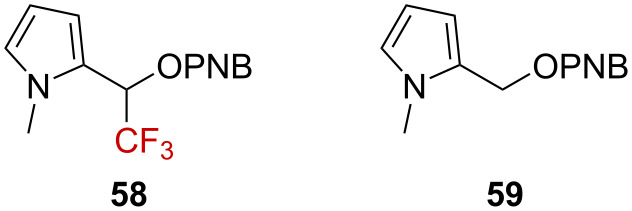
Structure of pyrrole derivatives **58** and **59**.

The presence of a strong electron-donating substituent could compensate the extreme deactivating power of the CF_3_ group, favoring a further exploitation for synthetic purposes. In this context, Tidwell and Kwong-Chip compared the solvolysis of *N*-methylpyrrole **58** to **59** (Figure 9) [70].

**Scheme 16:**
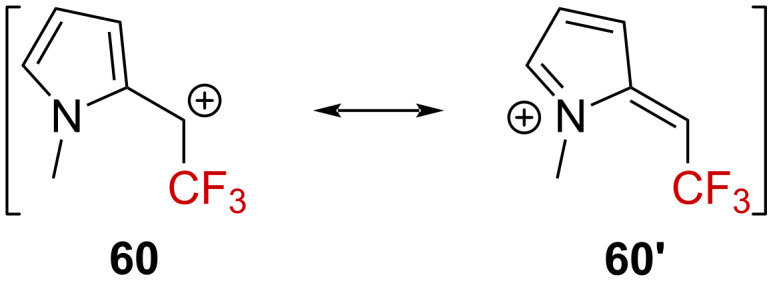
Resonance structure **60**↔**60’**.

A very similar rate was determined for **58** and **59**, with *k*_CF3_ = 4.40 × 10^−4^ s^−1^ and *k*_H_ = 1.84 × 10^−2^ s^−1^, respectively, providing a rate ratio of *k*_H_/*k*_CF3_ = 41.8. Plotting the solvolysis rate of **58** against *Y*_OTs_ led to a linear free-energy relationship supporting the rate-limiting formation of a carbenium ion **60**. The small *k*_H_/*k*_CF3_ ratio suggests here that the positive charge is highly delocalized in the pyrrole ring and should be regarded as a pyrrolium ion **60’** rather than an α-(trifluoromethyl)carbenium ion **60** (Scheme 16).

**Scheme 17:**
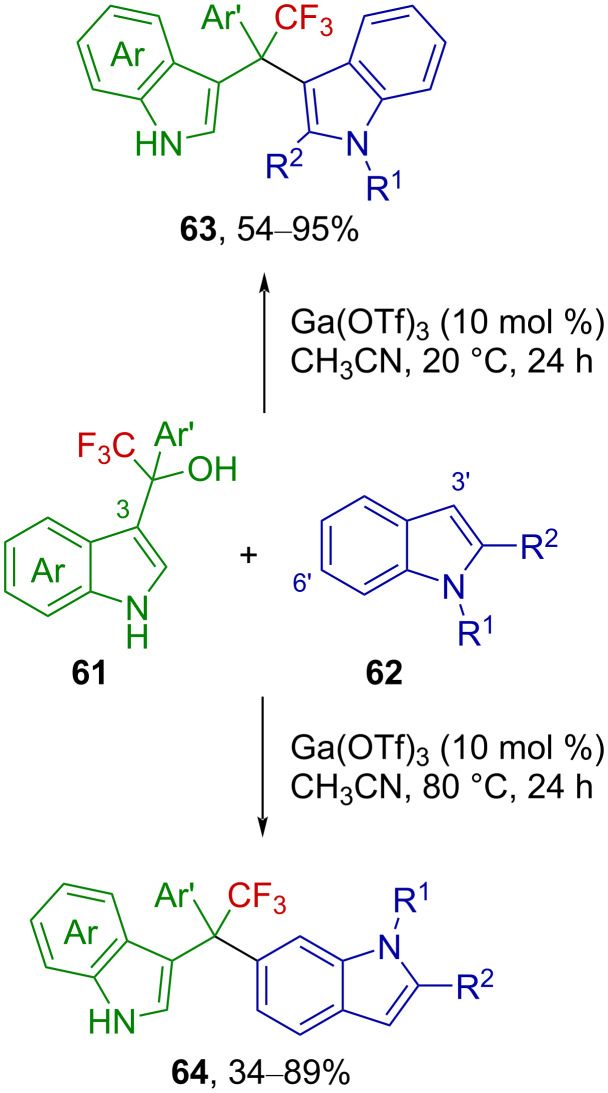
Ga(OTf)_3_-catalyzed synthesis of 3,3’- and 3,6’-bis(indolyl)methane from trifluoromethylated 3-indolylmethanols.

Similarly, trifluoromethyl-substituted indolium ions were invoked as intermediates in the recently reported gallium-catalyzed synthesis of unsymmetrical CF_3_-substituted 3,3’- and 3,6’-bis(indolyl)methanes from trifluoromethylated 3-indolylmethanols [71]. Alcohol **61** reacts with indole **62** to provide a product **63** or **64**, depending on the temperature (Scheme 17).

**Scheme 18:**
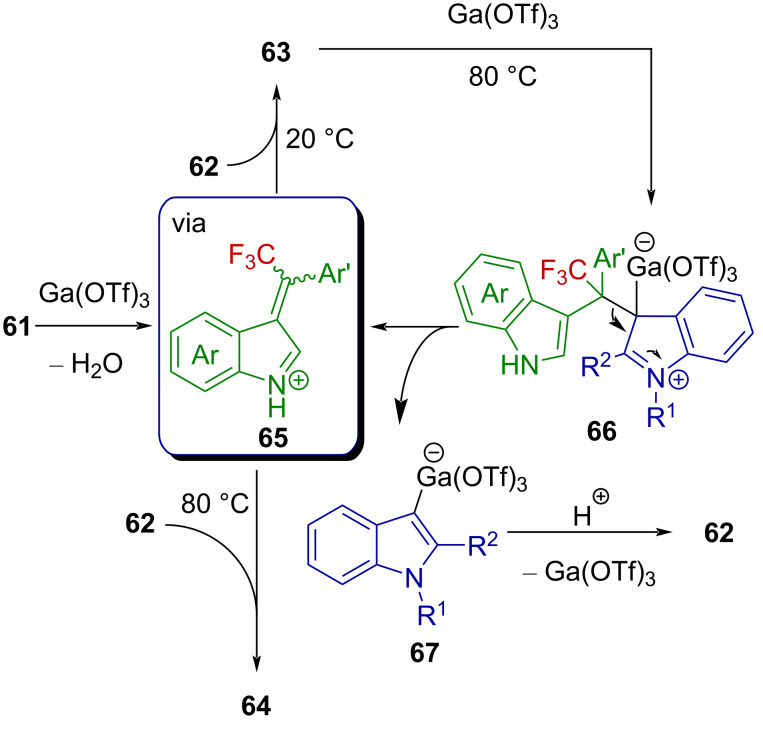
Proposed reaction mechanism.

The authors suggested that an indolium ion **65** is produced from the activation of **61** with Ga(OTf)_3_ and reacts with **62** in a Friedel–Crafts reaction to afford **63** (Scheme 18). Further control experiments showed that derivatives **63** were not stable at 80 °C under the reaction conditions and isomerized to furnish **64**. Based on these observations, the authors proposed that upon heating, Ga(OTf)_3_ reacts with **63** to release an indolium ion **65** and forms an organogallium species **67** via intermediate **66**, which, after protodemetallation, releases indole **62** and regenerates the catalyst. The retro-Friedel–Crafts reaction at 80 °C at the indole C3-position thus allows the progressive conversion of the starting material into the C6-derivative **64** (Scheme 18).

**Scheme 19:**
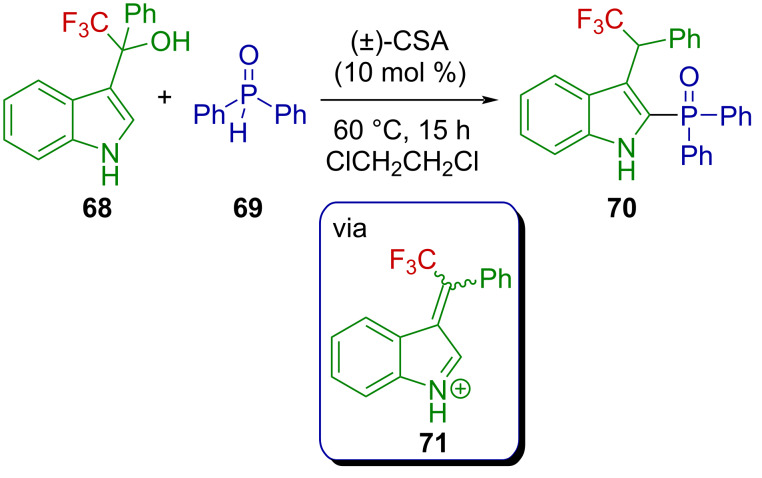
Metal-free 1,2-phosphorylation of 3-indolylmethanols.

Chen et al. reported the synthesis of C2-phosphorylated indoles via 1,2-phosphorylation of 3-indolylmethanols with *H*-phosphine oxides or *H*-phosphonates under Brønsted acid activation [72]. The scope of the reaction includes one example of a CF_3_-substituted 3-indolylmethanol, **68**, which is efficiently phosphorylated by **69** in the presence of a catalytic amount of camphor sulfonic acid (CSA) at 60 °C, affording **70**. The authors suggested the transient formation of an analogous indolium ion **71** (Scheme 19).

**Scheme 20:**
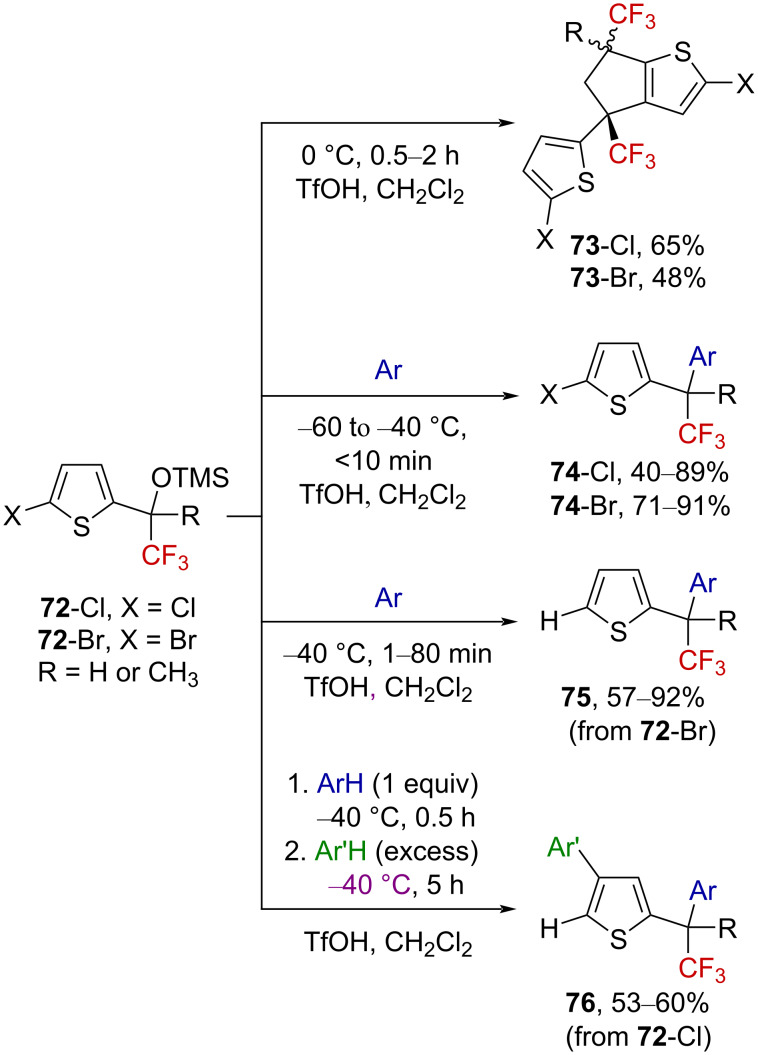
Superacid-mediated arylation of thiophene derivatives.

Very recently, Vasilyev and Khoroshilova investigated the superacid-promoted activation of α-(trifluoromethyl) silyl ethers exhibiting a thiophene core [73]. At 0 °C, thiophenes **72**-Cl and **72**-Br undergo electrophilic dimerization, affording a mixture of **73**-Cl and **73**-Br (Scheme 20). When the reaction was cooled to −60 °C < *T* < −40 °C in the presence of aromatic nucleophiles, thiophenes **72**-Cl and **72**-Br could be converted into **74**-Cl and **74**-Br derivatives via a side-chain arylation reaction. When the reaction was conducted at −40 °C, the reactivity was shown to be governed by the nature of the halogen atom. For the brominated derivatives **72**-Br, the corresponding side-chain arylation reaction occurred at −60 °C, but a further hydrodehalogenation led to the bromine-free derivatives **75**. For the chlorinated derivatives **72**-Cl, a similar side-chain arylation−hydrodehalogenation sequence occurred, but an additional Friedel–Crafts arylation at the C4-position led to derivatives **76**. In this latter case, a two-step one-pot process was developed in order to access derivatives bearing two different aromatic rings.

**Scheme 21:**
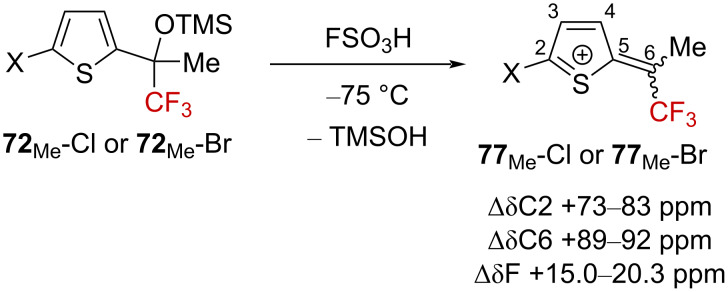
In situ mechanistic NMR investigations.

Mechanistic investigations were then undertaken by in situ low-temperature NMR experiments, allowing the observation of thiophenium ions **77**_Me_-Cl and **77**_Me_-Br (Scheme 21). ^19^F NMR analysis showed significant downfield shifts for the signal of the CF_3_ group compared to the neutral precursors, characteristic of α-(trifluoromethyl)carbenium ions. However, and as expected, the ^13^C NMR spectra showed considerable downfield shifts for the carbon atoms C2 and C6, suggesting a highly delocalized positive charge in the heteroaromatic ring as depicted below.

### α-(Trifluoromethyl)allylcarbenium ions

**Scheme 22:**
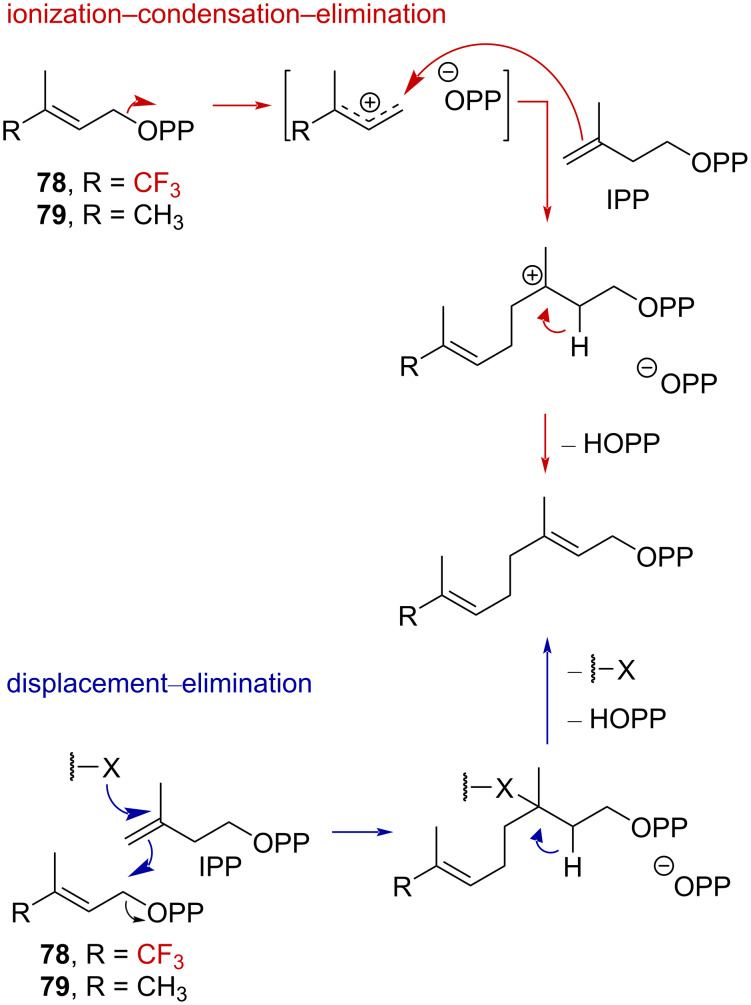
Proposed mechanisms for the prenyltransferase-catalyzed condensation.

In 1976, Poulter et al. exploited the powerful electron-withdrawing effect of the CF_3_ group to elucidate the prenyltransferase-catalyzed condensation mechanism [74–75]. The authors envisioned that substituting a methyl group in isopentenyl pyrophosphate (IPP) by a CF_3_ group (Scheme 22, **79**→**78**) should greatly reduce the reaction rate in the case of an ionization–condensation–elimination mechanism, while a small acceleration should be observed in the case of a displacement–elimination mechanism.

**Scheme 23:**
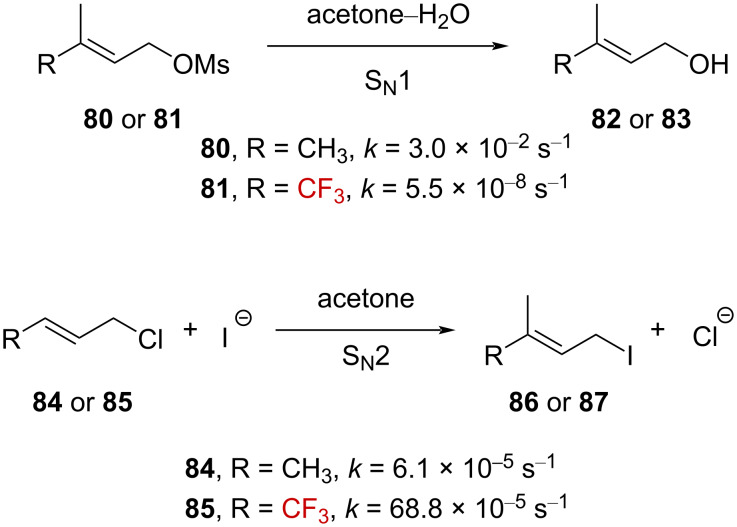
Influence of a CF_3_ group on the allylic S_N_1- and S_N_2-mechanism-based reactions.

Promising results were first obtained during investigations conducted on CF_3_-substituted derivatives in S_N_1- and S_N_2-mechanism-based reactions (Scheme 23). A profound retardation effect for the solvolysis of **81** in acetone–H_2_O (S_N_1) with *k*_CH3_/*k*_CF3_ = 5.4 × 10^5^ was observed, while **85** promoted the Finkelstein reaction (S_N_2) about 11 times faster than **84** (*k*_CH3_/*k*_CF3_ = 8.9 × 10^−2^, Scheme 23). This is the result of a destabilized cationic intermediate in the first case and a stabilized negatively charged transition state in the second.

**Scheme 24:**
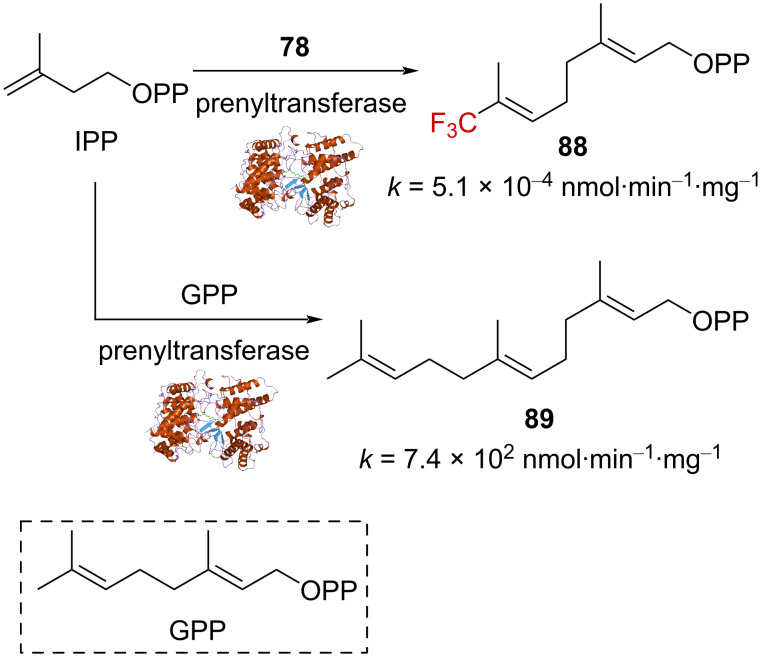
Influence of the CF_3_ group on the condensation reaction.

When **78** was incubated in the presence of IPP and the enzyme prenyltransferase, a rate of 5.1 × 10^−4^ nmol⋅min^−1^⋅mg^−1^ was measured for the condensation reaction (Scheme 24), which is to be compared to a value of 7.4 × 10^2^ nmol⋅min^−1^⋅mg^−1^ observed for the condensation involving IPP and geranyl pyrophosphate (GPP). **78** was 1.5 × 10^6^ times less reactive than geranyl pyrophosphate, allowing to conclude that the condensation mechanism involving prenyltransferase as a catalyst occurs via an ionization–condensation–elimination sequence.

**Scheme 25:**
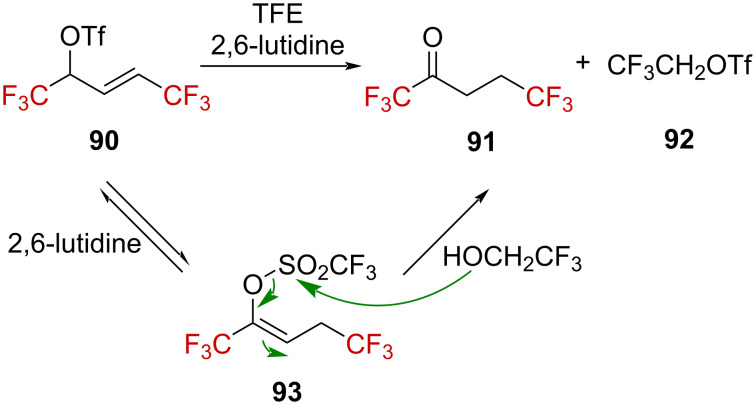
Solvolysis of **90** in TFE.

As suggested by the aforementioned studies, α-(trifluoromethyl)-substituted allylic carbenium ions could exist in solution. The solvolysis of CF_3_-substituted allyl sulfonates was thus thoroughly examined by Gassmann and Harrington [76]. The solvolysis of doubly CF_3_-deactivated **90** in trifluoroethanol (TFE) required the presence of 2,6-lutidine, leading to ketone **91** and triflate **92**. This observation suggests that lutidine allows the isomerization of **90** into **93**, followed by a nucleophilic attack of the solvent at the sulfur atom (Scheme 25).

**Scheme 26:**
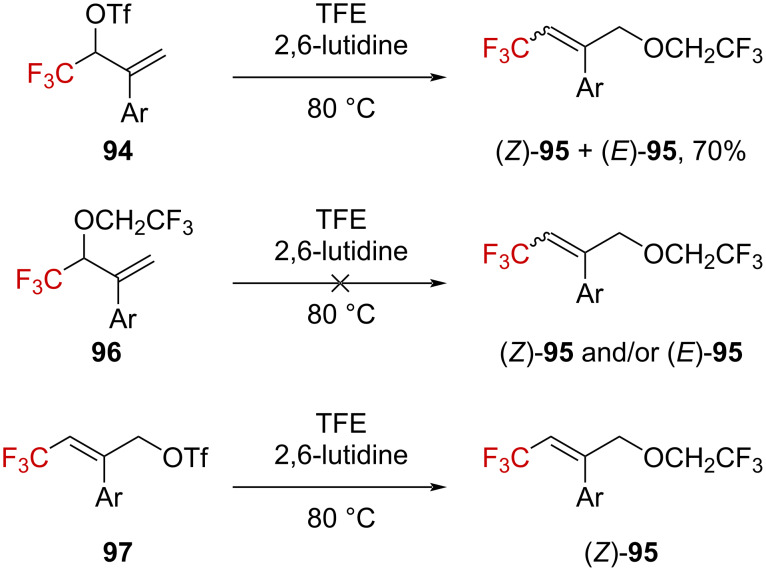
Solvolysis of allyl triflates **94** and **97** and isomerization attempt of **96**.

The reactivity of analogous monotrifluoromethyl-substituted allyl derivatives **94**, bearing an aryl group in the vinylic position was also explored (Scheme 26). Trifluoroethanolysis of secondary triflate **94** gave a mixture of (*Z*)-**95** and (*E*)-**95** in a combined 70% yield, with an *E*/*Z* ratio of 17:83–8:92, depending on the nature of the aryl substituent (*p*-OMe or *p*-Cl, respectively). It is worth noting that the formation of S_N_2 product **96** was not observed. Similar observations have been reported by Langlois et al. [77]. In order to get some insights into the mechanism, derivative **96** was synthesized and subjected to solvolysis. However, this compound was found to be stable under the reaction conditions [52]. When primary triflate **97** was subjected to solvolysis, the expected product (*Z*)-**95** was obtained, and the rate was 50–100 times faster than when starting from **94**. The Hammett–Brown correlation gave a poor dependence of the rate on the nature of the aryl substituent, and thus suggesting that the aryl group does not participate in the positive-charge stabilization. Finally, the Grunwald–Winstein plot gave a linear free-energy relationship between the rate and *Y*_OTs_, supporting the formation of a carbenium ion.

**Scheme 27:**
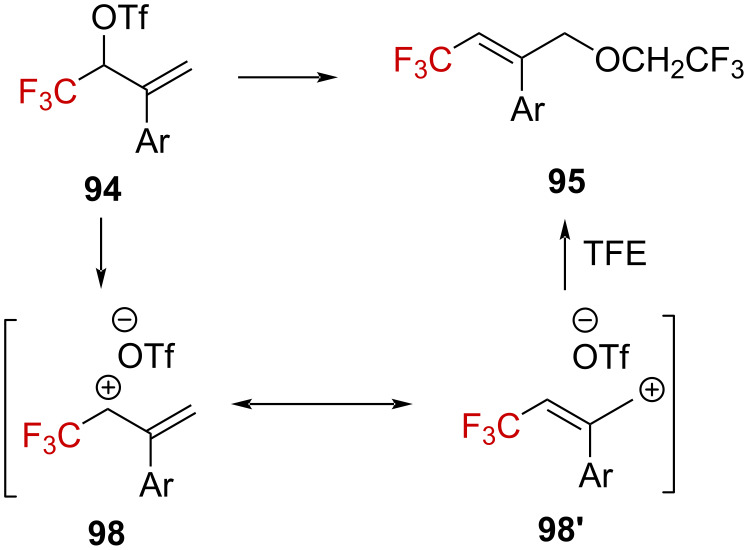
Proposed mechanism for the formation of **95**.

From these observations, the authors concluded that **94** dissociates into an ion pair **98** in the rate-limiting step, in which the delocalized positive charge is highly concentrated in the γ-CF_3_ position (see **98’**), which is the electronically and sterically privileged position for the solvent approach, to subsequently give **95** (Scheme 27).

**Scheme 28:**
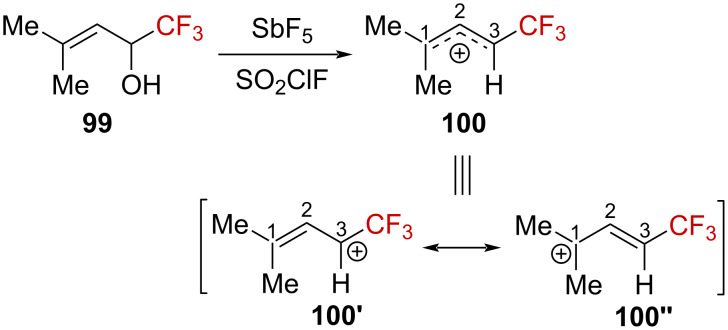
Formation of α-(trifluoromethyl)allylcarbenium ion **100** in a superacid.

Prakash et al. also investigated the formation of α-(trifluoromethyl)allylcarbenium ions from alcohol precursors in a superacid [78]. When allylic alcohol **99** was ionized with SbF_5_ in SO_2_ClF at −78 °C, the corresponding α-(trifluoromethyl)allylcarbenium ion **100** was formed. The carbons atoms C1 and C2 exhibited very different chemical shifts, δ_C1_ = 157 ppm and δ_C2_ = 290 ppm, which are to be compared to the nontrifluoromethylated analogue (δ_C1_ = 206 ppm and δ_C2_ = 251.8 ppm). The authors suggested that “the positive charge is more unevenly localized in the cation” **100**, with the resonance form **100’’** contributing significantly more than **100’** (Scheme 28). This unsymmetrical delocalized structure in carbenium compound **100** was also confirmed by DFT calculations at the B3LYP/6-31G* level, with a C2–C3 bond considerably shorter than the C1–C2 bond, with d_C2_–_C3_ = 1.359 Å and d_C1_–_C2_ = 1.427 Å.

**Scheme 29:**
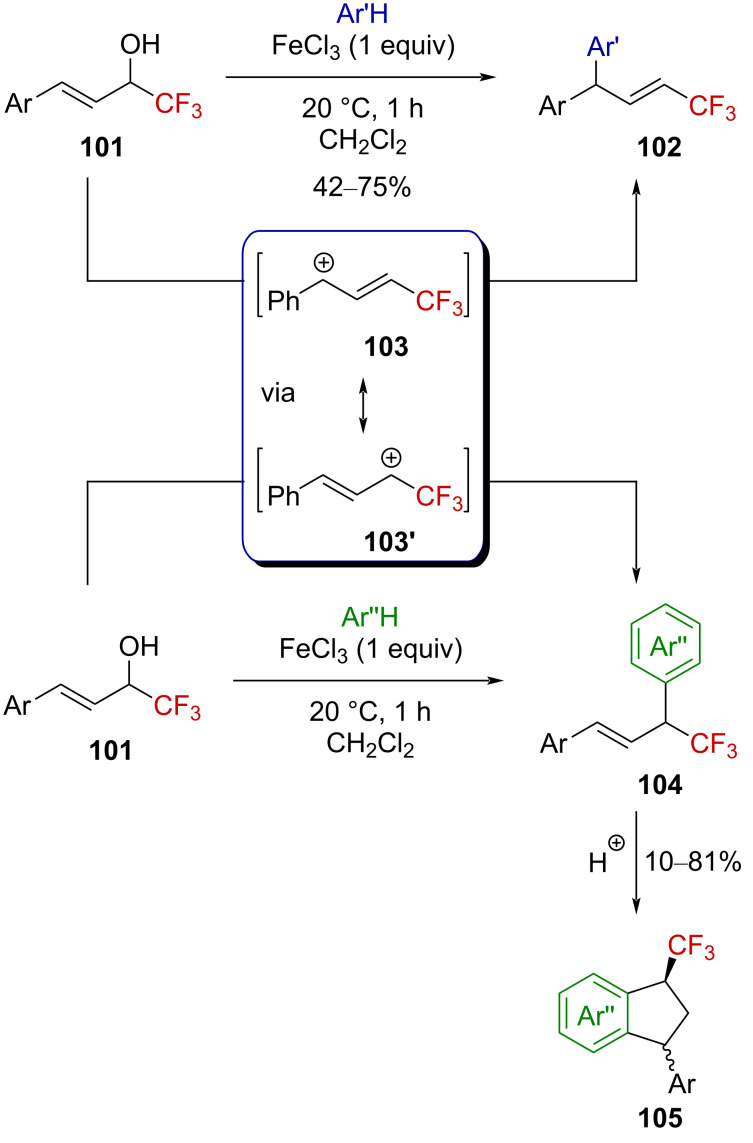
Lewis acid activation of CF_3_-substituted allylic alcohols.

More recently, Vasilyev et al. reported that Lewis acid activation of α-(trifluoromethyl)allyl alcohol **101** allowed the transient formation of the corresponding α-(trifluoromethyl)allylcarbenium ion **103**↔**103’**, the resonance form **103** of which could be trapped with arenes to afford (trifluoromethyl)vinyl-substituted derivatives **102** (Scheme 29) [79–80]. It was also suggested that the resonance form **103’** has a nonnegligible contribution as this α-(trifluoromethyl)allylcarbenium ion could be trapped by some electron rich arenes (i.e., xylene, cumene, etc.). The products **104** further react to afford indanes **105** after hydroarylation. A closely related study on dibrominated allylic α-(trifluoromethyl) alcohols also invoked the transient formation of allylic carbenium ions, such as **103** [81].

### α-(Trifluoromethyl)alkynylcarbenium ions

**Scheme 30:**
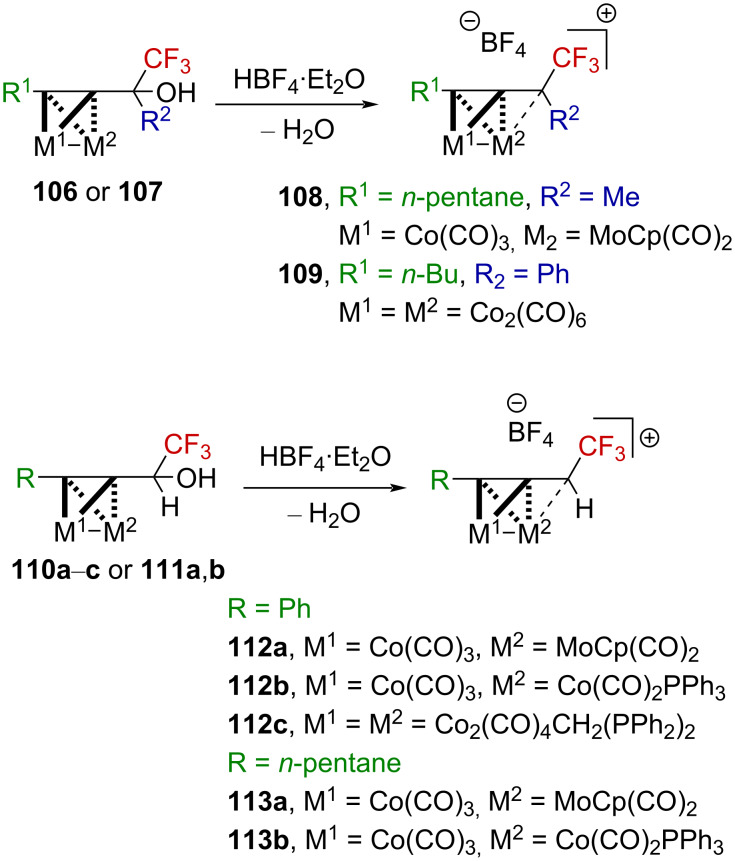
Bimetallic-cluster-stabilized α-(trifluoromethyl)carbenium ions.

It has been reported that the complex of Co_2_(CO)_6_ and propargyl alcohols allows the facile generation of the corresponding propargylium ions (Nicholas reaction) in a relatively strong acidic medium (i.e., TFA, BF_3_⋅Et_2_O, etc.). These cobalt-cluster-stabilized propargylium ions exhibit a surprisingly high thermodynamic stability, comparable to that of triarylmethylcarbenium ions and are readily observable by NMR spectroscopy or isolable as salts with relatively weakly coordinating anions (BF_4_^−^, PF_6_^−^, etc.) [82]. In this context, Gruselle et al. exploited the strong stabilization provided by Co–Co and Co–Mo bimetallic clusters to generate α-(trifluoromethyl)propargylium ions (Scheme 30). While the tertiary carbenium ion **108** was isolable as a solid [83–84], the tertiary carbenium ion **109** and the secondary derivatives **112a**–**c** and **113a**,**b** afforded oils. The secondary derivatives were much more sensitive in spite of the use of electron-rich Co–Mo clusters and could only be characterized by NMR and IR spectroscopy [85]. Upon ionization, the change in the electronic density is directly reflected by the downfield shift of the ^19^F NMR chemical shift of the CF_3_ group but also by a CO shift to a higher frequency. As a general example, **111a** (δ_19F_ = −75.9 ppm; ν_CO_ = 2051, 2001, 1984, and 1942 cm^−1^) affords **113a** (δ_19F_ = −59.2 ppm; ν_CO_ = 2104, 2065, 2055, 2006, and 1989 cm^−1^), which exhibits the previously mentioned features, with Δδ_19F_ = +16.7 ppm and Δν_CO_ ≈ +50 cm^−1^.

**Scheme 31:**
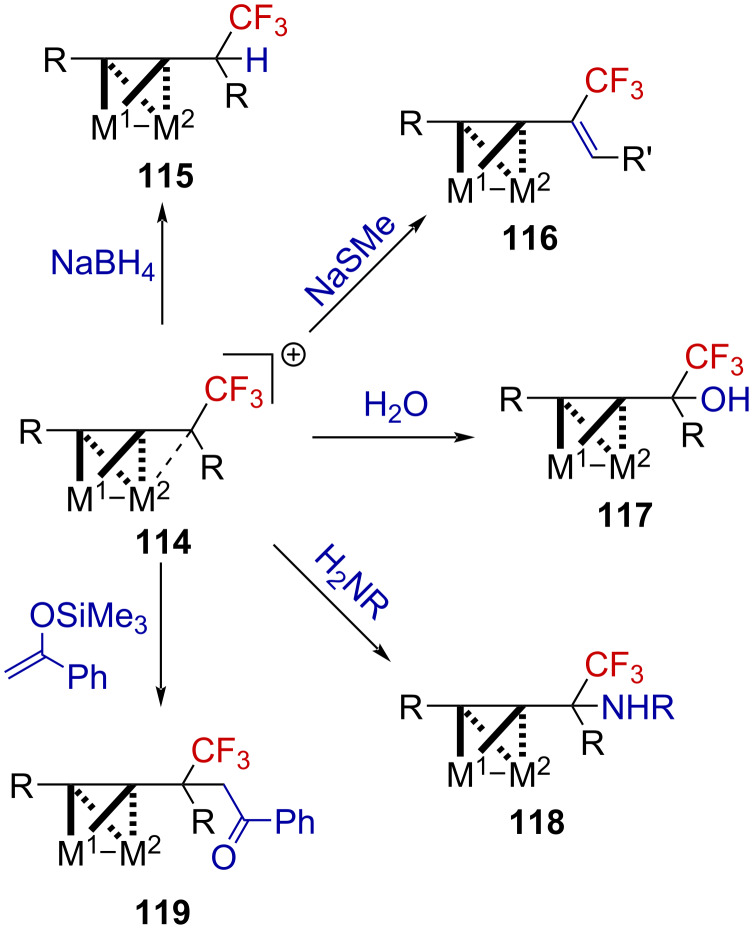
Reactivity of cluster-stabilized α-(trifluoromethyl)carbenium ions.

Beyond the synthetic challenges associated with the generation of such species, the authors explored their use in organic synthesis. These metal-stabilized α-(trifluoromethyl)propargylium ions **114** could be engaged in useful transformations, such as reductions, eliminations, as well as C–O, C–N, or C–C bond formations (Scheme 31).

**Scheme 32:**
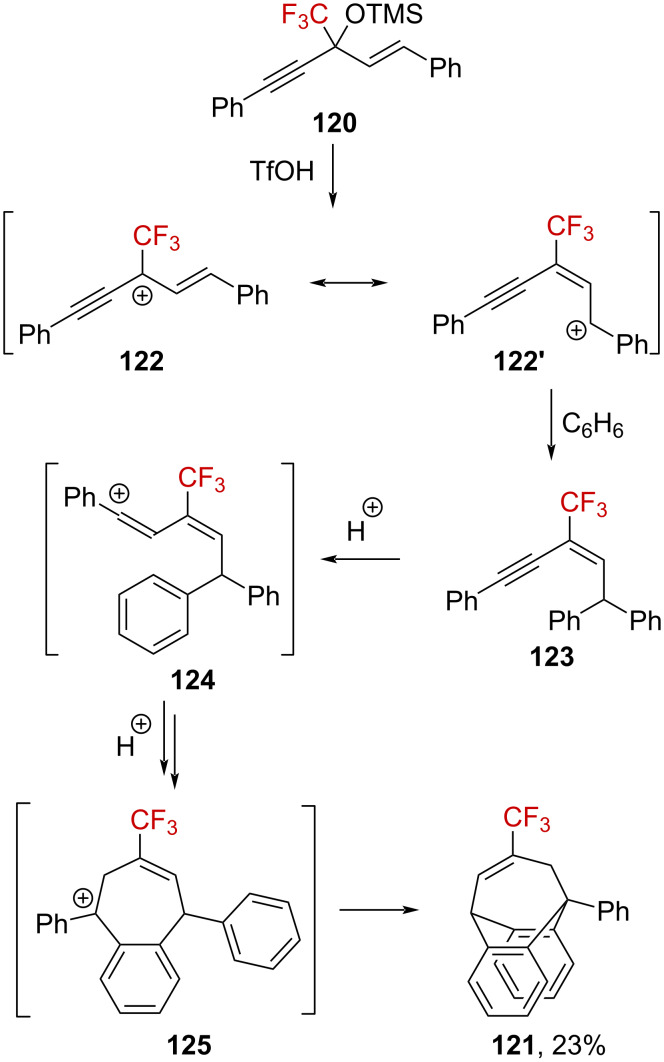
α-(Trifluoromethyl)propargylium ion **122**↔**122’** generated from silyl ether **120** in a superacid.

α-(Trifluoromethyl)propargylium has also been suggested as an intermediate in superacid-mediated Friedel–Crafts reactions [86]. When [α-(trifluoromethyl)propargyl]allyl silyl ether **120** was added to a dichloromethane solution of triflic acid in the presence of benzene, the original [3.2.2]-bridged CF_3_-substituted product **121** was obtained. The authors proposed an elimination of TMSOH to generate the propargyl-substituted α-(trifluoromethyl)allylcarbenium ion **122** at first, which is a resonance form of the benzylic carbenium ion **122’**. Subsequently, **122’** reacts in a Friedel–Crafts reaction with benzene to generate **123**. After two successive hydroarylation reactions, the final product **121** is produced via the formation of vinylic and benzylic carbenium ions **124** and **125**, respectively (Scheme 32).

**Scheme 33:**
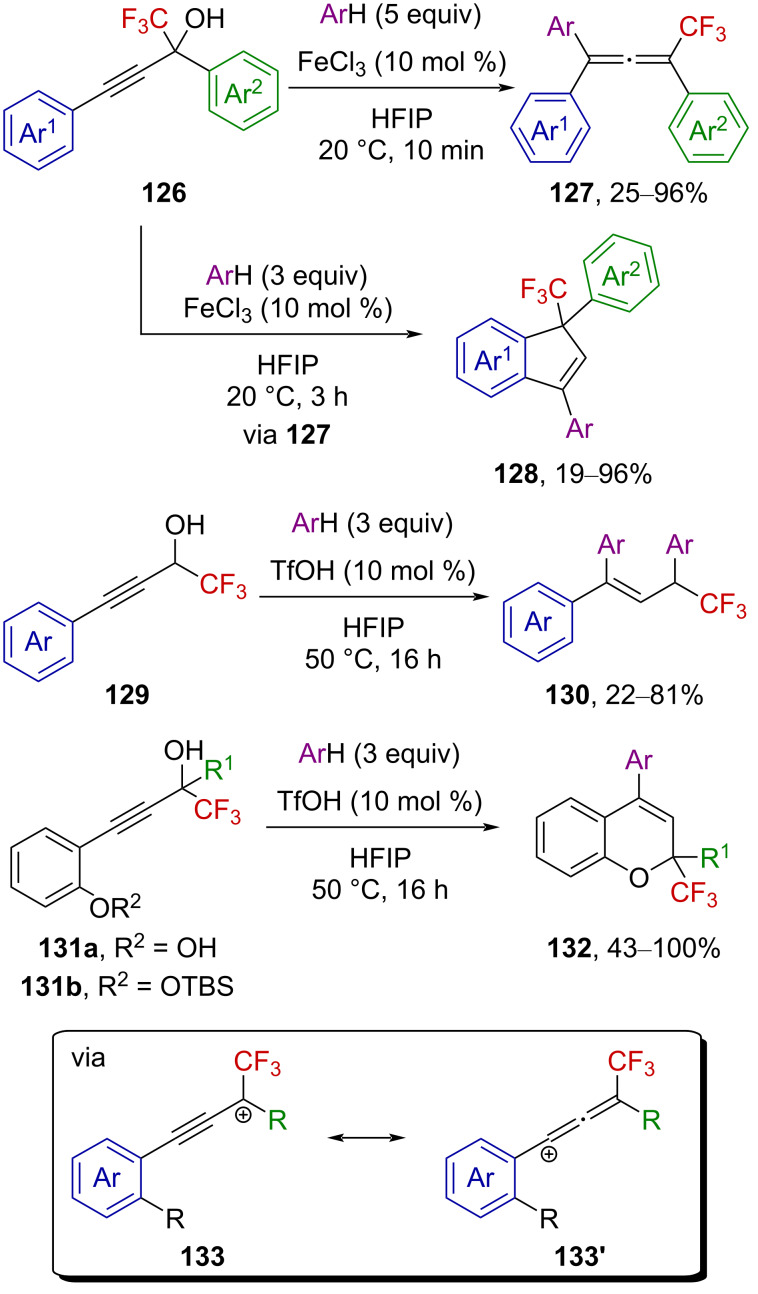
Formation of α-(trifluoromethyl)propargylium ions from CF_3_-substituted propargyl alcohols.

Moran et al. also investigated the reactivity of a variety of CF_3_-substituted propargyl alcohols (Scheme 33) [87]. The reactivity of the benzylic (trifluoromethyl)propargyl alcohol **126** strongly depends on the reaction conditions, as allenes **127** or indenes **128** were both obtained under FeCl_3_ activation. Indeed, with a longer reaction time, allenes **127** undergo a subsequent intramolecular hydroarylation reaction leading to indenes **128**. The authors suggested the formation of FeCl_3_–HFIP complexes being involved in a Lewis acid-assisted Brønsted acid catalysis. The CF_3_-substituted propargyl alcohol **129** was found to undergo tandem Friedel–Crafts hydroarylation reactions to give derivatives **130** under TfOH activation at 50 °C. Finally, CF_3_-substituted chromene derivatives **132** were obtained under the same reaction conditions from *ortho*-hydroxy or *ortho*-silyloxy derivatives **131a** and **131b**, respectively. The common intermediate in these reactions is supposed to be α-(trifluoromethyl)propargylium ion **133**↔**133’**.

### Heteroatom-substituted α-(trifluoromethyl)carbenium ions

**Scheme 34:**
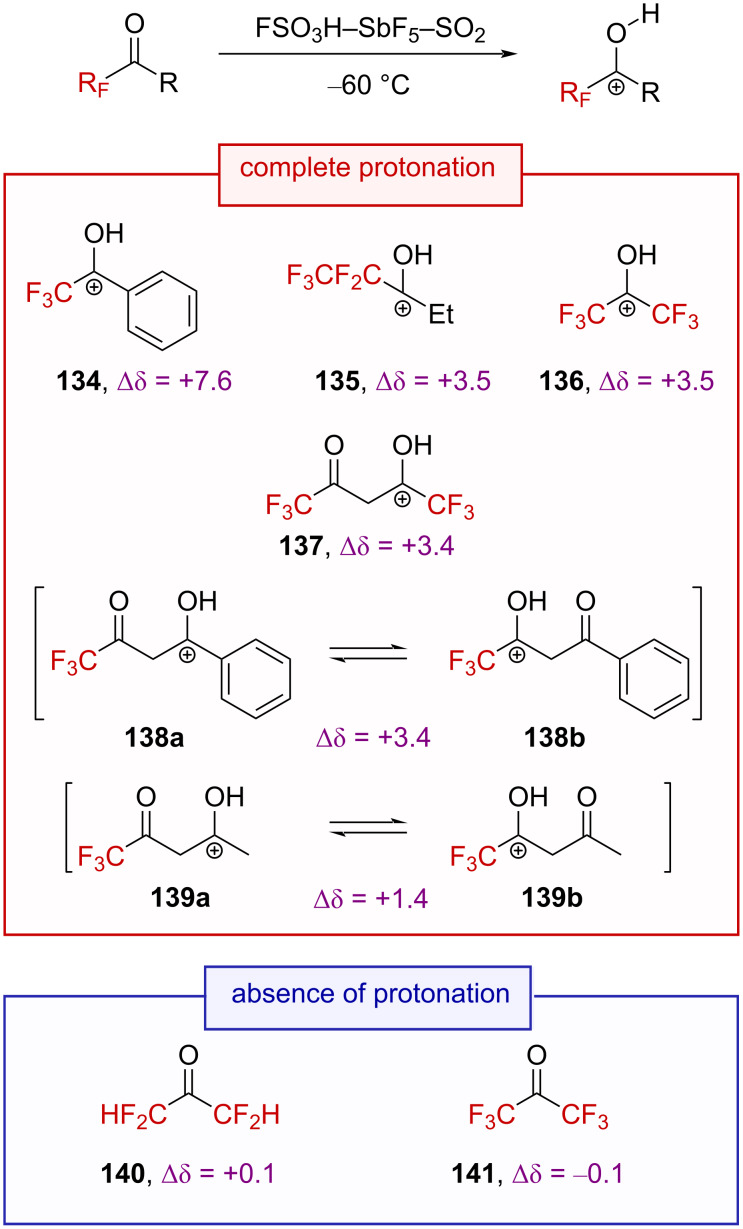
Direct NMR observation of the protonation of some trifluoromethyl ketones in situ and the corresponding ^19^F NMR chemical shifts. Δδ = δ_19F,product_ − δ_19F,precursor_ (δ in ppm).

The stabilization of carbenium ions through oxygen lone pair back-donation [35] is a common feature in organic synthesis [88–90]. In this context, Olah, Pittman, et al. investigated the protonation of a variety of trifluoromethyl ketones in a superacid [35,91]. Trifluoromethyl ketone protonation was observed by NMR spectroscopy at −60 °C in a superacidic FSO_3_H–SbF_5_–SO_2_ solution (Scheme 34).

**Scheme 35:**
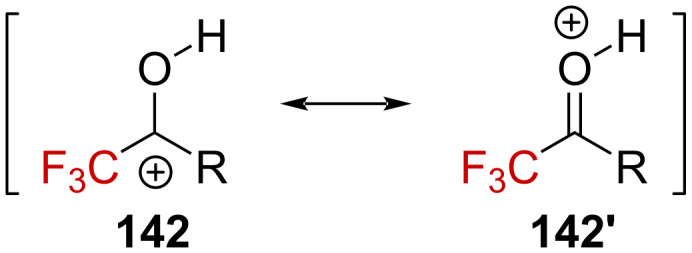
Selected resonance forms in protonated fluoroketone derivatives.

The ^19^F chemical shift variation for the generated oxygen-substituted trifluoromethylated carbenium ions ranged from +7.6 to +1.4 ppm, significantly lower than for carbon-substituted α-(trifluoromethyl)carbenium ions (e.g., the carbenium ion **10a**, Δδ = +24.8 ppm), confirming the considerable contribution of the oxygen lone pair to the stabilization of the cation **142**↔**142’** (Scheme 35).

**Scheme 36:**
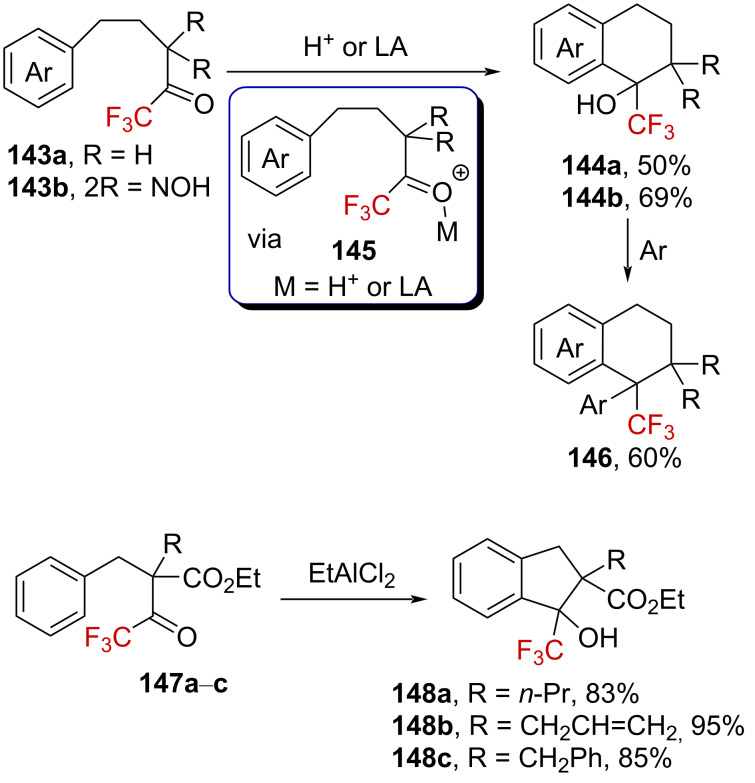
Acid-catalyzed Friedel–Crafts reactions of trifluoromethyl ketones **143a**,**b** and **147a**–**c**.

Oxygen-stabilized α-(trifluoromethyl)carbenium ions (oxocarbenium ions) have been exploited for chemical synthesis [92–94]. Ketone **143a** and ketoxime **143b** undergo Friedel–Crafts reactions in the presence of Brønsted or Lewis acid to furnish the corresponding CF_3_-containing tetralin derivatives **144a** and **144b**, respectively (Scheme 36). In addition, **144a** could be further converted into **146** in the presence of aromatic nucleophiles (e.g., benzene or toluene). Similarly, derivatives **147a**–**c** could also be converted into indanol derivatives **148a**–**c** in high yields (Scheme 36) [95].

**Scheme 37:**
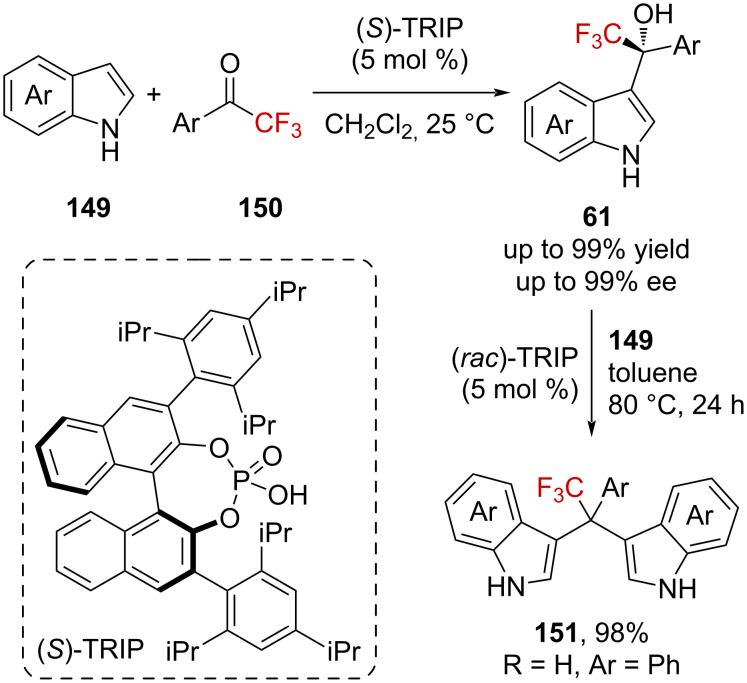
Enantioselective hydroarylation of CF_3_-substituted ketones.

Ma et al. managed the enantioselective arylation of aromatic trifluoromethyl ketones **150** with (*S*)-TRIP (Scheme 37) [96]. A variety of CF_3_-substituted enantioenriched benzylic alcohols **61** were thus synthesized after the trapping of protonated CF_3_-substituted ketones **134** (Scheme 37). Interestingly, these benzylic alcohols **61** did not undergo further arylation and were stable under the reaction conditions. In agreement with computational studies [97], this behavior was assigned to the presence of the CF_3_ group, which induces a shortening of the C–O bond in the product (*d*_C–O_ = 1.426 Å) compared to the CH_3_ analogue (*d*_C–O_ = 1.438 Å) and strongly inhibits the formation of the α-(trifluoromethyl)bisarylcarbenium ion, as illustrated by the higher activation energy needed for the dehydration (Δ*E*_CF3_ = 21.0 kcal⋅mol^−1^ vs Δ*E*_CH3_ = 14.8 kcal⋅mol^−1^ at the B3LYP/6-31+G(d,p) level). On the other hand, the first arylation reaction seems to be facilitated by the CF_3_ group (Δ*E*_CF3_ = 16.9 kcal⋅mol^−1^ vs Δ*E*_CH3_ = 21.2 kcal⋅mol^−1^ at the B3LYP/6-31+G(d,p) level). Raising the temperature finally favors the dehydration and the second Friedel–Crafts reaction to afford bisarylated products **151**.

**Scheme 38:**
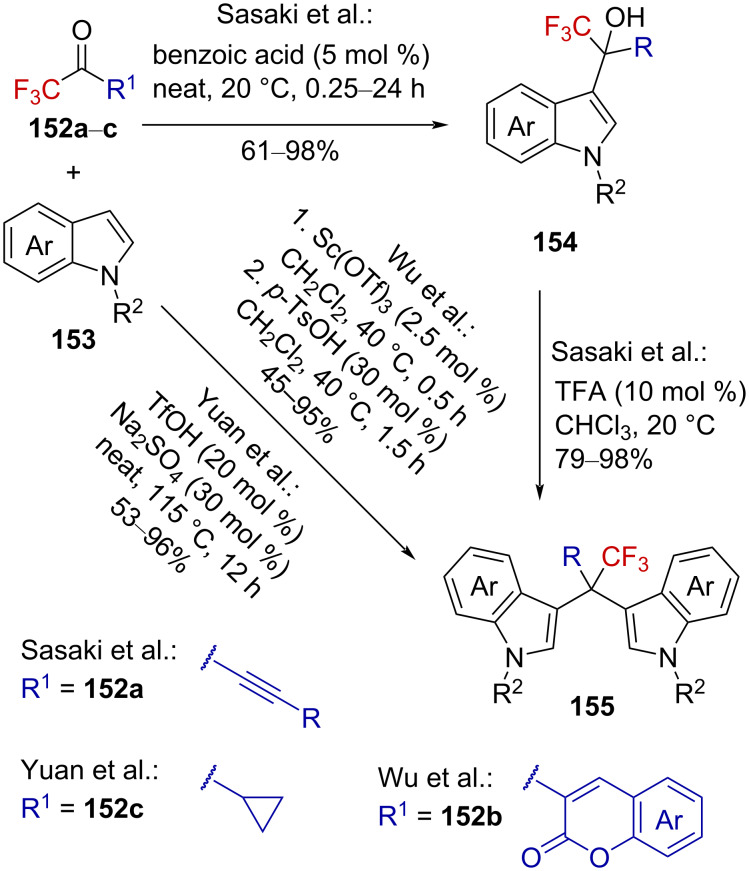
Acid-catalyzed arylation of ketones **152a**–**c**.

In complementary studies, Sasaki et al. reported the acid-catalyzed mono- and diarylation of CF_3_-substituted α,β-ynones **152a** [98], Wu et al. reported the one-pot two-step acid-catalyzed diarylation of trifluoroacetyl coumarins **152b** [99], and Yuan et al. reported the acid-catalyzed diarylation of CF_3_-substituted cyclopropyl ketone **152c** [100] (Scheme 38). In these reactions, oxygen-stabilized α-(trifluoromethyl)carbenium ions **142** are supposed to be generated by protonation or Lewis acid activation of the starting ketones.

**Scheme 39:**
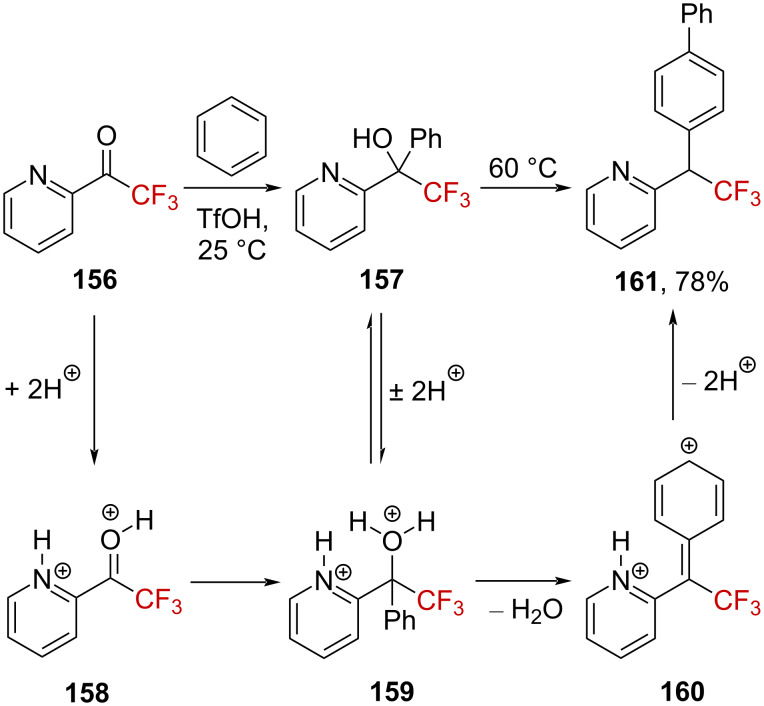
Reactivity of **156** in a superacid.

Klumpp et al. explored the reactivity of CF_3_-substituted superelectrophiles (defined as multiply charged cationic electrophiles [101]) generated in superacid media [102]. Hence, when trifluoroacetyl pyridine **156** was treated with benzene in triflic acid, alcohol derivative **157** was obtained. In a superacid, **156** generates a dication **158** in which the electrophilicity is enhanced through a strong charge repulsion (Scheme 39). This dication reacts with benzene to provide pyridinium–oxonium dication **159** in solution. Further arylation does not occur spontaneously, which was evident because alcohol **157** was isolated at the end of the reaction. Upon heating at 60 °C, the second arylation takes place, presumably via the formation of dicationic superelectrophile **160**. Again, due to charge repulsions as well as due to the strong electron-withdrawing effect of the CF_3_ group, the positive charge adjacent to the CF_3_ group is highly delocalized within the phenyl ring, and arylation occurs regioselectively at the *para*-position, affording biaryl species **161**.

**Scheme 40:**
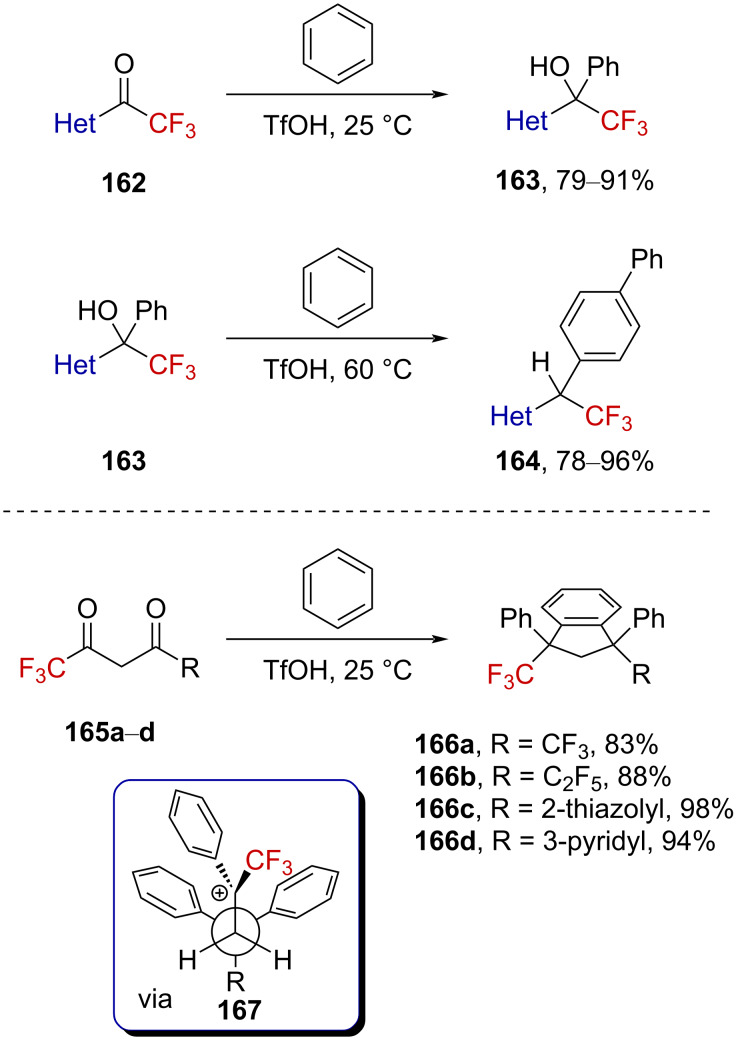
Reactivity of α-CF_3_-substituted heteroaromatic ketones and alcohols as well as 1,3-diketones.

Using this strategy, several trifluoromethyl ketones **162** and alcohols **163** bearing heteroaryl substituents (i.e., benzothiazole, quinoline, isoquinoline, benzimidazole, or imidazole) prone to be protonated were elegantly converted into the corresponding alcohols **163** and biphenyl compounds **161** in high yield (Scheme 40, top). The reaction of CF_3_-substituted 1,3-diketones **165a**–**d** in TfOH was also deeply investigated by Klumpp et al. [101]. The *syn*-indanes **166a**–**d** could cleanly be generated after successive well-defined arylation reactions via **167** (Scheme 40, bottom). Moreover, the CF_3_ group was found to be essential in this reaction as 2,4-pentanedione did not react with benzene under similar conditions.

**Scheme 41:**
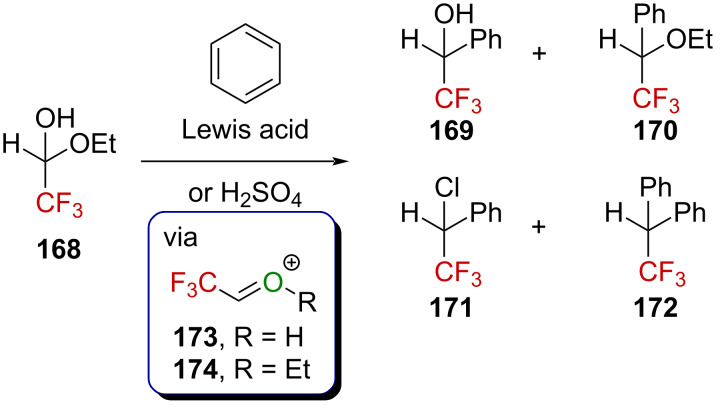
Reactivity of **168** with benzene in the presence of a Lewis or Brønsted acid.

The use of acetal derivatives in place of ketones as precursors of oxygen-stabilized α-(trifluoromethyl)carbenium ions was also investigated. For instance, the readily available hemiacetal **168** was shown to react with benzene in the presence of a Lewis acid or H_2_SO_4_ to form compounds **169**–**172** in various amounts, depending on the acid used (Scheme 41) [103]. It is assumed that an oxygen-stabilized α-(trifluoromethyl)carbenium ion is involved. It was shown that **168** could also react with (hetero)arenes [104–105] and alkenes [106] under Lewis acid activation but also with electron-rich arenes under thermal activation [107–109].

**Scheme 42:**
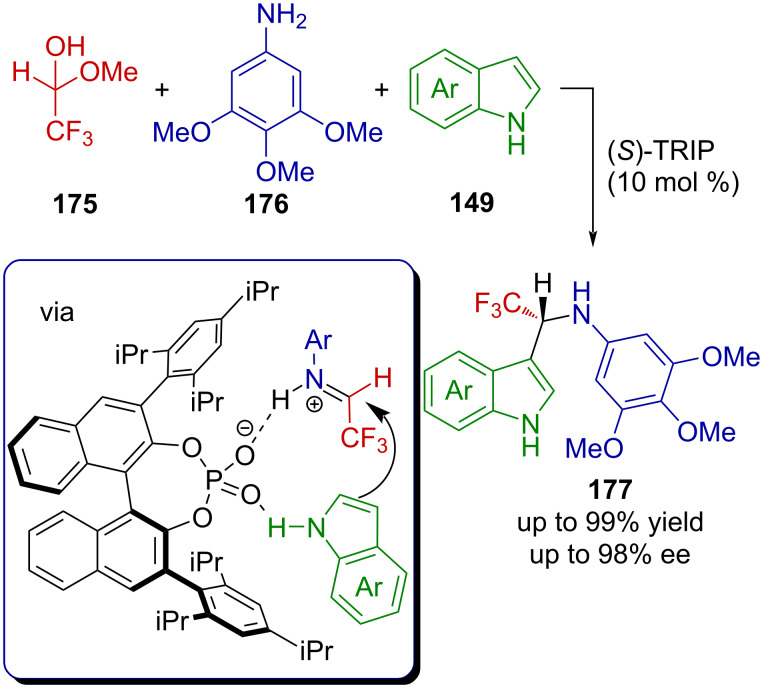
Acid-catalyzed three-component asymmetric reaction.

CF_3_-substituted hemiacetal **168** can react with amines to furnish the corresponding hemiaminal ethers, which can be further activated by a Lewis acid to generate CF_3_-substituted iminium ions able to promote Friedel–Crafts alkylations [110–111]. Ma et al. exploited this mode of activation in a Brønsted acid-catalyzed three-component asymmetric reaction [112]. Mixing hemiacetal **175**, arylaniline **176**, and indole derivatives **149** in the presence of a catalytic amount of the moderately acidic (*S*)-TRIP (p*K*_a_ = 3–4 in DMSO [113–114]) in dichloromethane afforded the chiral α-(trifluoromethyl)aminoaryl derivatives **177** in an excellent yield and enantiomeric excess (Scheme 42). The authors proposed that hemiacetal **175** and amine **176** react under the reaction conditions to give an imine in the first step, which is protonated by (*S*)-TRIP to generate the corresponding chiral CF_3_-substituted iminium ion. The latter subsequently reacts via the most accessible *Re*-face with indole **149** to afford the resulting Friedel–Crafts product **177**. Worthy of note is the fact that the reaction works equally well with a CHF_2_-containing hemiacetal.

**Scheme 43:**
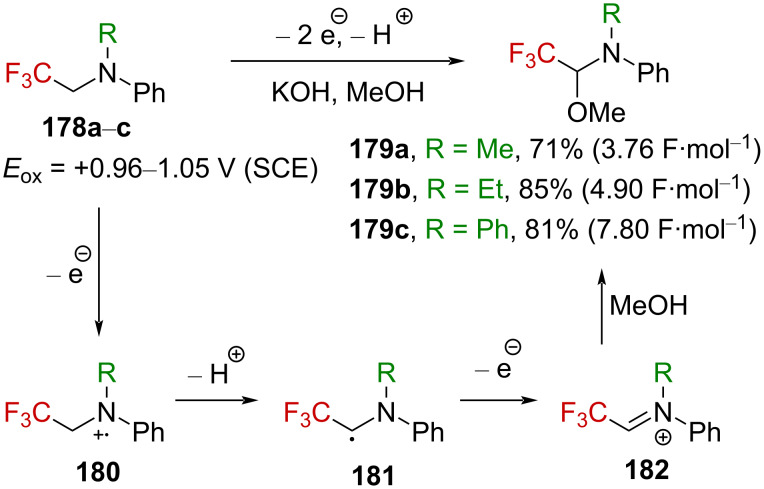
Anodic oxidation of amines **178a**–**c** and proposed mechanism.

Nitrogen-stabilized α-(trifluoromethyl)carbenium ions have also been extensively investigated. Under electrochemical conditions, trifluoromethylated iminium ions **182** were successfully generated by Fuchigami et al. [115]. Starting from tertiary amines **178a**–**c**, the corresponding hemiaminal ethers **179a**–**c** were obtained (Scheme 43). The reaction is highly regioselective as no methoxylation of **178a** and **178b** was observed on the nontrifluoromethylated alkyl substituent (Me or Et). Hence, although amines **178a**–**c** are more difficult to oxidize than their nonfluorinated analogues (*E*_ox_ (PhNMe_2_) = +0.71 V (SCE)), the radical cation **180** is formed under the reaction conditions, and deprotonation at the methylene unit near the CF_3_ group is highly favored because of the higher acidity, accounting for the observed high regioselectivity. In addition, the transient stabilization of radical **181** by the captodative effect could also favor the general process.

**Scheme 44:**
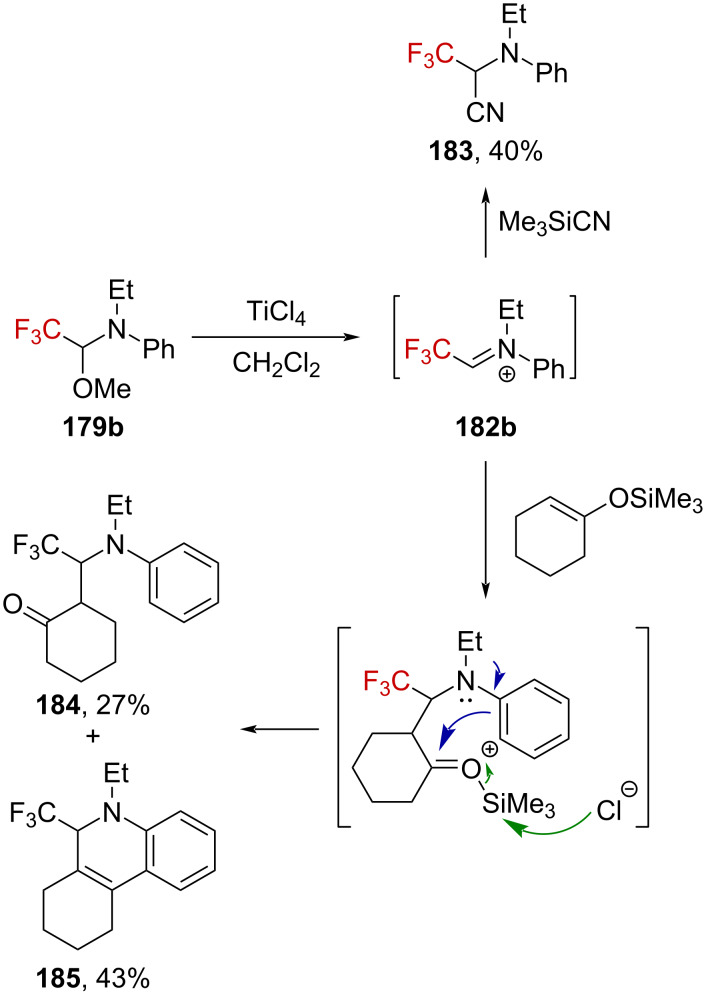
Reactivity of **179b** in the presence of a strong Lewis acid.

Lewis acid activation of trifluoromethylated hemiaminal ethers has also been studied by Fuchigami et al. [115–116]. For instance, when **179b** is treated with a slight excess of TiCl_4_ in dichloromethane, iminium ion **182b** can be trapped by TMSCN to furnish α-(trifluoromethyl)-α-aminonitrile **183** in 40% yield. The iminium was also successfully trapped by a silyl enol ether, affording a mixture of ketone **184** and heterocycle **185** (Scheme 44).

**Scheme 45:**
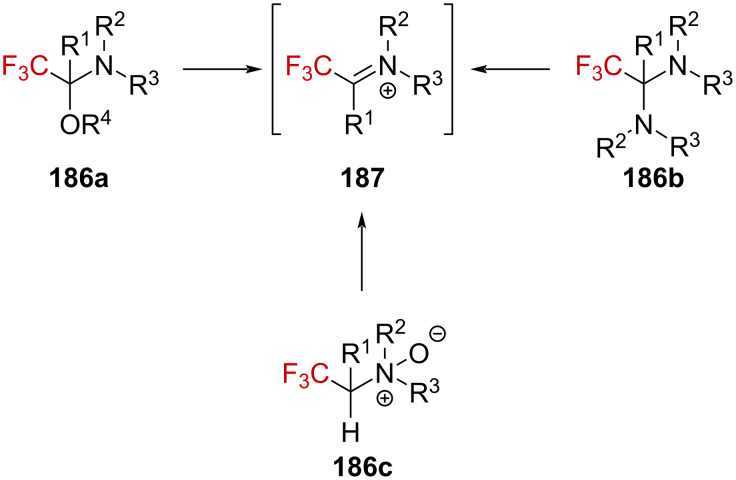
Trifluoromethylated derivatives as precursors of trifluoromethylated iminium ions.

The trifluoromethyl-substituted derivatives **186a**–**c** have then been exploited as a convenient source of trifluoromethylated iminium ions **187** (Scheme 45) [117–119].

**Scheme 46:**
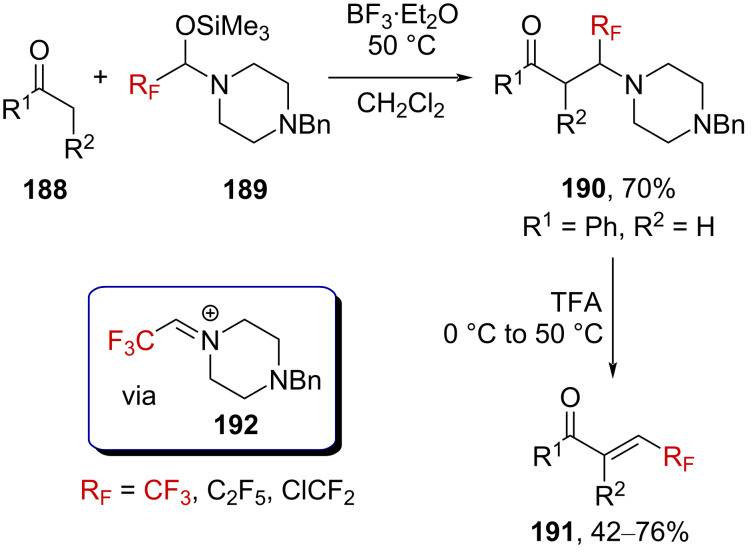
Mannich reaction with trifluoromethylated hemiaminal **189**.

Langlois, Billard, and Blond reported on the Mannich-type reaction between silylated trifluoromethylated hemiaminal derivatives **189** [120] and enolizable ketones **188** [121]. The intermediate formation of trifluoromethylated iminium ion **192** by Lewis acid activation was suggested by the authors (Scheme 46). The resulting CF_3_-substituted β-amino ketones **190** could then be efficiently transformed in a one-pot procedure into the corresponding CF_3_-substituted enones **191** upon Brønsted acid treatment.

**Scheme 47:**
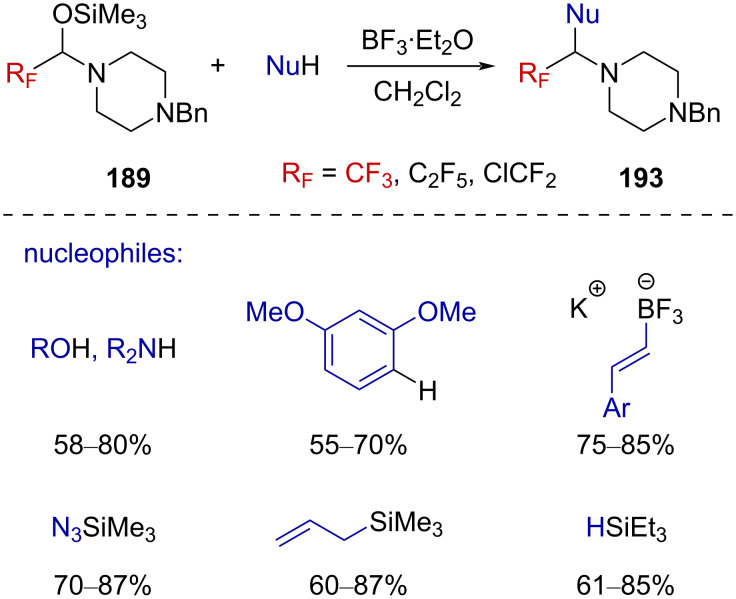
Suitable nucleophiles reacting with **192** after Lewis acid activation.

Langlois and Billard then exploited the reactivity of the trifluoromethylated iminium ion **192** and extended the scope of the reaction to a larger panel of nucleophiles, including alcohols, amines, aromatic and vinyl derivatives, as well as silylated nucleophiles (Scheme 47) [122].

**Scheme 48:**
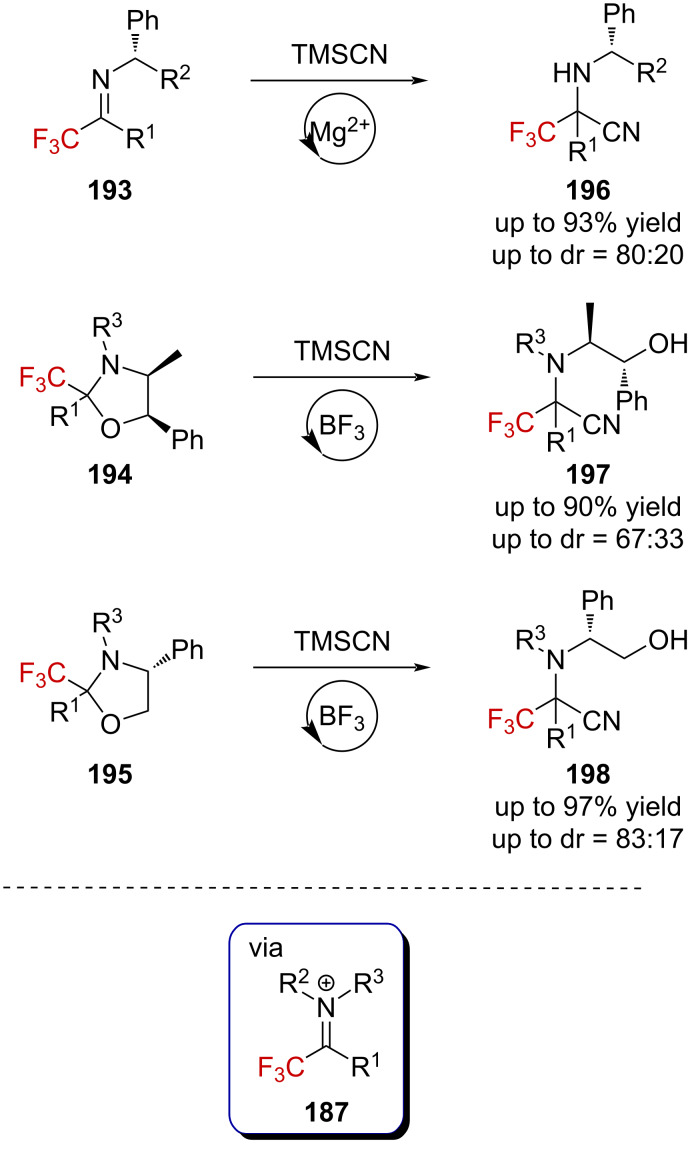
Strecker reaction involving the trifluoromethylated iminium ion **187**.

Brigaud and Huguenot also suggested the formation of a trifluoromethylated iminium ion **187** during the course of their studies on a Strecker-type reaction [123]. Starting from trifluoromethylated imines **193** or oxazolidines **194** and **195** bearing enantiopure chiral auxiliaries, the authors accessed the corresponding cyano derivatives **196**–**198** with different levels of diastereoselectivity (Scheme 48). Further development by Brigaud et al. allowed the synthesis of CF_3_-substituted pseudoprolines structurally related to oxazolidines **194** and **195** [124].

**Scheme 49:**
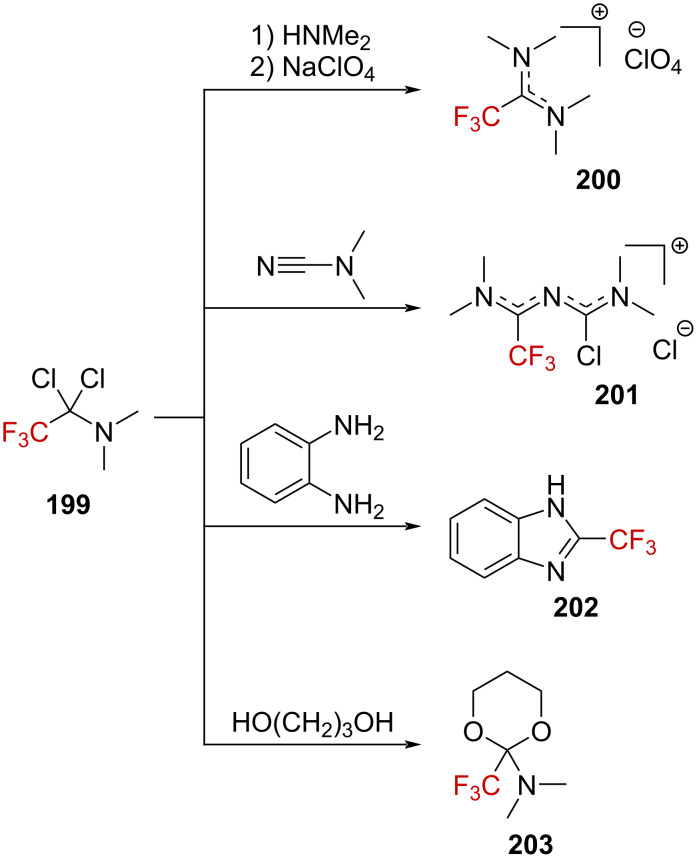
Reactivity of **199** toward nucleophiles.

Viehe et al. also contributed by developing the chloroalkylamino reagent **199**, bearing a geminal CF_3_ group, which proved to be a valuable synthon for the introduction of the CF_3_ group into molecules [125]. Thus, **199** exhibits a high reactivity towards many functionalities, as depicted below (Scheme 49). Interestingly, **200** and **201** are sufficiently stable to be synthesized, presumably due to electron delocalization (guanidinium ions).

Following these seminal contributions, the chemistry of CF_3_-substituted iminium ions **187** was extensively exploited for synthetic purposes [126–138].

**Scheme 50:**
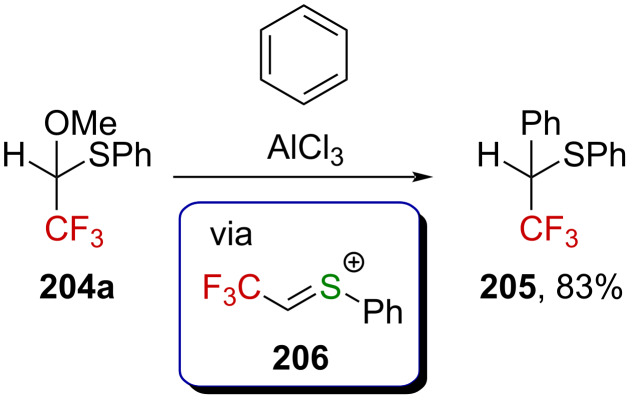
Reactivity of **204a** with benzene in the presence of a Lewis acid.

The related thioacetal **204a** was also studied and reacts with benzene upon treatment with strong Lewis acids (best with AlCl_3_) [139]. In this case, the only product formed in the course of the reaction was **205**, isolated in 83% yield (Scheme 50). The proposed cationic intermediate in this reaction is a sulfur-stabilized α-(trifluoromethyl)carbenium ion **206** (an α-(trifluoromethyl)-substituted sulfonium cation).

**Scheme 51:**
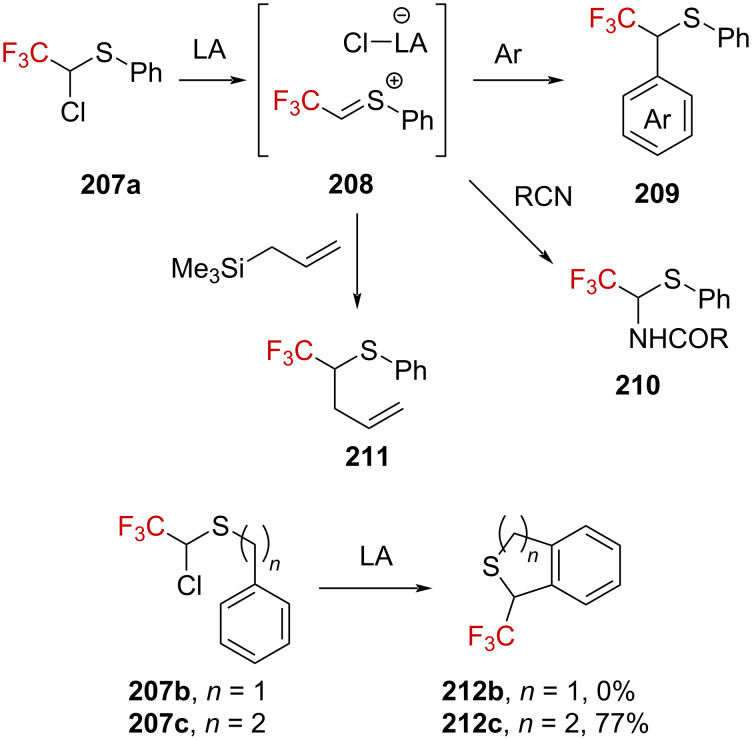
Reactivity of α-(trifluoromethyl)-α-chloro sulfides in the presence of strong Lewis acids.

Analogous to thioacetals **204a**, chloroalkylthio derivatives **207a**–**c**, bearing an adjacent CF_3_ group, were also investigated [140]. It appeared that a sulfur-stabilized α-(trifluoromethyl)carbenium ion **208** can be generated from **207a** by chloride abstraction following Lewis acid activation (e.g., SnCl_4_ or ZnCl_2_), opening an avenue for this cation to react with various nucleophiles (Scheme 51). Such a cation can also be trapped intramolecularly by a phenyl moiety; however, the length of the appended alkyl chain appeared to be of the utmost importance in this transformation.

**Scheme 52:**
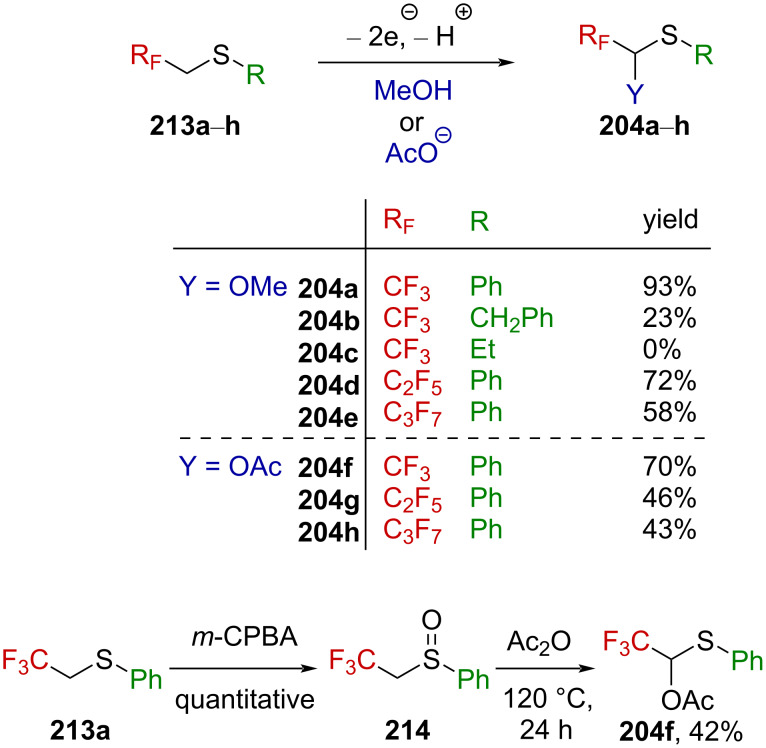
Anodic oxidation of sulfides **213a**–**h** and Pummerer rearrangement.

Analogous to their work on the nitrogen counterparts (vide supra), Fuchigami et al. were successful in the electrochemical production of sulfur-stabilized α-(trifluoromethyl)carbenium ions [139,141]. Thereby, they converted sulfides **213a**–**h** into thioacetals **204a**–**h** (Scheme 52). It is worth to note that the presence of an aromatic substituent on the sulfur atom is essential for the sulfides to react. Also, lengthening the perfluoroalkyl chain from CF_3_ to C_2_F_5_ or C_3_F_7_ resulted in a significant drop in the yield. Interestingly, while the electrochemical acetoxylation of **213a** furnished **204a** in an excellent yield of 93%, the Pummerer rearrangement of sulfoxide **214** under harsh conditions turned out to be less efficient, affording **204f** in only 42% yield.

**Scheme 53:**
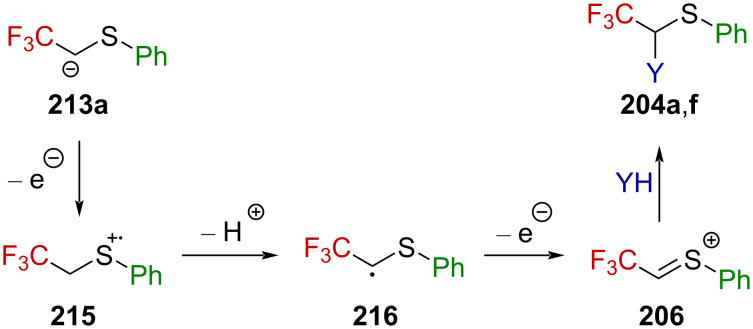
Mechanism for the electrochemical oxidation of the sulfide **213a**.

This reaction is thought to proceed stepwise via a first oxidative electron transfer, followed by deprotonation, a second oxidative electron transfer, and methoxylation or acetoxylation, respectively (Scheme 53). The driving force in this reaction is assumed to be the deprotonation of radical cation **215**, a highly destabilized species due to the presence of the strongly electron-withdrawing CF_3_ substituent, which leads to radical **216**, synergistically stabilized by the electron-withdrawing CF_3_ group and the electron donor sulfur atom through a captodative effect. Further oxidative electron transfer produces α-(trifluoromethyl)-substituted sulfonium ion **206**, leading to **204a**,**f** after reacting with the solvent.

### α-(Trifluoromethyl)alkylcarbenium ions

**Scheme 54:**
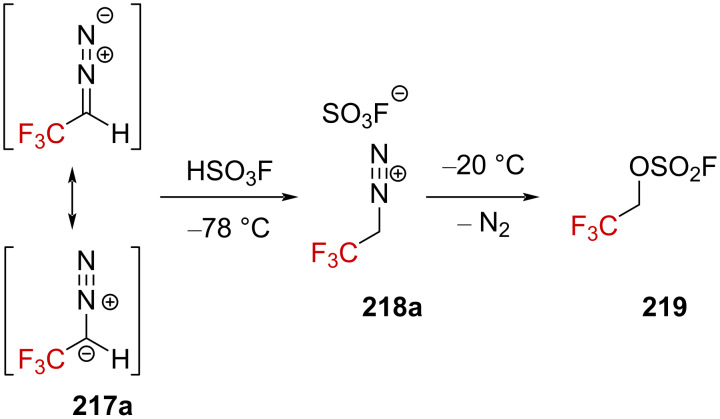
Reactivity of (trifluoromethyl)diazomethane (**217a**) in HSO_3_F.

**Hypothetical formation of CF_3_-containing alkylcarbenium ions from diazonium salts:** In 1967, Mohrig et al. successfully observed the first aliphatic diazonium ion **218a** by protonation of the corresponding diazo precursor [142] **217a** in a superacid by in situ NMR spectroscopy (Scheme 54) [143]. The remarkable characteristic of this strategy was the installation of a CF_3_ group in the α-position of the N_2_ moiety. This strategy relies on the high electron-withdrawing effect of the CF_3_ group, which greatly destabilizes nearby positive charges. As a result, the dissociation rate for the generation of molecular nitrogen was considerably reduced, allowing the observation of the diazonium ion at a low temperature. However, warming the diazonium solution up to −20 °C resulted in a vigorous evolution of N_2_ gas along with the clean formation of the resulting fluorosulfonate **219**, with no direct observation of the α-(trifluoromethyl)carbenium ion.

**Figure 10:**
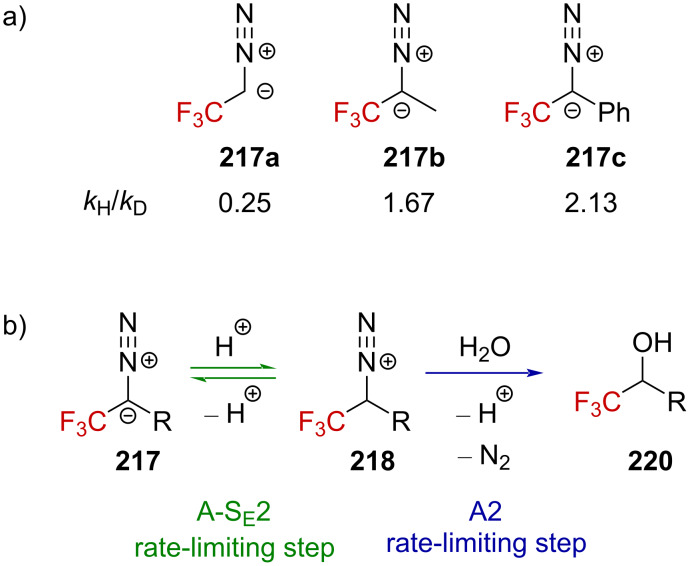
a) Structure of diazoalkanes **217a**–**c** and b) rate-limiting steps of their decomposition.

Further studies were conducted by Lenoir and Dahn to shed light on the mechanism of the solvolysis of CF_3_-substituted diazoalkane derivatives (Figure 10a) [144]. They measured an inverse kinetic isotope effect of *k*_H_/*k*_D_ = 0.25 for the solvolysis of **217a** in dioxane/H_2_O 60:40 in the presence of HClO_4_ (3 ≤ pH ≤ 4) and mentioned that this low value is “typical of a preequilibrium protonation reaction” and the rate-limiting solvolysis of diazonium ion **218a** (Figure 10b, in blue). Furthermore, the addition of a strong nucleophile dramatically increased the rate. The authors thus concluded that these observations are pieces of evidence for an A2 bimolecular process, which is also in agreement with the preferred decomposition pathway of other deactivated diazoalkanes (i.e., diazoacetate, *k*_H_/*k*_D_ = 0.34) [145–146]. Extending the investigations to diazo compound **217b** led to a different conclusion as a “normal” isotope effect of *k*_H_/*k*_D_ = 1.67 was obtained in this case. Diderich found a comparable ratio of *k*_H_/*k*_D_ = 2.13 for diazo compound **217c** [147]. In these latter cases, the solvolysis of diazoalkanes **217b** and **217c** is supported by an A-*S*_E_2 mechanism including a rate-limiting proton transfer (Figure 10b, in green) as the solvolysis rate approximately corresponds to the transfer rate of a proton (or deuteron). The difference in the reactivity between **217a** and **217b**,**c** would thus be due to the easier protonation of **217b**,**c** compared to **217a**, in a similar way as to how one can expect secondary carbanions to be more basic than primaries.

**Scheme 55:**
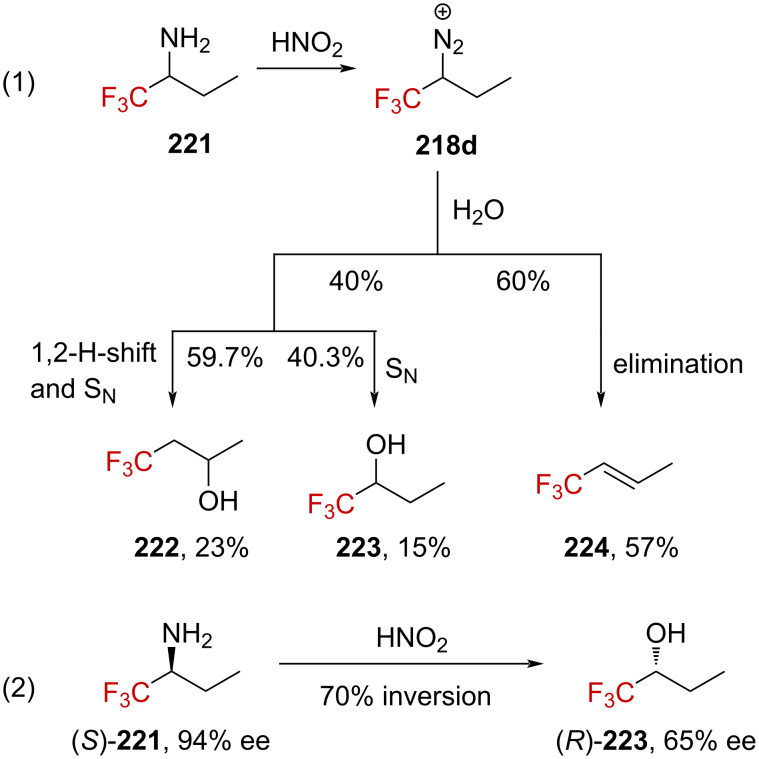
Deamination reaction of racemic **221** and enantioenriched (*S*)-**221**.

Studies on CF_3_-substituted diazonium ions were next conducted by Kirmse and Gassen to determine the solvolysis mechanism [148]. They found that upon deamination of **221** using a solution of sodium nitrite in aqueous perchloric acid at pH 3.5, a 60:40 mixture of the elimination product **224** and alcohols **222** and **223** was obtained in a 95% overall yield. These alcohols result from either solvolysis (**223**, 40.3%) or rearrangement (**222**, 59.7%, reaction (1) in Scheme 55). Further investigations on the stereochemical aspects leading to product **223** showed that when enantioenriched amine (*S*)-**221** (94% ee) was subjected to deamination, product (*R*)-**223** was obtained, with an inverted configuration and an eroded enantiomeric purity of 65% ee (reaction (2) in Scheme 55). The authors thus concluded that the formation of (*R)*-**223** from (*S*)-**221** occurred by a nucleophilic substitution mechanism, with 70% inversion. Since the racemization via a diazo↔diazonium equilibrium was excluded due to negligible ^2^D incorporation (i.e., <1%) when D_2_O was used, the 30% racemization noted in the process would account for the transient formation of a trifluoromethyl-substituted carbenium ion.

**Scheme 56:**
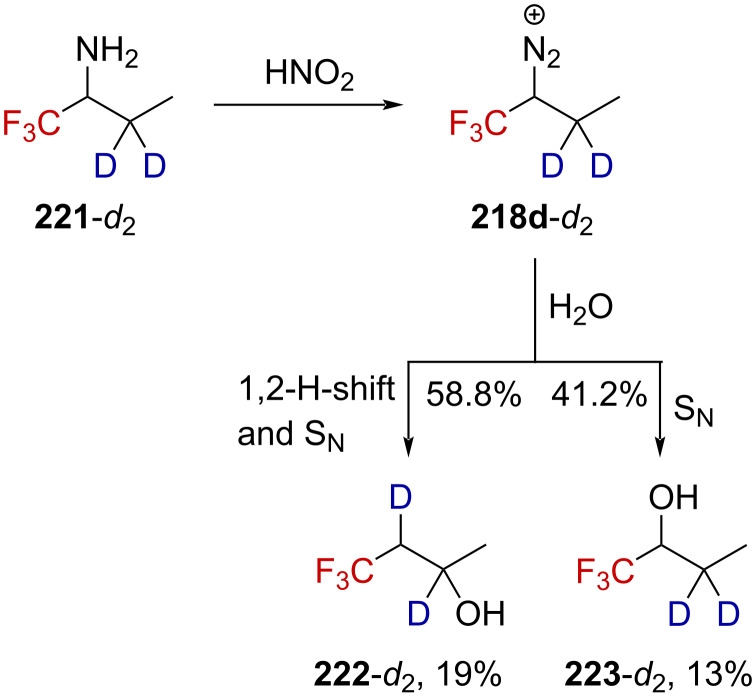
Deamination reaction of labeled **221**-*d*_2_. Elimination products were formed in this reaction, the yield of which was not determined.

Attempts to elucidate the mechanism for the formation of **222** revealed that deuterium-labeled **221**-*d*_2_ furnished products **223**-*d*_2_ and **222**-*d*_2_ upon deamination in a similar ratio and yield (Scheme 56, 41.2:58.8, 32%) as for the unlabeled **221** (Scheme 55, 40.3:59.7, 38%). This is a strong evidence for the transient formation of a carbenium ion as the isotope effect for the 1,2-H-shift is known to be very small in carbenium ions. It has been indeed previously demonstrated that a 1,2-H-shift isotope effect of *k*_H_/*k*_D_ = 1.2–1.3 was obtained starting from 2-butyldiazonium ion **225**, which is known to decay via a carbenium ion [149–150].

**Scheme 57:**
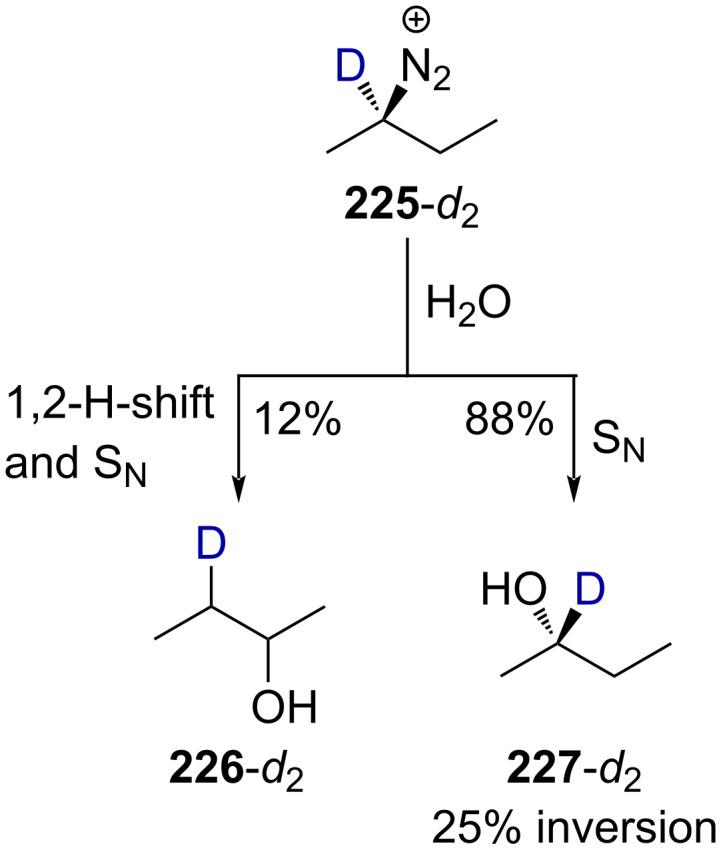
Deamination reaction of **225**-*d*_2_. Elimination products were also formed in this reaction in undetermined yield.

In the absence of the CF_3_ group, **225**-*d*_2_ decays in a mixture of alkenes and alcohols. By taking only the alcohol mixture into account, alcohol **227**-*d*_2_ was considered to have been obtained via a nucleophilic substitution mechanism (88%) with 25% inversion and **226**-*d*_2_ via rearrangement (12%, Scheme 57). This contrasts with the previous results obtained for **218d**, which lead to 40.3% of the nucleophilic-substitution product **223** with 70% inversion and 59.7% of rearranged **222** when only considering the mixture of alcohols (reaction (1) in Scheme 55).

**Scheme 58:**
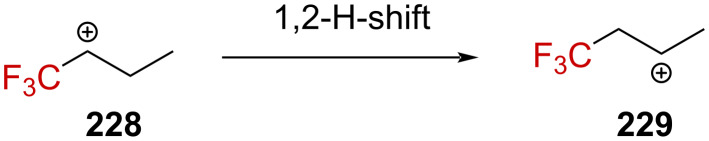
Formation of **229** from **228** via 1,2-H-shift.

This would be consistent with a less labile C–N bond in **218d** and the formation of the extremely reactive α-(trifluoromethyl)carbenium ion **228** that is therefore more prone to undergo rearrangements to generate the more stabilized β-(trifluoromethyl)carbenium ion **229** (Scheme 58).

**Scheme 59:**
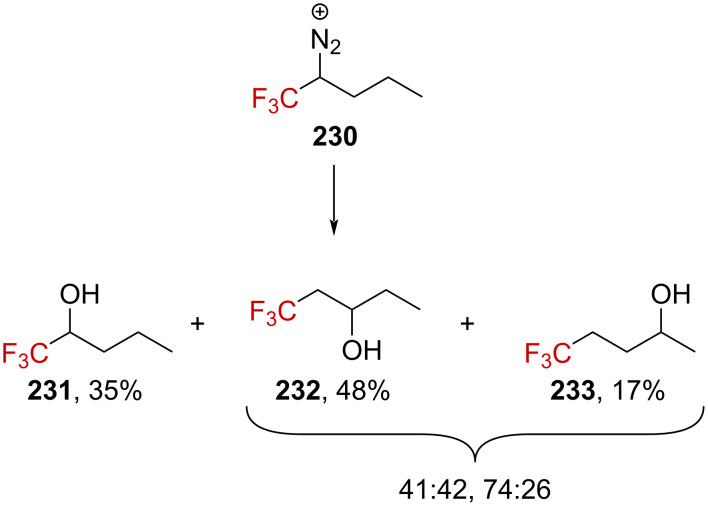
Deamination reaction of **230**. Elimination products were formed in this reaction, the yield of which was not determined.

Further rearrangements were confirmed by the authors when alcohol **233**, resulting from a twofold 1,2-H-shift, was generated from diazonium salt **230** (Scheme 59).

**Scheme 60:**
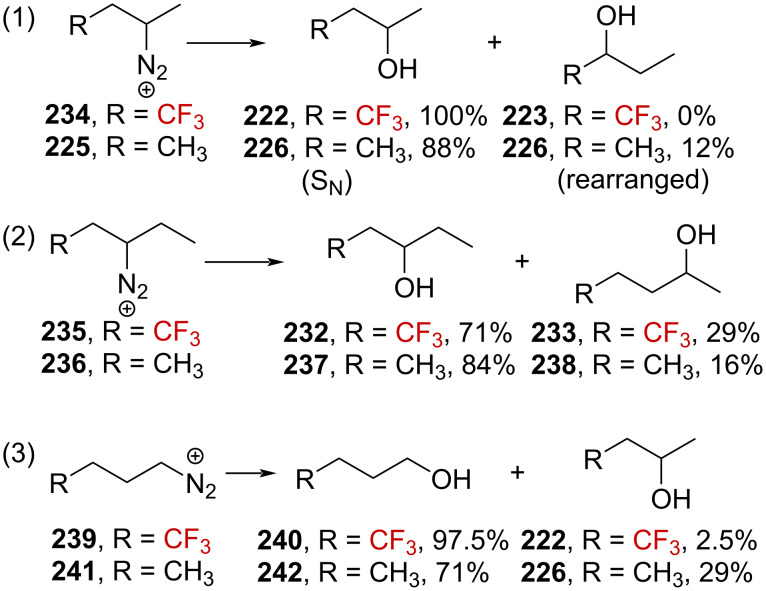
Deamination of several diazonium ions. Elimination products were formed in these reactions, the yield of which was not determined.

The β- and γ-CF_3_ effects on the carbenium ions were also investigated by the same authors by systematically comparing the reactivity of a selected series of CF_3_-containing and analogous nonfluorinated diazonium ions toward solvolysis. The diazonium ion **234** led exclusively to alcohol **222**, with the absence of any detectable rearranged products, while the CF_3_-free analogous species **225** underwent 12% rearrangement (reaction (1) in Scheme 60). The diazonium ion **235** furnished alcohols **232** and **233** in a 71:29 ratio, without the detectable formation of α-(trifluoromethyl) alcohol **231**, while the analogous compound **236** provided **237** and **238** in a 84:16 ratio (reaction (2) in Scheme 60). Similarly, the terminal diazonium ion **239** decayed to produce a 97.5:2.5 ratio of alcohols **240** and **222**, a very different behavior than for **241**, which produced **242** and **226** in a 71:29 ratio (reaction (3) in Scheme 60).

Even though the direct observation of α-(trifluoromethyl)carbenium ions was not the purpose of this study, it successfully brought a better understanding on the effect of a CF_3_ group close to a positive charge.

**Scheme 61:**
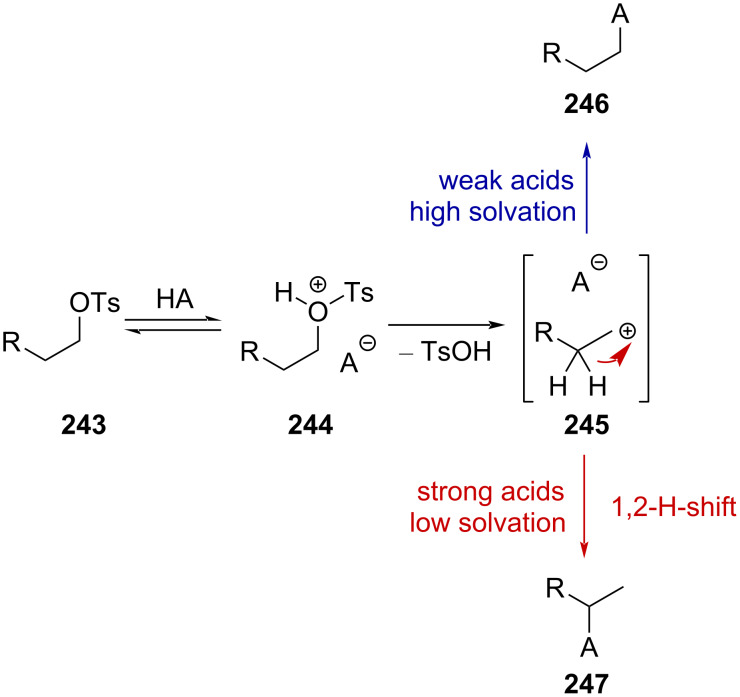
Solvolysis reaction mechanism of alkyl tosylates.

**Hypothetical formation of CF_3_-containing alkylcarbenium ions by activation of alcohol derivatives:** The solvolysis reaction of alkyl tosylates has attracted the attention of many chemists, and successive studies revealed that hydrogen or methyl shifts were effective and most prominent in strongly acidic solvents, such as HSO_3_F, with *H*_0_ = −15.1 [151] (Scheme 61) [152–154]. This is the result of the lack of solvation of intermediate carbenium ion **245** in strong acids due to the high ionizing power and low nucleophilicity, favoring the stabilization by hyperconjugation, followed by 1,2-H-shift [155].

**Scheme 62:**
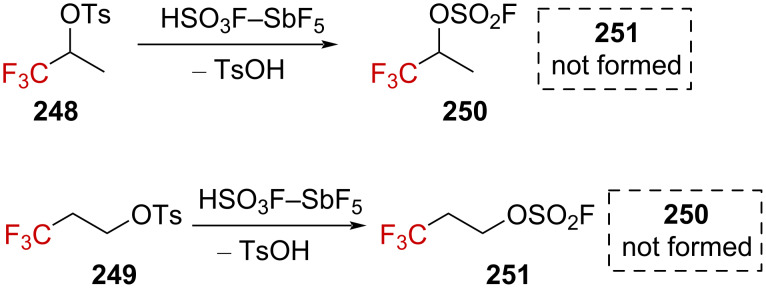
Solvolysis outcome for the tosylates **248** and **249** in HSO_3_FSbF_5_.

In this context, Myhre and Andrews explored the reaction of α- and β-(trifluoromethyl) tosylates **248** and **249** in strongly acidic solvents (Scheme 62) [156]. Contrary to what could have been expected, no rearranged products were formed in either case, even in magic acid, HSO_3_F–SbF_5_ (*H*_0_ = −23 [151]).

**Figure 11:**
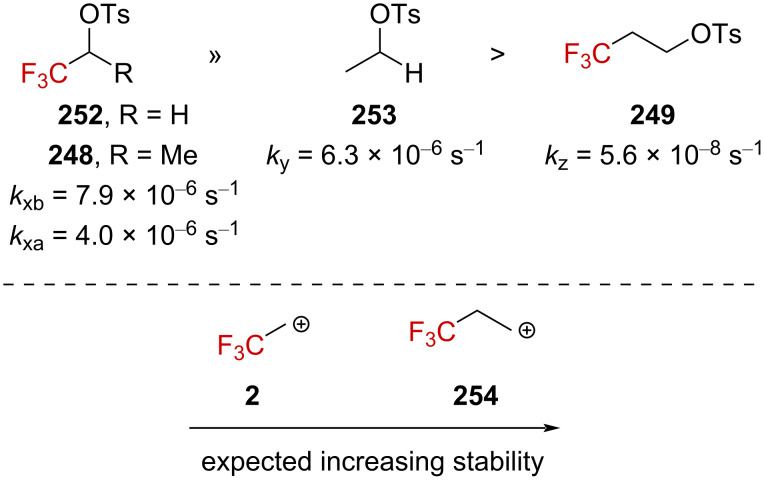
Solvolysis rate of **248**, **249**, **252**, and **253** in 91% H_2_SO_4_.

The solvolysis study on aliphatic trifluoromethyl tosylate derivatives in strong acids was conducted following theoretical studies [156–157]. While **248** and **252** showed a solvolysis rate comparable to that of **253** in 85–100% H_2_SO_4_, derivative **249** underwent solvolysis at a significantly slower rate (Figure 11). This counterintuitive behavior was not considered to be in line with the intermediary formation of a carbenium ion, as β-(trifluoromethyl)carbenium ion **254** generated from **249** is expected to be more stable than α-(trifluoromethyl)carbenium ion **2** generated from **252**.

**Scheme 63:**
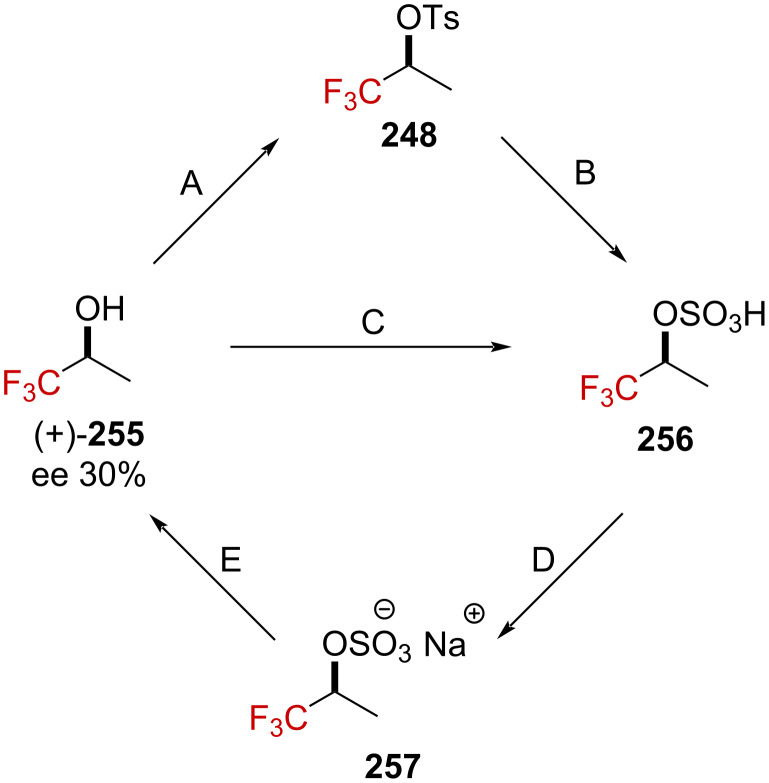
Illustration of the reaction pathways. TsCl, pyridine, −5 °C (A); 98% H_2_SO_4_, 30 °C (B); 98% H_2_SO_4_, 30 °C (C); NaOH (aq), evaporation, extraction with MeOH (D); and moist Et_2_O–H^+^, reflux (E).

To rationalize this trend under these reaction conditions, the authors submitted the enantioenriched alcohol (+)-**255** ([α]_365_^25^ +2.682, the absolute configuration was not mentioned) to two distinct reaction pathways (Scheme 63). No erosion of the specific rotation, neither through path ABDE ([α]_365_^25^ +2.692), nor CDE ([α]_365_^25^ +2.679) was observed, suggesting that an α-(trifluoromethyl)carbenium ion cannot be considered as a reactive intermediate.

**Scheme 64:**
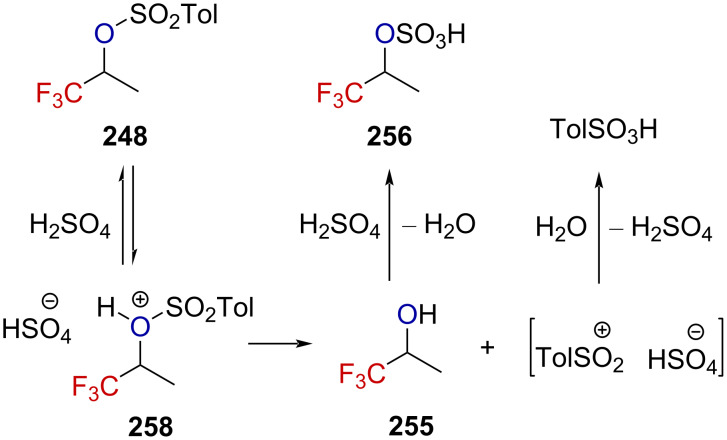
Proposed solvolysis mechanism for the aliphatic tosylate **248**.

Further labeling experiments revealed that the ^18^O percentage in ^18^O-**255** (24.6% ± 0.3%) remained unchanged before and after being subjected to the path A–B–D–E (24.4% ± 0.3%) or C–D–E (24.3% ± 0.3%). Hence, no C–O bond cleavage happens in any of these steps. The authors rationalized the experimental observations by invoking a dissociation mechanism involving the cleavage of the weak O–S bond, as depicted in Scheme 64. These experimental results strongly oppose those collected by Tidwell and Koshy [39] on benzylic α-(trifluoromethyl)-substituted tosylate derivatives (see section on α-(trifluoromethyl)-substituted carbenium ions), presumably due to the presence of a stabilizing phenyl moiety in the latter case.

**Scheme 65:**
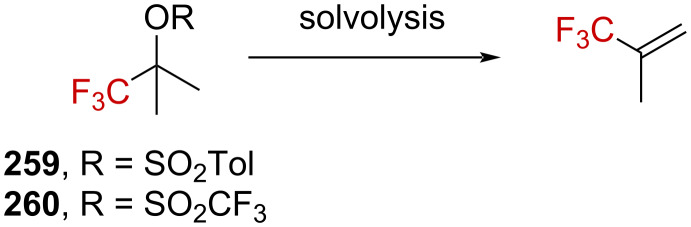
Solvolysis of the derivatives **259** and **260**.

Analogous investigations on triflate derivatives were realized by Tidwell et al. [41]. Triflates are more reactive than tosylates – as illustrated by *k*_Tf_/*k*_Ts_ = 7 × 10^4^ for the elimination reactions of **259** and **260** – and were thus of interest in the context of solvolysis studies. The solvolysis of **260** in various solvents led to the sole formation of the elimination product, and no nucleophilic substitution of the triflate by the solvent was observed. Similar results were also reported previously by the authors for **259** (Scheme 65) [39]. Interestingly, no dependence of the elimination rate on the ionizing power of the solvents was observed, suggesting that the formation of an ion pair (either intimate or solvent-separated) was not the limiting step. However, the faster rate obtained in the most nucleophilic solvents implies that the solvent is involved in the rate-limiting step.

Kinetic isotope effects in the elimination reactions of **260**, **260**-*d*_3_, and **260**-*d*_6_ were found to be *k***_260_**/*k***_260_**_-_*_d_*_3_ = 1.78 and *k***_260_**/*k***_260_**_-_*_d_*_6_ = 3.80. The effect of the solvents and added salts on the rate proved that the medium (solvent and salt) is involved in the rate-limiting step. Furthermore, the values obtained for the secondary isotope effect agreed with the elimination as the rate-limiting step and strongly support the hypothesis that the latter occurred from an intimate ion pair.

**Scheme 66:**
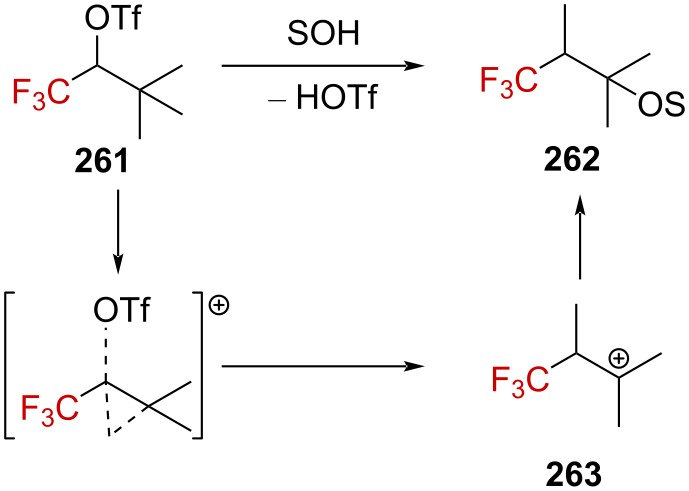
Solvolysis of triflate **261**. SOH = solvent.

Starting from **261**, no elimination product could be formed during the solvolysis reaction, and a 1,2-methyl shift occurred to generate **262** after solvent trapping, as reported by Roberts and Hall (Scheme 66) [158]. Kinetic studies revealed a linear free-energy relationship between the rate of the solvolysis against the *Y*_OTf_ values. The isolated product **262** as well as the kinetic data strongly support the formation of the β-(trifluoromethyl)carbenium ion **263** in the rate-limiting step with considerable neighboring group participation, characteristic of a *k*_Δ_ pathway.

**Scheme 67:**
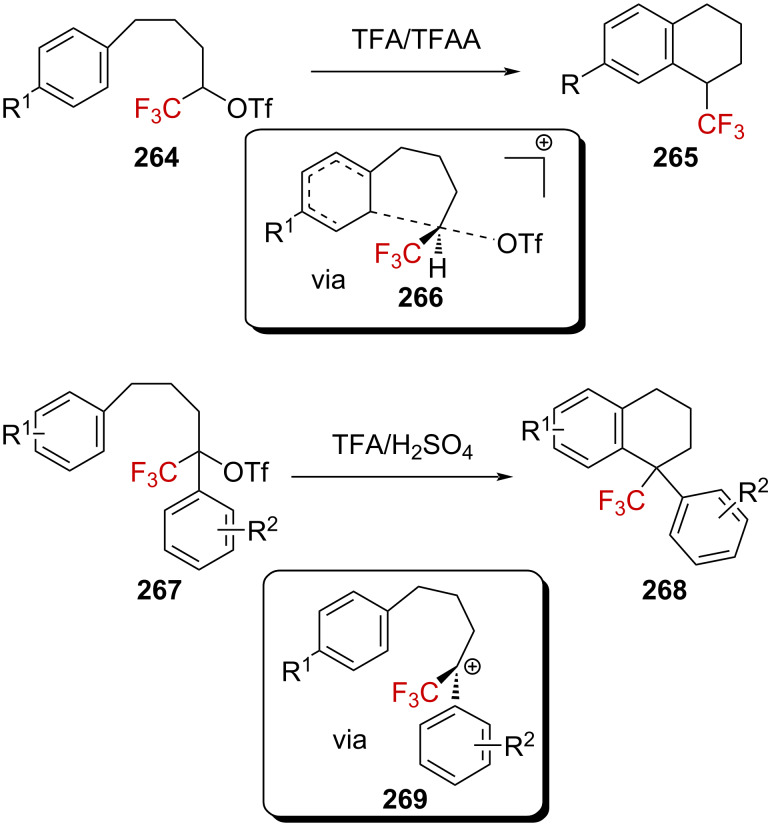
Intramolecular Friedel–Crafts alkylations upon the solvolysis of triflates **264** and **267**.

Bonnet-Delpon et al. successfully took advantage of the intramolecular stabilization of a cation induced by the presence of a CF_3_ group to develop a method to access 1-(trifluoromethyl)tetralins [159]. For instance, upon the solvolysis of systems such as **264** in TFA/TFAA, the cyclized products **265** were obtained. Furthermore, it is known that the nontrifluoromethylated tosylate analogue undergoes the same cyclization via a *k*_Δ_ process rather than a *k*_c_ process [160]. The authors thus proposed that the aryl ring stabilizes the cation concomitantly after the elimination of the triflate anion to form the transition state **266** in the solvolysis reaction of derivatives **264**. The same cyclization reaction occurred when derivatives such as **267** were solvolyzed in TFA/H_2_SO_4_, affording **268** (Scheme 67). However, while the nature of the aryl substituent R^1^ had a negligible effect on the rate, the latter had a convincing dependence on the nature of the substituent R^2^. For benzylic systems **267**, the authors proposed a *k*_c_ pathway involving the formation of the more stable benzylic α-(trifluoromethyl)carbenium ion **269**, with a subsequent cyclization reaction.

**Scheme 68:**
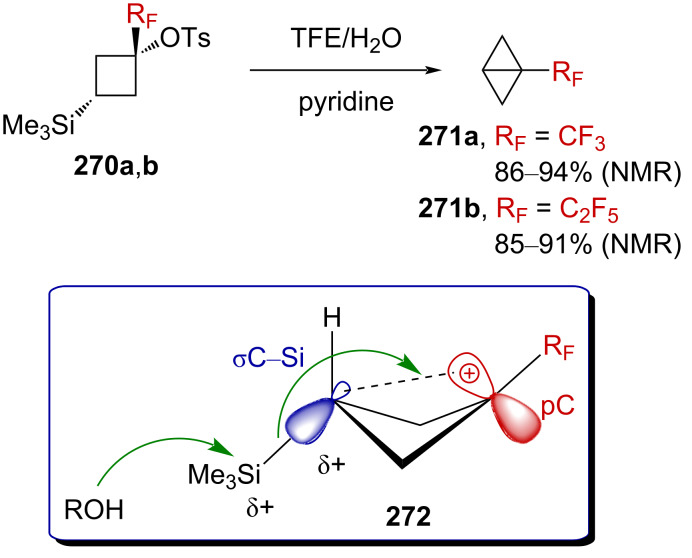
α-CF_3_-enhanced γ-silyl elimination of cyclobutyltosylates **270a**,**b**.

Gassman and Doherty suggested that the introduction of a strongly electron-withdrawing group in the α-position of a positively charged carbon center could magnify the neighboring group participation so as to compensate for the increased electron deficiency at the incipient cationic center [4,161]. Using this strategy, Tilley et al. reported the first synthesis of strained CF_3_-substituted bicyclo[1.1.0]butane **271a** via γ-silyl elimination of α-(trifluoromethyl)cyclobutyl tosylate **270a** (Scheme 68) [162]. The reaction was proposed to occur via neighboring-group participation of the silicon-based group, through homohyperconjugative stabilization of the pC orbital of the incipient α-R_F_-substituted carbenium ion by a percaudal (back lobe) participation of the σC–Si orbital (**272**, Scheme 68). Importantly, the initial W-conformation in the starting material **270a**,**b** was mandatory to allow a sufficient orbital overlap as the U-conformation (*endo*-sickle-like isomer) failed to react within the reaction time (≈12 h). In **272**, the positive charge is thus significantly delocalized at the silicon center, allowing a facile nucleophilic displacement at the silicon atom by a solvent molecule to afford **271a**,**b**. The CF_3_ moiety strongly affects the stability in **271a**, which was found to be stable “indefinitely” when stored under an inert atmosphere at a low temperature and did not suffer from polymerization.

**Scheme 69:**
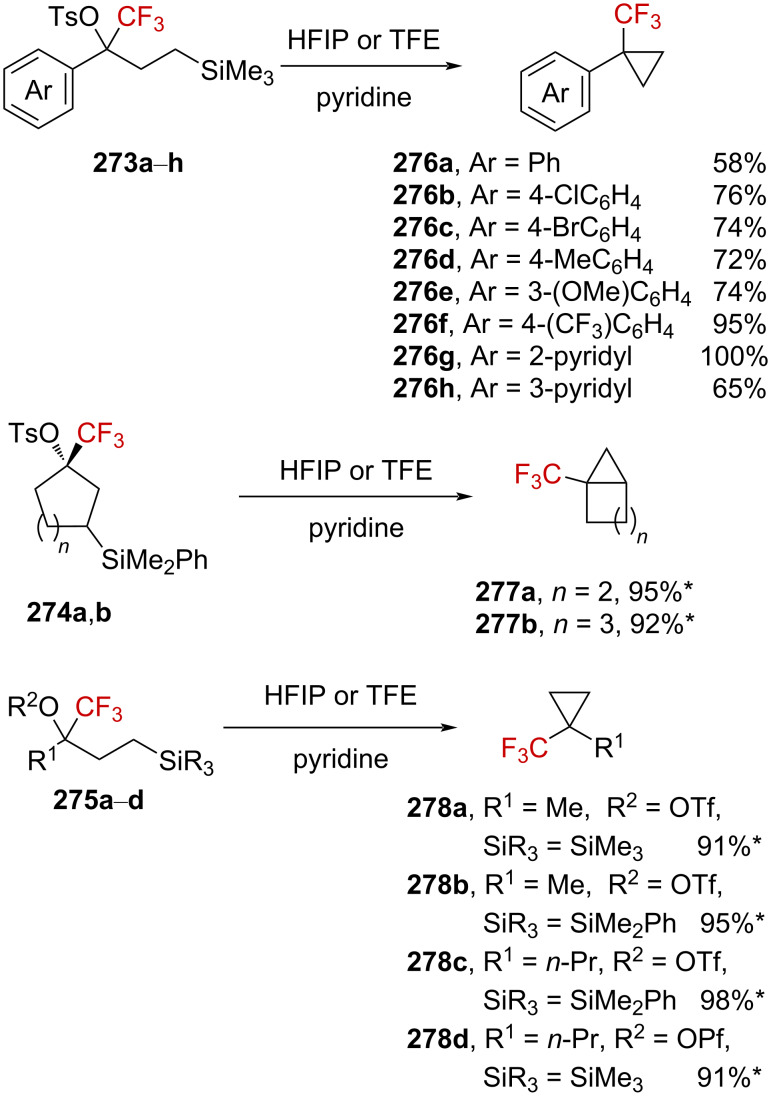
γ-Silyl elimination in the synthesis of a large variety of CF_3_-substituted cyclopropanes. Pf = pentafluorophenylsulfonate. For **277c** and **276g**, no pyridine was used. For **276g**, the yield refers to the protonated pyridinium tosylate. *NMR yield.

Further investigations by Tilley et al. were conducted in order to enlarge the scope of the above-mentioned 1,3-silyl elimination of α-(trifluoromethyl) tosylate, which was restricted so far to cyclobutyl derivatives, and a variety of linear or cyclic α-(trifluoromethyl)-γ-silyl sulfonates was targeted (Scheme 69) [163–164]. While the solvolysis was readily performed with tosylate-like leaving groups in the case of aromatic substituents being present, as in **273a**–**h**, or in the cyclic systems **274a**,**b**, a better leaving group, such as triflate, was generally required for alkyl derivatives **275a**–**d**.

**Scheme 70:**
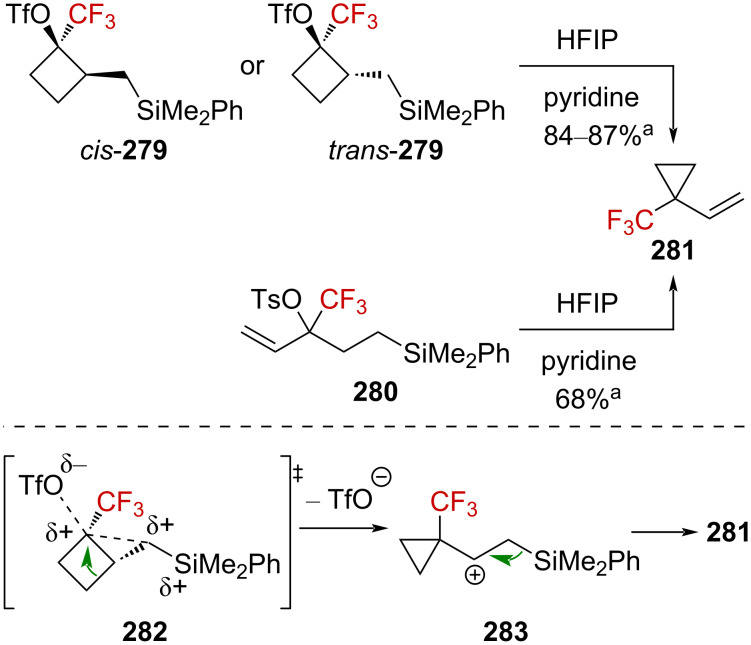
Synthetic pathways to **281**. ^a^NMR yields.

Interestingly, CF_3_-substituted cyclopropanes **281** could be obtained from linear derivative **280** but also from cyclic **279** (*cis*-**279** or *trans*-**279**) via an alternative mechanism. The proposed mechanism for the conversion of **279** into **281** invokes an alkyl shift, leading to the generation of a carbenium ion **283**, stabilized by the β-effect of silicon (via the transition state **282**), and further β-silyl elimination affords product **281** (Scheme 70). In addition, *trans*-**279** reacted approximately 12 times faster than *cis*-**279**, and thus suggesting a neighboring-group participation via the σC–Si orbital in the proposed transition state **282**.

**Scheme 71:**
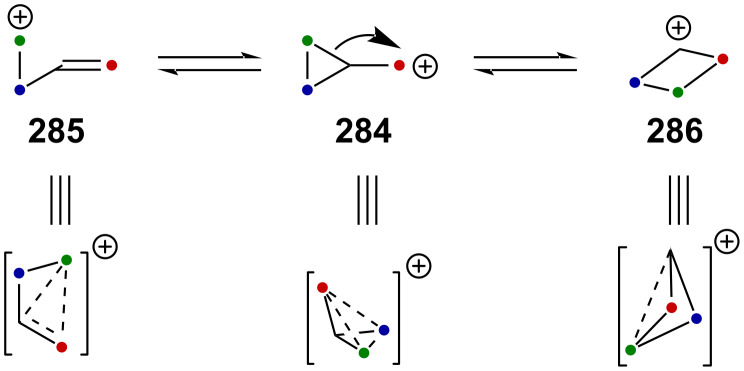
The cyclopropyl-substituted homoallylcyclobutylcarbenium ion manifold.

Very recently, Creary reported a study on the generation of CF_3_-subtituted γ-silylcarbenium ions via a cyclopropylcarbinyl rearrangement [164]. When cyclopropylcarbinylcarbenium ion **284** is generated, this species is in an equilibrium with the homoallylcarbenium and cyclobutylcarbenium ions **285** and **286** (Scheme 71) [164].

**Scheme 72:**
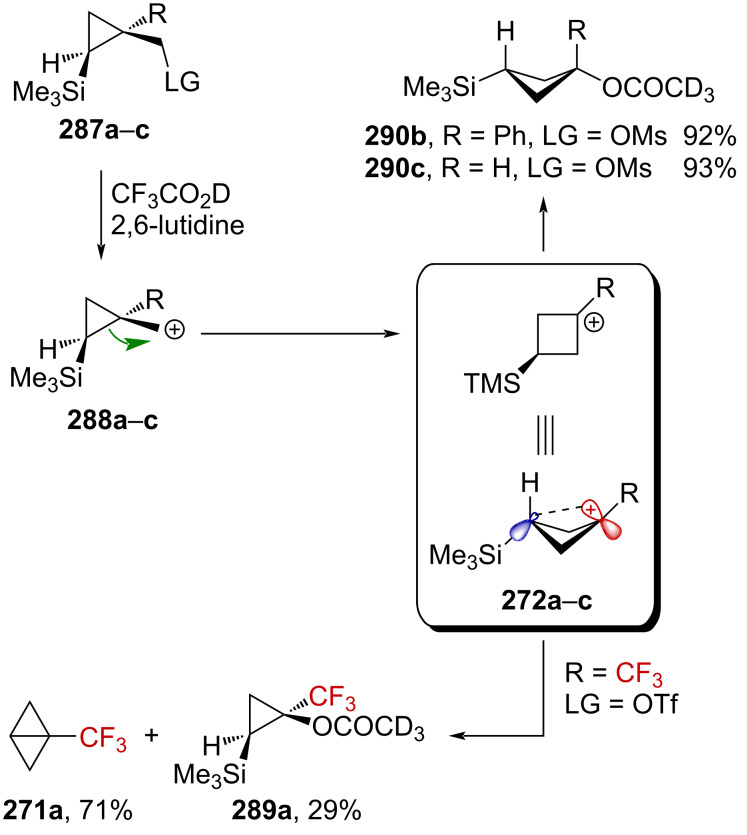
Reactivity of CF_3_-substituted cyclopropylcarbinyl derivatives **287a**–**c**. LG = leaving group.

Creary investigated the solvolysis of CF_3_-substituted cyclopropylcarbinyl triflate **287a** and obtained a mixture of bicyclobutane **271a** and unrearranged solvent-substitution product **289a** in 71% and 29% yield, respectively (Scheme 72) [164]. This result was in stark contrast with those obtained with Ph- and H-substituted analogues **287b** and **287c** because the main products of the reactions in the latter cases were cyclobutanes **290b** and **290c**. As mentioned previously, this is the result of an enhanced neighboring-group participation induced by the presence of the CF_3_ group in **287a**. A stronger percaudal stabilization is thus present in carbenium intermediate **272a**, which leads mainly to **271a** by solvent-assisted γ-silyl elimination.

**Scheme 73:**
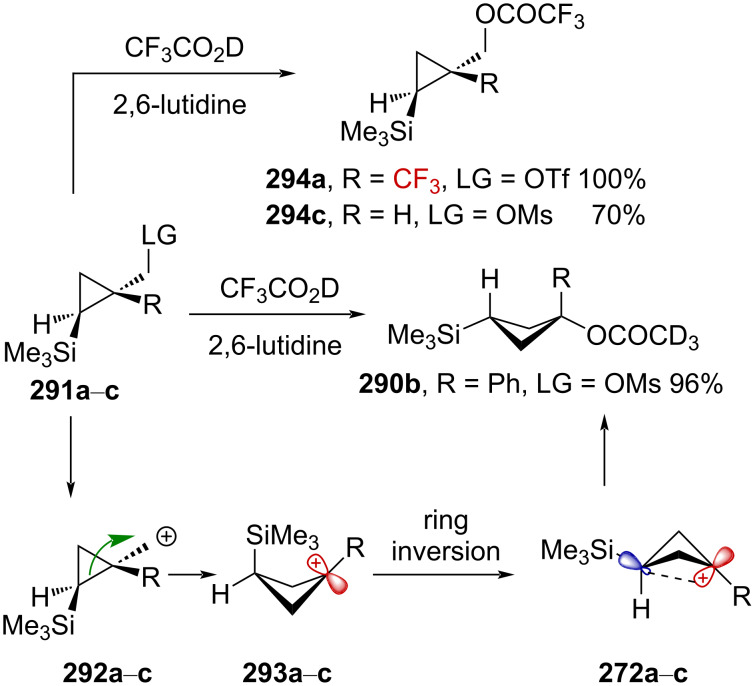
Reactivity of CF_3_-substituted cyclopropylcarbinyl derivatives **291a**–**c**.

Creary then considered the diastereomers of **287a**–**c**, namely **291a**–**c**. While **291b** led to the same product **290b**, the isomer **290a** and unsubstituted **290c** exhibited a different reactivity as they did not form the rearranged cyclobutane derivatives **290a** and **290c** (Scheme 73) [164]. It was mentioned that for isomers **291a**–**c**, the conformation of the corresponding cyclobutylcarbenium ions **293a**–**c** after the rearrangement would not allow the percaudal participation of the TMS group. Nevertheless, in the presence of a stabilizing group, such as a phenyl group, carbenium ion **293b** is sufficiently stable and can undergo ring inversion to furnish carbenium ion **272b**, stabilized by the TMS group, which finally gives **290b**. On the other hand, in the presence of a CF_3_ group or a H atom, **291a** and **291c** strongly suffer from the absence of this stabilization and are mainly converted to the unrearranged products **294a** and **294c**.

**Scheme 74:**
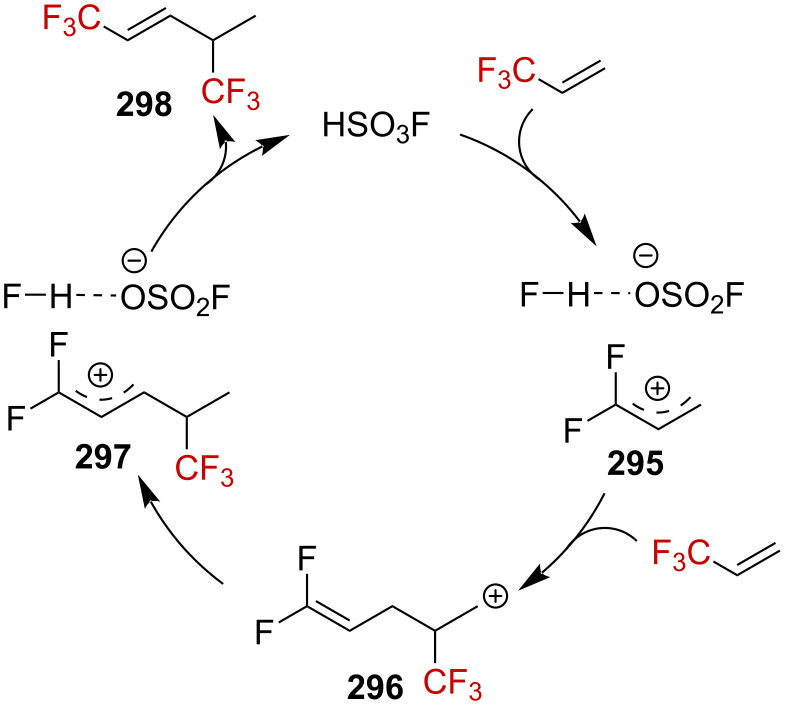
Superacid-promoted dimerization or TFP.

**Hypothetical formation of CF_3_-containing alkylcarbenium ions by alkene activation:** Because 1,1,1-trifluoropropene (TFP) undergoes an anti-Markovnikov addition in the presence of hydrogen halide, Myhre and Andrews anticipated that a similar regioselectivity may occur with HSO_3_F [156]. Submitting the fluorinated olefin to HSO_3_F unexpectedly led to a dimerization of TFP. The provided mechanistic explanation involves a C–F activation by the HSO_3_F Brønsted superacid to generate difluorinated allylcarbenium ion **295**. It must then react with another molecule of TFP to give **296** (Scheme 74). A subsequent 1,3-hydrogen shift, driven by the formation of an allylic carbenium ion **297** from a primary carbenium ion **296**, furnished the isolated product **298** after fluorine abstraction from the anion.

**Scheme 75:**
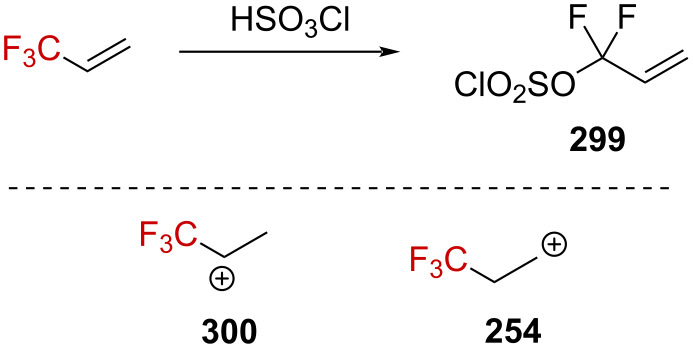
Reactivity of TFP in a superacid.

Further evidence for the formation of the putative difluorinated allylcarbenium ion **295** was obtained by dissolving TFP in less acidic HSO_3_Cl (*H*_0_ = −13.8 [151]). In this superacidic medium, difluoroallyl sulfonate **299**, resulting from the direct trapping of **295** by the more coordinating SO_3_Cl^−^ anion (compared to SO_3_F^−^), was smoothly formed (Scheme 75) [165]. Hence, this demonstrated that the C–F activation of the CF_3_ moiety to generate a difluoroallylcarbenium ion **295** was favored over the formation of a secondary α-CF_3_-substituted species **300** or a primary aliphatic β-(trifluoromethyl)carbenium ion **254**. Indeed, no evidence for the protonation of TFP was obtained, highlighting once more the extraordinary electron-withdrawing and deactivating potential of the CF_3_ moiety. It is worthy of note that the installation of an aryl group, however, makes the protonation of α-(trifluoromethyl)styrene derivatives possible, even though a retardation of the rate of 10^4^–10^7^ has been measured due to the presence of the CF_3_ group [68].

**Scheme 76:**
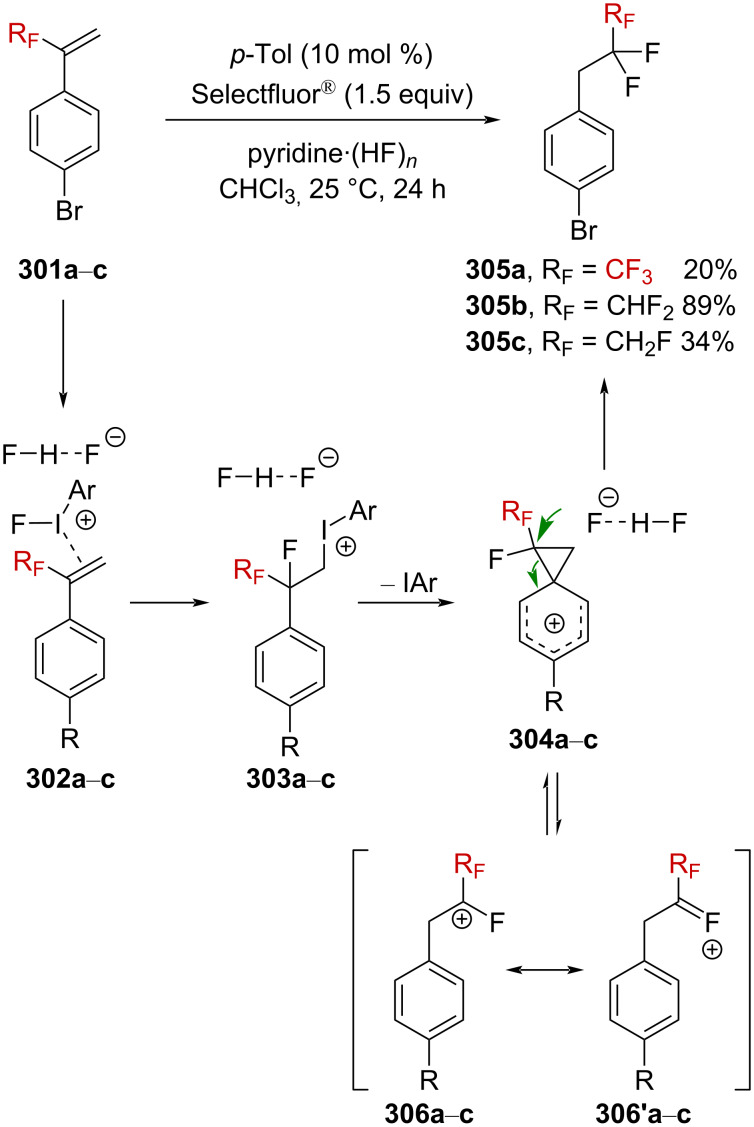
*gem*-Difluorination of α-fluoroalkyl styrenes via the formation of a “hidden” α-R_F_-substituted carbenium ion **306**↔**306’**.

To overcome the difficulty to generate trifluoromethyl-substituted alkylcarbenium ions after the activation of trifluoromethyl-substituted alkenes, the stabilization by a neighboring group could be envisaged. In the enantioselective *gem*-difluorination of styrenes catalyzed by hypervalent iodoarene species, Jacobsen et al. elegantly exploited the stabilizing effect of an aromatic ring through skeletal rearrangement via a phenonium ion intermediate [166]. Recently, Gilmour et al. synthesized highly fluorinated scaffolds using this strategy (Scheme 76) [167]. The widely accepted mechanism for this transformation involves a first fluoroiodination of an olefin **301a**–**c** to give **303a**–**c**, followed by an anchimerically assisted iodonium elimination to generate the phenonium ions **304a**–**c** and a subsequent regioselective fluoride addition to furnish compounds **305a**–**c** (Scheme 76) [168]. In this example, the phenonium species **304a**–**c** can be regarded as a “hidden” α-(trifluoromethyl)carbenium ion **306a**–**c**, in which the fluorine atom in the α position stabilizes the cation by lone pair back-donation (see **306’a**–**c**), favoring the whole process.

### α-(Trifluoromethyl)vinylcarbenium ions

**Scheme 77:**
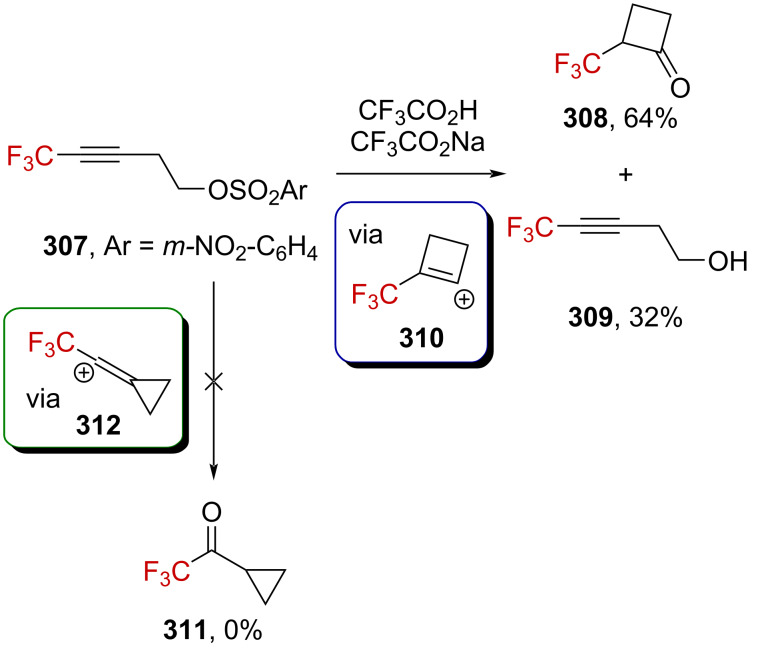
Solvolysis of CF_3_-substituted pentyne **307**.

The involvement of vinyl α-(trifluoromethyl)carbenium ions is scarcely reported in the literature. Vött et al. reported the synthesis of CF_3_-containing small rings via the transient formation of vinyl cations [169]. During the course of their study, they investigated the reactivity of CF_3_-substituted pentyne **307**. The solvolysis of **307** in TFA and CF_3_CO_2_Na led to cyclobutanone **308** and alcohol **309**. The isolation of **308** suggests the transient formation of β-(trifluoromethyl)vinyl cation **310**. However, no trace of a cyclopropyl ketone **311** was observed, indicating that this route is prohibited as it requires the generation of a more destabilized α-(trifluoromethyl)vinyl cation **312** of higher energy (Scheme 77).

**Scheme 78:**
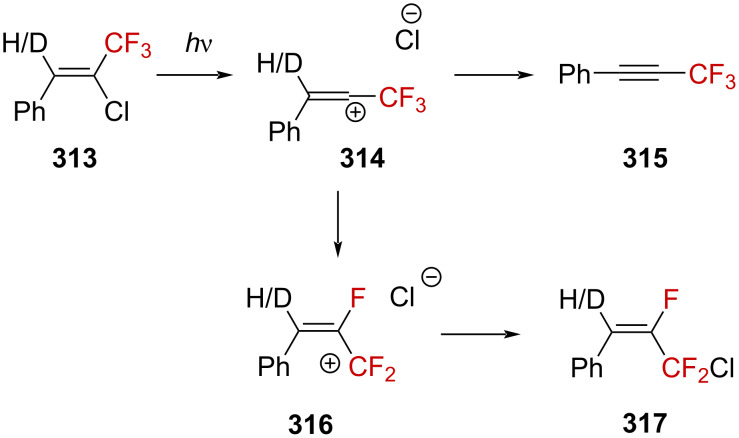
Photochemical rearrangement of **313**.

The photochemical formation of α-(trifluoromethyl)vinylcarbenium ions has also been suggested by Lodder et al. (Scheme 78) [170]. UV irradiation of vinyl compound **313** led to the formation of acetylene product **315**, which is suggested to be formed via β-H-elimination from an open α-(trifluoromethyl)vinylcarbenium ion **314**. A kinetic isotope effect study gave a *k*_H_/*k*_D_ = 1.22 ratio, which is in perfect agreement with β-secondary isotope effect values for reactions proceeding through a carbenium ion. The observation of product **317** strongly supports this cationic mechanism, as it is not unlikely that carbenium ion **314** undergoes a 1,2-fluorine shift (although such a rearrangement has not been experimentally demonstrated so far) to generate the more stable difluorinated allyl cation **316**, which leads to **317** after internal return. Noteworthy, it has been calculated that such a vinyl cation **314** is 42.1 kcal⋅mol^−1^ higher in energy than the corresponding CH_3_-substituted analogues.

### Nonclassical α-(trifluoromethyl)carbenium ions

**Figure 12:**
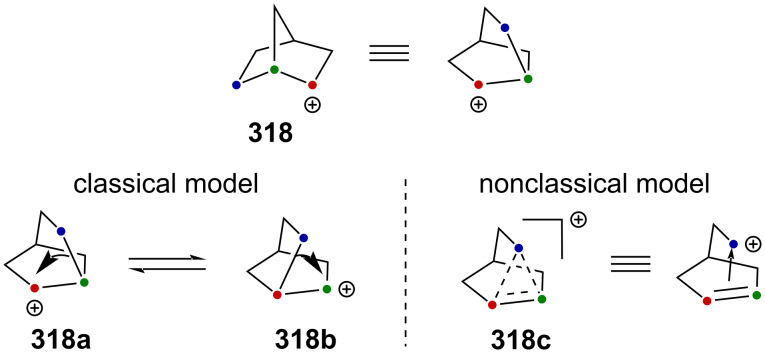
Structure of 2-norbornylcarbenium ion **318** and argued model for the stabilization of this cation.

The very existence of nonclassical carbocations (3 centers, 2π-electrons) has been the subject of debate for decades. The 2-norbornyl cation became the most emblematic example, and its structure has been proposed either as two carbenium ions, **318a** and **318b**, in a rapid equilibrium or as a symmetrical cation **318c**, displaying a nonclassical pentacoordinated carbon atom (Figure 12) [171–173]. Krossing et al. eventually put an end to this debate by achieving the crystal growth and crystal structure determination of the 2-norbornyl cation, the structure of which was unequivocally assigned as **318c** [174].

**Figure 13:**
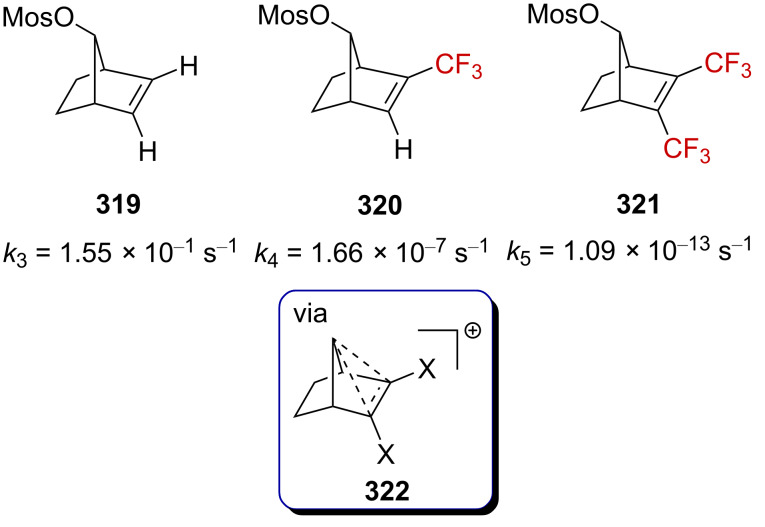
Structures and solvolysis rate (TFE, 25 °C) of the sulfonates **319**–**321**. Mos = *p*-MeOC_6_H_4_SO_2_.

In 1984, as part of their investigations on carbocation stabilization by neighboring group participation, Gassman and Hall brought evidence for the nonclassical model using a strategy involving a progressive destabilization of the resulting cation by the introduction of CF_3_ groups in the norbornene derivatives **319**–**321** (Figure 13) [175]. They found a cumulative effect of the CF_3_ groups on the solvolysis rate, with a 10^6^-fold decelerating effect upon the introduction of each CF_3_ unit. The authors concluded that “the fact that each CF_3_ group decreases the rate of ionization by 10^6^ provides overwhelming evidence that the interactions of the double bond […] with the incipient carbocation involve symmetrical (nonclassical) transition states **322**, rather than pairs of rapidly equilibrating (classical) cations”.

**Scheme 79:**
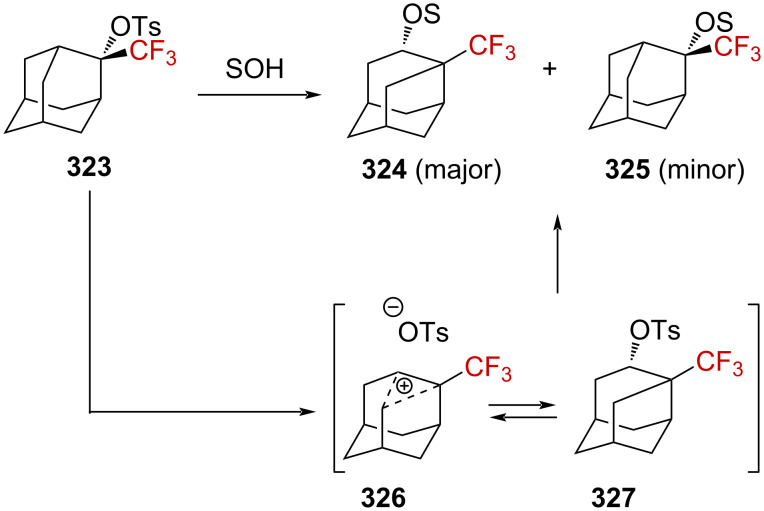
Mechanism for the solvolysis of **323**. SOH = solvent.

2-Adamantyl tosylate is one of the main references to describe the S_N_1 mechanism in which the carbenium character is maximized. For this reason, Prakash, Tidwell, et al. tried to reach the highest *k*_H_/*k*_CF3_ ratio by exploring 2-adamantyl-2-trifluoromethyl tosylate (**323**), which was expected to exhibit a profound reluctance to generate a carbenium ion [176]. Ironically, the solvolysis of **323** in several solvents led to an average ratio of *k*_H_/*k*_CF3_ = 2.0, the smallest ratio ever obtained to date. The explanation for this unprecedented high reactivity for an α-(trifluoromethyl)alkyl tosylate partly lies in the structure of the major solvolysis product **324** (Scheme 79). Monitoring of the reaction by NMR spectroscopy allowed the observation of intermediate **327**, which was suggested to result from a successive ion pair formation, rearrangement, and internal return. It was then observed that **327** was progressively converted into **324** at a rate 3 times slower than when it was produced from **323**. From these observations, the authors concluded that the high reactivity of **323** was attributed to the σ-donation from the C3–C4 bond, allowing the positive charge to also be shared in the β-position of the CF_3_ group via intermediate **326**. Furthermore, the presence of a ground-state strain of approximately 6.5 kcal⋅mol^−1^ due to the presence of the CF_3_ group was established in **323**, and the relief of this intrinsic strain in the transition state would act as an additional driving force and accelerate this reaction.

**Scheme 80:**
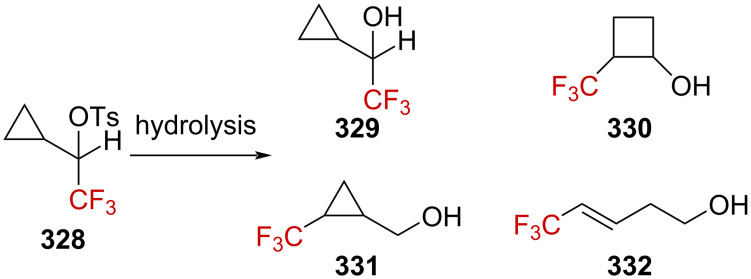
Products formed by the hydrolysis of **328**.

The solvolysis of cyclopropyl-substituted α-(trifluoromethyl) tosylate **328** was investigated by Meyer and Hanack, who reported a high tendency of **328** for rearrangements [177]. Hence, the hydrolysis of **328** led to **329** and to a mixture of the rearranged products **330**–**332** (Scheme 80).

**Scheme 81:**
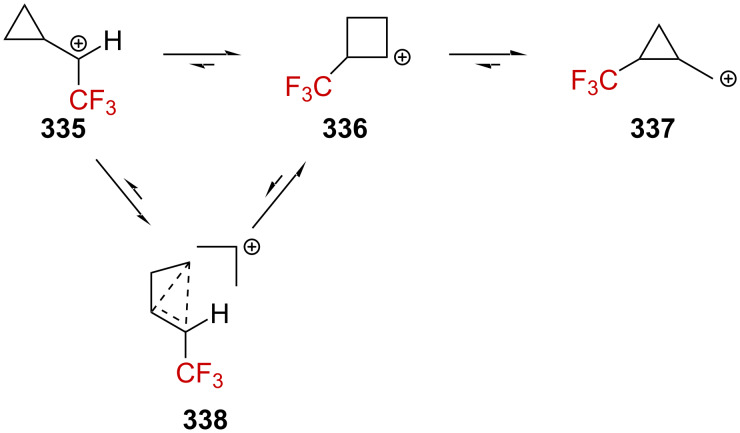
Proposed carbenium ion intermediates in an equilibrium during the solvolysis of tosylates **328**, **333**, or **334**.

Suspecting that **330** and **331** were obtained from the solvent trapping of the rearranged carbenium ions **336** and **337**, respectively (Scheme 81), the cyclobutyl tosylate **333** and the cyclopropyl tosylate **334** were also solvolyzed (Table 3). Interestingly, while **328** yielded 3.5% of the direct solvent-substituted product **329**, **333**, and **334** yielded 25% of **330** and 92% of **331**, respectively, as a result of the lower tendency to rearrange, due to the higher ion stability.

**Table 3:** Solvolysis products of compounds **328**, **333**, and **334**.

	**329**	**330**	**331**	**332**

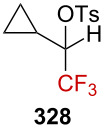	3.5%	28%	32%	34%
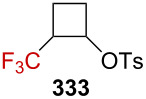	—	25%	68%	7%
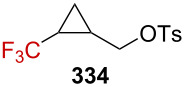	—	5%	92%	—

This suggests that **329** generates a highly reactive α-(trifluoromethyl)carbenium ion **335** upon solvolysis, which rapidly either rearranges via an alkyl shift to the β-(trifluoromethyl)carbenium ion **336** to give **330**, or to the γ-(trifluoromethyl)carbenium ion **338** via σ_C–C_ bond donation (i.e., a homoaromatic species), which is trapped at the primary carbon atom, similar as in norbornyl derivatives, to give **332**. Also, **336** can further rearrange by alkyl shift to give the γ-(trifluoromethyl)carbenium ion **337**, which leads to **331**. What is striking from these observations is the effect of the CF_3_ group on a positive charge nearby, as it continuously moves the latter from the α- to β- or eventually from the β- to the γ-position. Kinetic studies conducted by Roberts also support the formation of carbenium ion **335** as the rate-limiting step [178].

## Conclusion

Destabilized carbocations exhibit structural and electronic features that reduce their lifetimes. CF_3_-substituted carbocations are probably the cations that have long been regarded as the worst possible intermediates to be generated in an organic transformation, and therefore were deeply studied as exotic species. The study of CF_3_-substituted carbocations has therefore produced valuable contributions to understand their implications in synthetic transformations. Through these efforts, which are the subjects of this review, great perspectives in modern synthetic chemistry are expected as a result of the exploitation of these underestimated cationic intermediates.
